# Control-Anchored QUBO Clustering Reveals Control-Distant Postural Control Patterns in Motion Capture Balance Signals After COVID-19

**DOI:** 10.3390/s26206556

**Published:** 2026-10-15

**Authors:** Alisa Kunapinun, Andrzej Nowak, Peter J. Ellison, Mattan Pelah, Adar Pelah

**Affiliations:** 1 Harbor Branch Oceanographic Institute, Florida Atlantic University, Fort Pierce, FL 34946, USA; akunapinun@fau.edu or alisa3006@gmail.com; 2 Department of Psychology, Florida Atlantic University, Boca Raton, FL 33431, USA; nowak@fau.edu; 3 Center for Complex Systems, Florida Atlantic University, Boca Raton, FL 33431, USA; 4 School of Physics, Engineering & Technology, University of York, Heslington, York YO10 5DD, UK; peter.ellison@york.ac.uk; 5 Institute of Biomedical Engineering, Department of Engineering Science, University of Oxford, Oxford OX1 3PJ, UK; mattan.pelah@eng.ox.ac.uk; 6 Medical Research Council Brain Network Dynamics Unit, Nuffield Department of Clinical Neurosciences, University of Oxford, Oxford OX3 9DU, UK; 7 Department of Engineering and Computer Science, Florida Atlantic University, Boca Raton, FL 33431, USA

**Keywords:** post-COVID condition, balance, postural control, motion capture, frequency-domain analysis, vector-quantized variational autoencoder, QUBO, quantum annealing, weakly supervised clustering, control-anchored clustering

## Abstract

Post-COVID-19 balance and postural control alterations have been reported after SARS-CoV-2 infection, but individual-level phenotyping remains challenging because post-acute sequelae are heterogeneous and often lack clear clinical ground truths. We analyzed motion capture balance data from 140 participants, including 114 post-COVID-19 participants and 26 control-group participants, who completed sensory organization/postural balance tasks under single-task and cognitive dual-task conditions. Relative joint angle trajectories were transformed into 0–5 Hz frequency-domain spectra, from which 13 balance-derived task contrasts were constructed to capture cognitive dual-task versus single-task differences, closed-eyes versus open-eyes task condition differences, and related task condition changes. Joint-wise spectral contrast features were encoded using a vector-quantized variational autoencoder. Control-distant subgroup discovery was then formulated as a control-anchored, graph-based QUBO clustering problem and solved using a quantum annealing workflow. Simulated annealing and tabu search were applied to the identical BQM/QUBO formulation as classical solver-level references, whereas Gaussian mixture modeling was retained as an unconstrained classical clustering reference. Consistent with the control-anchored design of the QUBO objective, QUBO clustering identified a selective subset of post-COVID-19 participants with control-distant latent balance profiles while largely preserving the control-group reference structure. Across all task contrasts, quantum annealing assigned 1 of 307 control-group participant–contrast assignments (0.3%) and 118 of 1366 post-COVID-19 participant–contrast assignments (8.6%) to the control-distant group. Simulated annealing and tabu search produced slightly larger but similar control-distant post-COVID-19 subsets, assigning 143 of 1366 post-COVID participant–contrast assignments (10.5%) and 0 of 307 control-group participant–contrast assignments (0.0%) to the control-distant group. In contrast, GMM assigned 63 of 307 control-group participant–contrast assignments (20.5%) and 266 of 1366 post-COVID-19 participant–contrast assignments (19.5%) to the control-distant group. Parameter sensitivity analysis showed high overall assignment agreement for the same BQM/QUBO formulation, but the exact membership of the sparse control-distant subset was more variable. Simulated annealing sensitivity analysis showed overall agreement of 98.3–100.0% and control-distant Jaccard indices of 0.836–1.000 relative to the baseline, whereas a representative QPU sensitivity analysis showed overall agreement of 93.6–100.0% and control-distant Jaccard indices of 0.443–1.000. Frequency band profiles further suggested that QUBO-derived control-distant assignments corresponded to contrast-dependent and exploratory interpretable postural control patterns, particularly in low-frequency postural sway-dominant and mid–low-frequency postural control bands. These findings support the feasibility of objective-driven, control-anchored QUBO-based quantum-assisted clustering for exploratory subgroup discovery in heterogeneous, label-limited biomedical datasets. The proposed framework does not establish diagnostic criteria for long COVID or evidence of quantum speedup but provides a hypothesis-generating approach for interpretable post-COVID-19 neuromotor phenotyping and related medical data analysis applications.

## 1. Introduction

Since the emergence of the COVID-19 pandemic, hundreds of millions of individuals worldwide have been affected by SARS-CoV-2. Although many patients recover from the acute phase of infection, a substantial proportion experience persistent symptoms that extend well beyond the initial illness. This condition, commonly referred to as long COVID or post-acute sequelae of COVID-19 (PASC), has been formally defined as the presence of new, returning, or ongoing symptoms several months after the acute infection [1]. Epidemiological and biological studies indicate that long COVID affects a substantial subset of individuals after COVID-19 and may involve persistent multisystem abnormalities rather than a single disease mechanism [2,3]. A defining challenge of long COVID is its marked heterogeneity, with symptoms spanning fatigue, cognitive dysfunction, autonomic disturbances, sensory complaints, and neuromotor impairments [2]. This heterogeneity complicates objective identification, particularly when impairments are subtle, intermittent, or compensated by adaptive motor strategies. As a result, some individuals may perceive themselves as clinically recovered despite residual balance or postural control abnormalities that can be detected using objective assessment [4,5].

Postural control provides a sensitive window into the interaction among visual, vestibular, proprioceptive, neuromuscular, and cognitive systems. Human balance is regulated through coordinated sensory integration and motor control mechanisms that stabilize the body during standing and movement [6]. Measures of postural steadiness have long been used to quantify balance differences across healthy adults, aging populations, and individuals with neurological or sensorimotor impairments [7]. In the context of COVID-19, emerging evidence suggests that an infection history may be associated with changes in motor control, balance function, and movement coordination even after apparent clinical recovery [4,5,8]. Balance testing under sensory or cognitive perturbation may therefore reveal latent neuromotor deficits that are not readily apparent during routine clinical evaluation.

Despite this potential, translating balance and motion capture measurements into reliable individual-level markers of post-COVID-19 dysfunction remains challenging. Conventional balance metrics are influenced by substantial inter-individual variability, including differences in body morphology, habitual movement strategies, baseline postural sway, and compensatory control [6,7]. These factors can obscure subtle disease-related effects, causing many studies to detect differences at the group level while leaving individual-level separation unresolved. This limitation is particularly important in long COVID, where control-distant neuromotor patterns may occur only in a subset of individuals rather than uniformly across the post-COVID-19 population.

Frequency-domain analysis offers an alternative representation of postural control dynamics by characterizing movement signals according to their spectral content. The spectral analysis of postural sway can capture changes in balance stability that may not be evident from time-domain measures alone [9]. Frequency-based analysis has also been used to interpret tremor-related movement components and distinguish low-frequency postural sway from higher-frequency oscillatory activity [10]. Our previous work showed that joint-wise spectral representations derived from inverse kinematics can reveal task-dependent group-level differences between post-acute COVID-19 and non-COVID-19 individuals, particularly under cognitive perturbation [11]. However, spectral analysis alone does not determine whether these group-level differences reflect a uniform post-COVID-19 effect or distinct latent phenotypes within a heterogeneous population.

In the absence of a reliable clinical ground truth for post-COVID-19 neuromotor impairment, supervised classification approaches are inherently limited. The problem is better framed as phenotype discovery, in which latent subgroups are identified from high-dimensional data without diagnostic ground truth labels. Unsupervised and weakly supervised learning approaches have been widely used in biomedical research to discover computational phenotypes, latent disease clusters, and patient subgroups from noisy, sparse, or heterogeneous clinical data [12,13,14]. Autoencoder-based representation learning is well suited to this setting because it can compress high-dimensional observations into lower-dimensional latent spaces while preserving the salient structure. Vector-quantized variational autoencoders (VQ-VAEs) extend this idea by learning discrete latent codebooks rather than purely continuous latent variables [15]. Discrete latent representations can support the aggregation of recurring patterns and provide an interpretable interface for the downstream clustering of complex time-series or motion-derived data [16,17].

Classical clustering methods provide useful baselines for latent subgroup discovery but may impose assumptions that are not well matched to heterogeneous clinical data. Gaussian mixture modeling (GMM), for example, represents the latent structure as a mixture of Gaussian components and is therefore influenced by the geometry and covariance structure of the latent space [18]. In post-COVID-19 balance phenotyping, this can be problematic because the goal is not merely to partition the latent space geometrically but to identify control-distant subgroups while preserving the structure of the control-group reference. A clustering objective that explicitly incorporates relational structure and clinical assumptions may therefore be more appropriate for label-limited biomedical phenotyping.

Quantum computing is increasingly being explored as an emerging computational paradigm in biomedical science, with potential applications in drug discovery, molecular simulation, bioinformatics, genomics, multi-omics integration, and precision medicine [19,20]. Recent work has demonstrated proof-of-concept biomedical applications of quantum-enhanced algorithms, including molecular and drug discovery workflows [21]. However, many medical applications of quantum computing remain at an early translational stage, and current studies should be framed cautiously in terms of potential rather than established clinical acceleration [19]. In this context, the value of quantum-assisted methods is not limited to the hardware speed but also includes the ability to formulate clinically meaningful problems as structured optimization objectives.

Quadratic unconstrained binary optimization (QUBO) provides a natural framework for encoding clustering and subgroup discovery as an explicit energy minimization problem. QUBO and related Ising formulations represent combinatorial optimization problems using binary decision variables and pairwise interactions [22,23]. Quantum annealing provides a hardware-assisted approach to sampling low-energy configurations of QUBO objectives, and recent studies have explored its use in optimization, clustering, scheduling, and graph-structured decision problems [24,25,26]. For biomedical phenotyping, a QUBO formulation is particularly attractive because the prior structure can be encoded directly into the objective function. In the present setting, this allows control anchoring, the sparsity of control-distant assignments, outlier preference, and graph smoothness over latent similarity relationships to be incorporated into a single optimization problem.

In this study, we propose a balance-focused, quantum-assisted framework for exploring post-COVID-19 neuromotor phenotypes from motion capture data. Participants completed seven sensory organization and postural balance tasks under single-task and cognitive dual-task conditions. Relative joint angle trajectories were transformed into 0–5 Hz frequency-domain spectra, from which 13 balance-derived task contrasts were constructed to capture cognitive dual-task versus single-task differences, closed-eyes versus open-eyes task condition differences, and related task condition changes. Full 128-point joint-wise spectral contrast representations were encoded using a VQ-VAE to obtain latent neuromotor representations. We then formulated control-distant subgroup discovery as a control-anchored, graph-based QUBO clustering problem and solved it using a quantum annealing workflow. Simulated annealing and tabu search were applied to the same BQM/QUBO formulation as classical same-objective solver-level references, whereas GMM was retained as an unconstrained geometry-driven clustering reference.

This work is exploratory and does not aim to establish diagnostic criteria for long COVID or to determine infection status. Instead, the goal is to test whether latent frequency-domain balance representations can be combined with a control-anchored QUBO objective to identify interpretable control-distant post-COVID-19 balance phenotypes under label-limited and heterogeneous clinical conditions. Because latent clustering assignments are difficult to interpret physiologically on their own, we further summarized the joint-wise spectral contrast maps as predefined frequency bands to examine whether control-distant subgroups showed interpretable postural control patterns. This band-wise analysis served as an explanatory layer linking the optimization-derived subgroup assignments to low-frequency sway, postural control, compensatory, and tremor-like/high-frequency components.

By framing post-COVID-19 balance phenotyping as a control-anchored graph optimization problem, this study provides an early step toward objective-driven quantum-assisted medical data analysis for individualized subgroup discovery. The resulting participant-level assignments were further examined using demographic information, available clinical notes, and frequency-band spectral profiles to evaluate whether the derived subgroups were clinically contextualized and physiologically interpretable. The proposed framework is intended as a hypothesis-generating phenotyping approach rather than a diagnostic model.

## 2. Methodology

### 2.1. System Overview

**Figure 1:**
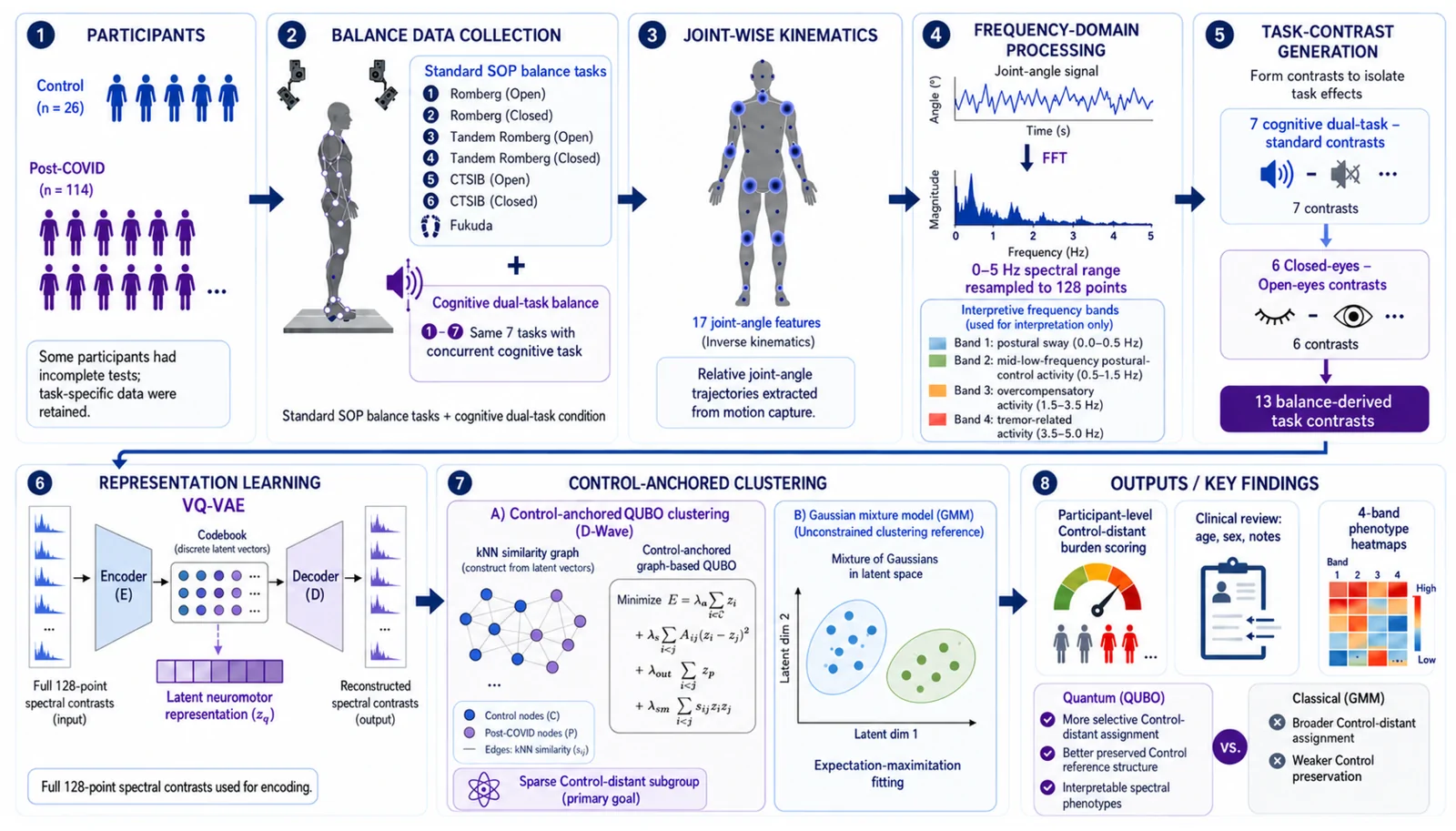
Overall framework for balance-based post-COVID-19 phenotyping using VQ-VAE and control-anchored QUBO clustering. Motion capture data from control-group and post-COVID-19 participants were collected during single-task sensory organization/postural balance tasks and cognitive dual-task balance conditions. Relative joint angle trajectories were extracted using inverse kinematics and transformed into 0–5 Hz frequency-domain spectral representations. Thirteen balance-derived task contrasts were constructed to capture cognitive dual-task versus single-task differences, closed-eyes versus open-eyes task condition differences, and related task condition changes. Full 128-point joint-wise spectral contrast representations were encoded using a vector-quantized variational autoencoder (VQ-VAE) to obtain latent neuromotor representations. These latent representations were analyzed using a control-anchored graph-based QUBO clustering objective solved through a quantum annealing workflow and classical same-BQM solvers, with Gaussian mixture modeling (GMM) used as an unconstrained classical clustering reference. Outputs included participant-level control-distant burden scoring, exploratory clinical review, and four-band spectral phenotype heatmaps for physiological interpretation. In the schematic, “standard” refers to the single-task SOP balance condition, whereas “cognitive dual-task” refers to the same balance tasks performed with a concurrent cognitive task.

This study uses a control-anchored weakly supervised analytical framework to identify balance-related neuromotor phenotypes from motion capture-derived joint kinematic signals in a heterogeneous post-COVID-19 cohort. An overview of the complete workflow is shown in Figure 1. The pipeline begins with single-task sensory organization/postural balance tasks and cognitive dual-task balance conditions collected from control-group and post-COVID-19 participants. Relative joint angle trajectories are extracted using inverse kinematics and transformed into 0–5 Hz frequency-domain spectral representations.

From these spectral representations, 13 balance-derived task contrasts are constructed to capture cognitive dual-task versus single-task differences, closed-eyes versus open-eyes task condition differences, and related task condition changes. Full 128-point joint-wise spectral contrast representations are encoded using a vector-quantized variational autoencoder (VQ-VAE) to obtain compact latent neuromotor representations. These latent representations are then analyzed using a control-anchored graph-based QUBO clustering objective solved through a quantum annealing workflow and through classical simulated annealing and tabu search solvers applied to the same BQM/QUBO formulation. Gaussian mixture modeling (GMM) is used as an unconstrained classical clustering reference to evaluate whether similar control-distant assignments arise from geometry-driven latent space clustering alone.

The resulting participant–contrast cluster assignments are aggregated into participant-level control-distant burden scores and interpreted using available demographic and clinical information, together with four-band spectral phenotype heatmaps. The framework is designed as an exploratory, hypothesis-generating approach for discovering control-distant balance phenotypes using a control group-anchored framework under label-limited and heterogeneous clinical conditions. It is not intended to establish diagnostic criteria for long COVID or to classify infection status.

### 2.2. Software and Hardware Environment

Computational analyses were performed on a Windows 11 workstation equipped with an Intel Core i9-14900KF CPU, 64 GB RAM, and an NVIDIA RTX 4090 GPU. The analysis pipeline was implemented in Python. Data preprocessing, numerical analysis, and tabular summaries used standard scientific Python libraries, including NumPy, SciPy, pandas, and scikit-learn. VQ-VAE model training was implemented in PyTorch and performed locally using GPU acceleration. PCA and Gaussian mixture modeling were implemented using scikit-learn. BQM/QUBO construction and solver workflows were implemented using D-Wave Ocean tools, with quantum annealing samples obtained remotely through the D-Wave cloud quantum annealing service. Simulated annealing and tabu search were run as classical same-BQM solver references. Hardware and software details are provided for reproducibility context; runtime was not used as a primary outcome or as evidence of quantum speedup.

### 2.3. Data Acquisition and Study Tasks

Motion capture data were acquired during balance assessments using a unified sensing and recording protocol to ensure consistent measurement conditions across task conditions. A markerless Microsoft Kinect-series depth camera system with three-dimensional body tracking was used to capture full-body kinematic data. The depth/body-tracking stream was acquired at approximately 30 frames per second using the Azure Kinect depth camera configuration of 640 × 576 pixels. The analytical pipeline used the exported three-dimensional skeletal joint trajectories rather than raw RGB or depth images. The camera was positioned in front of the participants to maintain full-body visibility during standing balance tasks, and participants were instructed to remain within the camera capture volume throughout each trial.

Because the analysis was based on markerless depth camera-derived skeletal trajectories, the recorded joint positions should be interpreted as estimated kinematic trajectories rather than direct anatomical marker measurements. The spatial resolution of the depth/body-tracking stream and the skeletal fitting procedure can introduce millimeter-scale positional uncertainty, and this uncertainty can propagate to relative joint angle estimates, particularly when two adjacent segment endpoints are displaced in opposite directions. Consequently, small angle differences between task conditions may partly reflect measurement uncertainty, tracking variability, or skeletal-model fitting errors. To reduce the influence of absolute positional offsets, the analysis used relative joint angle trajectories, trial-level centering, frequency-domain summaries, and group/subgroup-level spectral profiles rather than interpreting isolated frame-level angle differences. Nevertheless, Kinect-derived joint angle estimates remain less precise than marker-based motion capture measurements, and this limitation should be considered when interpreting small single-task versus cognitive dual-task differences.

Inverse kinematic features were derived from the exported three-dimensional skeletal joint coordinates by forming vectors between anatomically connected joint pairs along the Kinect skeletal chain. For each retained joint pair, a proximal-to-distal segment vector was computed at each frame, and roll- and pitch-related angular components were estimated from the segment orientation over time. These roll and pitch components were then combined into a vector-magnitude motion signal for each joint pair before frequency-domain transformation. Yaw-related components were not retained because they were more sensitive to the participant orientation relative to the camera and were less central to the standing balance tasks analyzed here. The resulting signals were therefore treated as relative joint angle motion features rather than precise anatomical joint angle measurements, following our previous joint-wise spectral balance analysis implementation [11].

All experimental procedures were approved by the Institutional Review Board of Florida Atlantic University (IRB No. 1423095-9), and written informed consent was obtained from all participants prior to data collection, in accordance with the Declaration of Helsinki. This study did not involve a clinical trial; therefore, clinical trial registration was not applicable.

COVID-19 status was determined from available study records, laboratory testing documentation, and clinical information. Participants classified as post-COVID-19 had a documented prior SARS-CoV-2 infection confirmed using clinically accepted COVID-19 testing, including RT-PCR or clinically validated antigen testing. All post-COVID-19 participants included in the analysis cohort had a documented positive COVID-19 test before enrollment in the balance assessment study. Where available, diagnosis dates and physician confirmation were recorded. Among the post-COVID-19 participants, one individual had a physician-documented diagnosis of long COVID established at a specialty clinic. Participants were included in the post-COVID-19 group only after documented recovery from the acute phase of infection.

Participants in the control group were defined as individuals without a known history of diagnosed COVID-19. Among the 26 control participants in the analysis cohort, 22 had documented negative COVID-19 testing at study enrollment, whereas 4 reported no known prior COVID-19 history but did not have prior diagnostic confirmation available. Serological antibody testing was available for a subset of participants but was not required for group assignment. COVID-19 classification was used for descriptive and comparative purposes only and was not used as a ground truth label in the control-anchored weakly supervised analytical framework.

For each participant, three-dimensional joint position trajectories (*x*, *y*, *z*) were recorded for major body segments, including the pelvis, spine, neck and head, upper extremities, lower extremities, and selected facial landmarks. Joint positions were captured bilaterally for the left and right body sides where applicable. The motion capture system recorded kinematic data at approximately 30 frames per second, corresponding to an effective sampling interval of approximately 0.033 s. Each balance trial followed the standardized Gans Sensory Organization Performance (SOP) balance protocol and was designed to last 30 s, corresponding to approximately 900 frames per trial before any trimming or exclusion due to incomplete recordings. The final number of retained frames could vary slightly across participants and task conditions because of acquisition timing, missing frames, or incomplete trials. All recordings were processed using the same temporal resolution across participants and task conditions.

Each participant was asked to complete seven sensory organization and postural balance tasks in the following order: Romberg with eyes open, Romberg with eyes closed, Tandem Romberg with eyes open, Tandem Romberg with eyes closed, Clinical Test of Sensory Interaction on Balance (CTSIB) with eyes open, CTSIB with eyes closed, and Fukuda. These tasks were designed to probe postural stability, sensory integration, visual dependence, and compensatory balance control under varying task demands. The same seven tasks were then repeated under a cognitive dual-task balance condition. During this condition, participants performed the same standing balance tasks while responding verbally to a concurrent randomly generated cognitive dual-task paradigm. In eyes-open sessions, the cognitive perturbation included a Stroop color naming task. In eyes-closed sessions, auditory prompts were used, such as asking participants to name a city beginning with a specified letter. This protocol allowed a comparison between single-task balance responses and cognitive dual-task balance responses, while recognizing that the cognitive dual-task conditions involved concurrent cognitive task demands.

The final analysis cohort included 140 participants with valid balance task data, consisting of 26 control participants and 114 post-COVID-19 participants. Some participants did not complete every task condition; therefore, participants with incomplete task data were retained for task-specific analyses to avoid the unnecessary loss of available data. Participant-level control-distant burden scores were subsequently normalized by the number of available task contrasts for each participant.

Basic demographic and clinical metadata, including age, sex, and available clinical notes, were used for descriptive and exploratory interpretation only and were not included as inputs to the analytical pipeline. Participant demographics for the analysis cohort are summarized in Table 1.

### 2.4. Joint-Wise Spectral Representation for Balance Analysis

This subsection describes the transformation of motion capture-derived kinematic signals into joint-wise spectral representations for balance analysis. Three-dimensional joint trajectories were converted into relative joint angle signals between connected body segments along the kinematic chain, allowing the representation to capture joint-level motion rather than the absolute camera-space position. Each joint signal was then transformed into the frequency domain within the 0–5 Hz range. The resulting spectral representations were resampled to a fixed length of 128 points for VQ-VAE latent representation learning and downstream clustering.

#### 2.4.1. Motion Signal Preprocessing and Quality Control

Before spectral transformation, exported three-dimensional skeletal joint trajectories were inspected and processed using a consistent preprocessing pipeline across participants and task conditions. Trials with incomplete recordings or tracking failures that prevented the reconstruction of the required joint angle signals were excluded from the corresponding task-specific analysis. Participants with missing task conditions were retained for other available contrasts, and participant-level control-distant burden scores were later normalized by the number of available task contrasts.

For retained trials, short gaps in skeletal tracking were handled by the temporal interpolation of the affected joint trajectories before joint angle computation. Interpolation was applied only to brief missing-frame segments for which the surrounding trajectory remained continuous. Longer tracking losses, severe body-tracking failures, or trials with insufficient usable frames were treated as incomplete recordings and were not included for that task contrast. This procedure allowed brief acquisition interruptions to be handled without discarding otherwise usable trials, while avoiding the reconstruction of trials with substantial tracking artifacts.

Relative joint angle signals were computed from the interpolated skeletal trajectories and then converted into roll and pitch vector-magnitude time series as described below. To reduce the influence of static posture offsets and between-participant baseline differences, each trial-level joint angle signal was centered before frequency-domain transformation. No additional task-specific band-pass filtering was applied before the DFT; instead, spectral components were computed from the processed joint angle signals, and the resulting magnitude spectra were restricted to the 0–5 Hz range. Frequencies above 5 Hz were excluded from the retained representation because they were more likely to reflect tracking noise, non-voluntary high-frequency fluctuations, or task-specific artifacts than postural control dynamics.

No windowing-based frequency resolution enhancement was used to reinterpret the recordings beyond their duration-limited spectral resolution. The 0–5 Hz spectra were subsequently resampled to a fixed 128-point representation only to provide consistent input dimensionality for VQ-VAE encoding and downstream clustering. Therefore, the resampled spectral points were treated as machine learning features rather than independent physical frequency resolution bins.

#### 2.4.2. Frequency-Domain Joint Representation

For each trial, relative joint angle signals were derived from pairs of connected landmarks along the anatomical kinematic chain, including the axial skeleton, upper extremities, and lower extremities. For each retained connected joint pair, a three-dimensional segment vector was computed from the proximal joint to the distal joint at each time point. Relative segment motion was then represented using the roll and pitch components derived from the time-varying segment orientation. These roll and pitch components were combined into a vector-magnitude motion signal for each joint pair before spectral transformation. This inverse kinematic representation emphasized relative motion between connected body segments rather than the absolute camera-space position. Yaw was excluded because it was not essential for the balance tasks analyzed in this study and could have introduced additional variability related to the participant orientation rather than postural control dynamics.

For each joint, the resulting motion signal was transformed into the frequency domain using the discrete Fourier transform (DFT). Spectral representations were retained within the 0–5 Hz range, corresponding to physiologically relevant frequencies for postural control and corrective balance responses. Frequencies above 5 Hz were excluded because they were considered more likely to reflect noise or non-voluntary high-frequency fluctuations than meaningful postural control dynamics. To obtain a fixed-dimensional representation across trials of varying duration, the 0–5 Hz spectral range was resampled to 128 points. This resampling was used to provide a consistent input size for the VQ-VAE and should not be interpreted as producing 128 independent frequency resolution bins. The effective spectral resolution was determined by the trial duration rather than by the number of resampled feature points [27,28]. For trials of approximately 30 s, the lowest reliably interpretable oscillatory frequency is on the order of 0.067 Hz when requiring at least two complete cycles for periodic interpretation. Therefore, low-frequency components below the duration-limited resolution were interpreted cautiously, and frequency band summaries were used for physiological interpretation rather than individual bin-level interpretation.

For each task condition, the raw joint-wise spectral magnitude for joint *j* and resampled spectral point *f* was denoted as $S_{j,f}$. Balance-derived task contrasts were computed by subtracting the reference-condition spectrum from the target-condition spectrum:

(1) $$\Delta S_{j,f}=S_{j,f}^{\mathrm{target}}-S_{j,f}^{\mathrm{reference}}.$$

The resulting contrast map was then normalized independently for each joint using the sum of the absolute contrast magnitudes across the 128 resampled spectral points:

(2) $$\Delta {\tilde{S}}_{j,f}=\frac{\Delta S_{j,f}}{\sum_{f^{\prime }=1}^{128}\left|\Delta S_{j,f^{\prime }}\right|+\epsilon }.$$

where $S_{j,f}^{\mathrm{target}}$ and $S_{j,f}^{\mathrm{reference}}$ denote the raw spectral magnitudes for the target and reference task conditions, respectively; $\Delta S_{j,f}$ denotes the raw spectral contrast; $\Delta {\tilde{S}}_{j,f}$ denotes the normalized spectral contrast representation; and ϵ is a small constant used to avoid division by zero. This normalization was applied after contrast computation, thereby preserving the direction of spectral modulation between task conditions while reducing scale differences across participants, joints, and trials.

**Figure 2:**
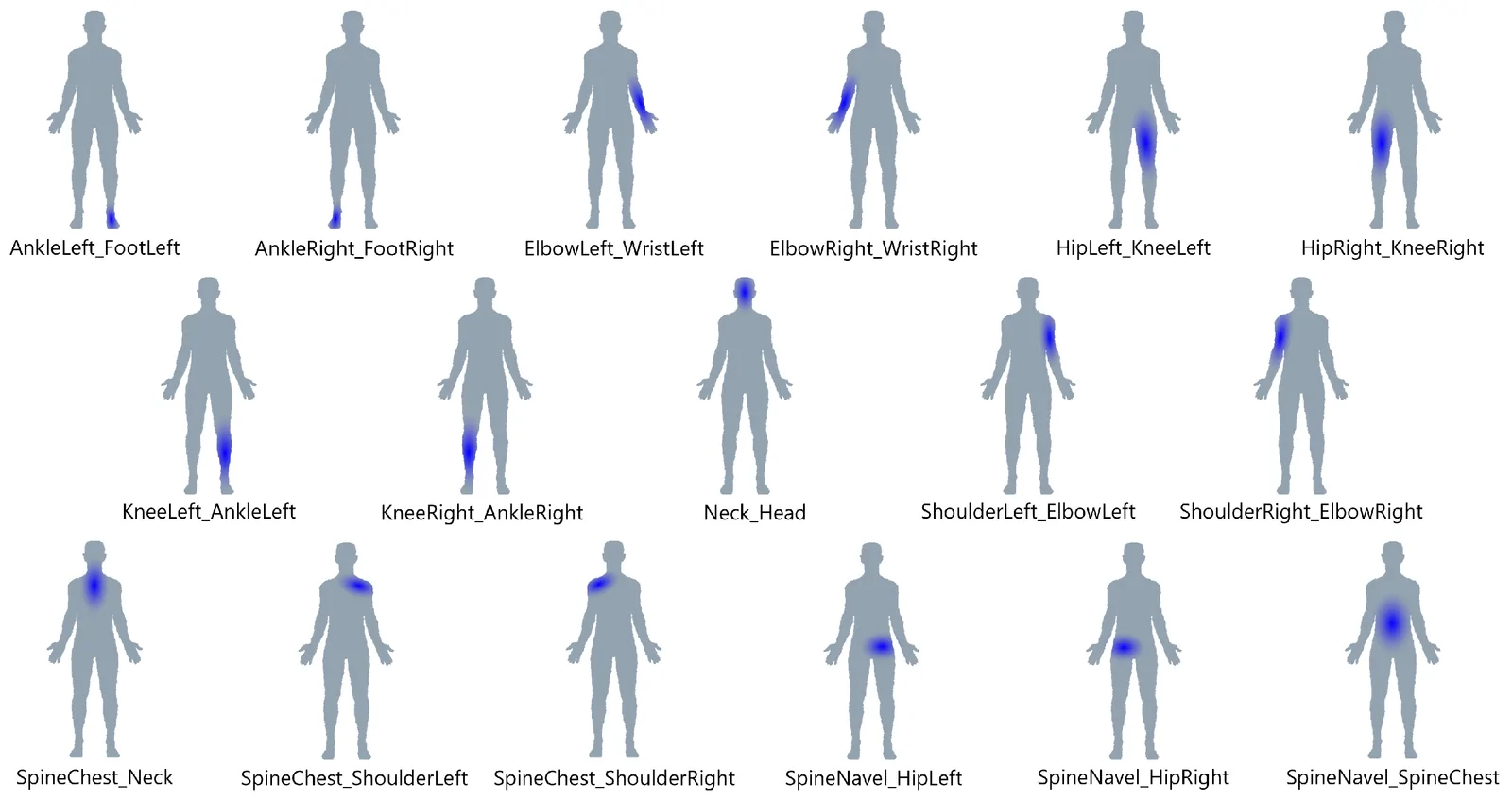
Seventeen joint-wise angle features used for spectral balance analysis. Each silhouette highlights one connected joint pair used to compute a relative joint angle signal. The retained features span distal lower-limb, knee, hip, upper-limb, shoulder, head–neck, and trunk-related segments. Roll and pitch components were combined into a vector-magnitude representation for each joint pair before spectral analysis.

Based on the anatomical kinematic chain, a total of 17 joint-wise angle features were retained for subsequent analysis. The retained joint angle features were AnkleLeft–FootLeft, AnkleRight–FootRight, ElbowLeft–WristLeft, ElbowRight–WristRight, HipLeft–KneeLeft, HipRight–KneeRight, KneeLeft–AnkleLeft, KneeRight–AnkleRight, Neck–Head, ShoulderLeft–ElbowLeft, ShoulderRight–ElbowRight, SpineChest–Neck, SpineChest–ShoulderLeft, SpineChest–ShoulderRight, SpineNavel–HipLeft, SpineNavel–HipRight, and SpineNavel–SpineChest. These features spanned the trunk, head, upper extremities, lower extremities, and major joints involved in balance control. The resulting representation formed a structured joint-wise spectral contrast map with joint and resampled frequency dimensions, which was used for VQ-VAE latent encoding and downstream clustering. The retained joint-wise features are illustrated in Figure 2.

#### 2.4.3. Cognitive Dual-Task Versus Single-Task Contrasts

To characterize task condition differences associated with the cognitive dual-task balance condition, cognitive dual-task versus single-task contrasts were constructed for all seven balance tasks: Romberg with eyes open, Romberg with eyes closed, Tandem Romberg with eyes open, Tandem Romberg with eyes closed, CTSIB with eyes open, CTSIB with eyes closed, and Fukuda. For each task, the raw joint-wise spectral representation obtained during the single-task condition was subtracted from the corresponding representation obtained during the cognitive dual-task balance condition. The resulting contrast map was then normalized at the joint level as described above.

The cognitive dual-task-related raw contrast was defined as

(3) $$\Delta S_{\mathrm{dual}-\mathrm{single}}=S_{\mathrm{dual}}-S_{\mathrm{single}},$$

where $S_{\mathrm{dual}}$ denotes the raw joint-wise spectral representation during the cognitive dual-task balance condition, and $S_{\mathrm{single}}$ denotes the corresponding raw spectral representation during the single-task balance condition. The normalized contrast representation, ${\tilde{\Delta S}}_{\mathrm{dual}-\mathrm{single}}$, was used as the input representation for downstream latent encoding. This procedure generated seven cognitive dual-task-related task contrasts and was designed to characterize spectral modulation associated with the cognitive dual-task balance condition relative to the single-task balance condition.

For visualization and physiological interpretation, contrast values can also be summarized as band-wise spectral differences:

(4) ΔEnergy=targetcondition−referencecondition.

For cognitive dual-task-related contrasts, the target condition corresponds to the cognitive dual-task balance condition, whereas the reference condition corresponds to the single-task balance condition for the same task. The term “reference condition” is used to avoid confusion with the control group.

#### 2.4.4. Closed-Eyes Versus Open-Eyes Task Contrasts

To characterize closed-eyes versus open-eyes task condition differences, closed-eyes versus open-eyes contrasts were constructed for the three balance tasks that included paired eyes-open and eyes-closed conditions: Romberg, Tandem Romberg, and CTSIB. These contrasts were computed separately for single-task balance tasks and cognitive dual-task balance tasks.

For compact notation, the closed-eyes versus open-eyes contrast under the single-task condition was denoted as $\Delta S_{V,\mathrm{single}}$, where V denotes visual condition comparison and single indicates the single-task balance condition. This raw contrast was defined as

(5) $$\Delta S_{V,\mathrm{single}}=S_{\mathrm{Closed},\mathrm{single}}-S_{\mathrm{Open},\mathrm{single}},$$

where $S_{\mathrm{Closed},\mathrm{single}}$ and $S_{\mathrm{Open},\mathrm{single}}$ denote the raw spectral representations during the eyes-closed and eyes-open single-task balance conditions, respectively. The normalized contrast representation, ${\tilde{\Delta S}}_{V,\mathrm{single}}$, was used for downstream latent encoding.

For cognitive dual-task balance tasks, the corresponding closed-eyes versus open-eyes contrast was denoted as $\Delta S_{V,\mathrm{dual}}$, where dual denotes the cognitive dual-task balance condition. This raw contrast was defined as

(6) $$\Delta S_{V,\mathrm{dual}}=S_{\mathrm{Closed},\mathrm{dual}}-S_{\mathrm{Open},\mathrm{dual}},$$

where $S_{\mathrm{Closed},\mathrm{dual}}$ and $S_{\mathrm{Open},\mathrm{dual}}$ denote the raw spectral representations during the eyes-closed and eyes-open cognitive dual-task balance conditions, respectively. The normalized contrast representation, ${\tilde{\Delta S}}_{V,\mathrm{dual}}$, was used for downstream latent encoding. Under the cognitive dual-task condition, the eyes-open trials used Stroop color naming, whereas the eyes-closed trials used auditory verbal prompts. Therefore, $\Delta S_{V,\mathrm{dual}}$ reflects closed-eyes versus open-eyes task condition differences under different concurrent cognitive task formats rather than isolated visual input removal alone. This procedure produced six closed-eyes versus open-eyes contrasts. Together with the seven cognitive dual-task versus single-task contrasts, this yielded 13 balance-derived task contrasts for downstream latent representation learning and clustering.

For band-wise visualization, the target condition corresponds to the eyes-closed task, whereas the reference condition corresponds to the eyes-open task of the same balance condition. For single-task contrasts, this definition primarily reflects closed-eyes versus open-eyes differences. For cognitive dual-task contrasts, it reflects the combined difference between the eye condition and concurrent cognitive task format, as described above. The term “reference condition” is used to avoid confusion with the control group.

Although the full joint-wise spectral contrast maps resampled to 128 points were used as inputs to the VQ-VAE and clustering pipeline, four frequency bands were also used for physiological interpretation and heatmap visualization: postural sway-dominant activity, 0.0–0.5 Hz; mid–low-frequency postural control activity, 0.5–1.5 Hz; corrective or overcompensatory activity, 1.5–3.5 Hz; and tremor-like/high-frequency components, 3.5–5.0 Hz. These band-wise summaries were used only for the interpretation of group-level and phenotype-level spectral patterns and were not used as the primary input representation for latent encoding.

For the frequency band profile summaries, the number of participants contributing to each subgroup was recorded separately for each task contrast, clustering method, and frequency band profile group. To quantify uncertainty in the band-averaged profiles, nonparametric bootstrap confidence intervals were computed for the mean ΔEnergy within each subgroup and frequency band. Bootstrap resampling was performed at the participant level within each subgroup using 5000 bootstrap resamples, and 95% confidence intervals were estimated from the empirical bootstrap distribution. These variability summaries were used only for the interpretation of the frequency band profiles and were not used as inputs to VQ-VAE encoding, QUBO clustering, simulated annealing, tabu search, or GMM clustering.

### 2.5. Discrete Latent Encoding via VQ-VAE

To move beyond descriptive spectral summaries and enable participant-level phenotype discovery, the joint-wise spectral contrast representations were encoded using a vector-quantized variational autoencoder (VQ-VAE). As summarized in the overall workflow in Figure 1, the VQ-VAE was used as a label-free representation learning module between the spectral contrast generation stage and the downstream clustering stage. For each balance-derived task contrast, the normalized 0–5 Hz spectral contrast representation was resampled to 128 points, ensuring consistent input dimensionality across joints, tasks, and participants. Each input sample therefore consisted of a joint-wise spectral contrast map derived from 17 anatomical joint angle features. For convolutional VQ-VAE encoding, the joint dimension was zero-padded to 32 channels, and the spectral dimension was represented using 128 resampled points, yielding an input tensor of size 1×128×32 for each participant–contrast sample. The additional padded joint positions did not correspond to anatomical joint features and were used only to provide a fixed rectangular input grid for convolutional encoding.

Briefly, the VQ-VAE architecture consisted of an encoder, a discrete codebook, and a decoder. The encoder mapped each normalized spectral contrast map to a continuous latent representation, which was then quantized by assigning each latent vector to its nearest codebook entry using the Euclidean distance. In this study, the latent embedding dimensionality was set to 64, and the codebook consisted of 128 learnable embedding vectors. The codebook vectors were initialized as trainable embedding parameters before optimization and were updated during training through the standard VQ-VAE vector quantization objective, which included an embedding loss for moving selected codebook vectors toward encoder outputs and a commitment loss that encourages encoder outputs to remain close to their selected codebook entries. The straight-through estimator was used so that gradients from the decoder reconstruction loss could be passed to the encoder while the nearest-neighbor codebook assignment remained discrete. Because the codebook contained 128 vectors in a 64-dimensional latent space, the entries were not constrained to be linearly independent; at most, 64 vectors could be linearly independent in this space. Instead, the codebook entries were interpreted as learned prototype vectors that discretized recurring spectral motion patterns for downstream phenotype discovery.

The decoder was trained jointly to reconstruct the original spectral contrast input from the quantized latent representation. The reconstruction objective encouraged the discrete codebook to preserve task-relevant spectral structure while reducing the dimensionality of the original joint-wise spectral representation. Compared with conventional autoencoders that rely on continuous latent variables, the discrete latent space induced by the VQ-VAE enables the aggregation of recurring spectral patterns and provides a structured representation for downstream control-anchored clustering [16].

In detail, the VQ-VAE used in this study was implemented as a convolutional encoder–quantizer–decoder architecture. The encoder consisted of three two-dimensional convolutional layers. The first layer mapped the single-channel input representation to 32 feature maps using a kernel size of 4, stride (2,1), and padding 1, followed by ReLU activation. The second layer mapped 32 feature maps to 64 feature maps using the same kernel size, stride, and padding, also followed by ReLU activation. The third layer mapped the 64 feature maps to a 64-channel latent embedding representation using a kernel size of 4, stride (2,1), and padding 1. This architecture downsampled the spectral dimension progressively while preserving the second spatial dimension of the joint-wise representation.

Vector quantization was then applied to the encoder output using a learnable codebook with 128 embedding vectors, each of dimension 64. For each latent vector, the nearest codebook entry was selected using the Euclidean distance. The vector quantization loss consisted of an embedding loss and a commitment loss, with commitment weight β=0.25, and the straight-through estimator was used to pass gradients from the decoder to the encoder.

The decoder mirrored the encoder using three transposed convolution layers. The first transposed convolution layer mapped the 64-channel quantized latent representation to 64 feature maps using kernel size 4, stride (2,1), and padding 1, followed by ReLU activation. The second layer mapped 64 feature maps to 32 feature maps using the same kernel size, stride, and padding, also followed by ReLU activation. The final transposed convolution layer mapped the 32 feature maps back to a single-channel reconstructed spectral contrast representation. The reconstruction loss was computed using the mean squared error, and the total training loss was the sum of the reconstruction loss and the vector quantization loss.

The VQ-VAE was trained using all available balance-derived spectral contrast maps pooled across the 13 task contrasts, because all contrasts shared the same joint-wise frequency-domain input structure. Training was performed using the Adam optimizer with an initial learning rate of $3\times 10^{-4}$, a batch size of 32, and 600 training epochs. A cosine annealing learning rate scheduler was used with $T_{\max}=500$ and a minimum learning rate of $1\times 10^{-5}$. No obvious codebook collapse was observed during training, indicating that the learned discrete codebook retained usable latent diversity for downstream clustering.

After VQ-VAE encoding, the discrete latent codes were aggregated to construct participant-level representations for each balance-derived task contrast. Specifically, the occurrence frequency of each codebook entry was counted across the encoded joint-wise spectral representation, yielding a fixed-length latent histogram for each participant and contrast. This histogram summarized the distribution of learned spectral patterns in the discrete latent space.

All encoded joint-wise spectral components were pooled into a single histogram without explicit joint weighting. This avoided imposing a priori assumptions about the relative importance of specific joints and allowed the representation to reflect the collective distribution of joint-level spectral patterns. Each histogram was normalized to sum to one, resulting in a probability distribution over codebook entries that was invariant to the number of encoded components available for a given participant and task contrast.

Because the VQ-VAE was used for label-free representation learning rather than supervised prediction, model evaluation was not based on classification accuracy or held-out label performance. Instead, the trained encoder and codebook were used to extract participant-level latent histograms for each balance-derived task contrast. These latent histograms served as task-specific neuromotor signatures and were exported for downstream clustering, including control-anchored QUBO clustering and Gaussian mixture modeling as an unconstrained classical clustering reference.

To assess representation-level stability, a post hoc VQ-VAE seed stability analysis was performed. The VQ-VAE was retrained using three random seeds while keeping the architecture and hyperparameters fixed, including latent dimension 64, codebook size 128, commitment weight β=0.25, batch size 32, learning rate $3\times 10^{-4}$, and 600 training epochs. Because VQ-VAE codebook indices are not identifiable across random initializations, codeword numbers were not compared directly across seeds. Instead, stability was assessed using the reconstruction error, codebook usage, codebook perplexity, and downstream control-distant assignment agreement after applying the same control-anchored BQM/QUBO construction to each seed-derived latent representation. Downstream stability was evaluated using both simulated annealing as a matched same-BQM reference and a parallel QPU-sampled workflow.

### 2.6. Control-Anchored Graph-Based QUBO Clustering

Following VQ-VAE encoding, participant-level latent representations were analyzed using a control-anchored graph-based QUBO clustering framework. QUBO and related Ising formulations provide standard representations for encoding combinatorial optimization problems as binary energy minimization objectives [22,23]. In this study, the goal was to identify control-distant latent profiles while preserving the control-group structure as a control-like reference. Clustering was performed independently for each of the 13 balance-derived task contrasts.

For each contrast, the VQ-VAE latent representation was first standardized and reduced using principal component analysis (PCA) [29]. PCA was applied after standardization, with the number of retained components selected automatically to preserve 95% of the explained variance. This PCA step was used as a hardware-aware preprocessing step to obtain a compact latent geometry for control distance scoring and graph construction. It was not intended to optimize clinical separation but to reduce the latent space dimensionality while preserving the dominant latent variance for QPU-compatible BQM construction.

A k-nearest-neighbor graph [30] was then constructed in the PCA-transformed latent space using k=32. The kNN graph was used as a hardware-aware sparsification strategy: rather than constructing a fully connected participant graph, which would introduce a quadratic number of pairwise interactions, only local latent-neighborhood relationships were encoded in the QUBO smoothness term. This reduced the BQM interaction density while preserving the local geometric structure in the latent space. Pairwise distances between neighboring samples were converted into RBF affinities,

(7) $$A_{ij}=\exp\left(-\frac{d_{ij}^{2}}{2\sigma^{2}}\right),$$

where $d_{ij}$ is the Euclidean distance between neighboring participants *i* and *j*, and σ is the median of the nonzero kNN distances for the corresponding contrast. Duplicate symmetric edges were merged by retaining the stronger affinity.

For each participant *i*, a binary variable $z_{i}$ was defined as

(8) $$z_{i}=\left\{\begin{array}{cc} 0, & \mathrm{control}\text{-}\mathrm{like},\\ 1, & \mathrm{control}\text{-}\mathrm{distant}. \end{array}\right.$$

To quantify the deviation from the control-group reference distribution, a control distance score was computed for each participant. For participant *i*, the raw control distance score was defined as the mean Euclidean distance from that participant to its nearest $k_{c}$ control-group participants in the PCA-transformed latent space, where $k_{c}=5$:

(9) $$r_{i}=\frac{1}{k_{c}}\sum_{j\in N_{k_{c}}^{\mathrm{Control}}\left(i\right)}{∥x_{i}-x_{j}∥}_{2},$$

where $x_{i}$ denotes the PCA-transformed latent representation of participant *i*, and $N_{k_{c}}^{\mathrm{Control}}\left(i\right)$ denotes the set of the $k_{c}$ nearest control-group participants to participant *i* in the PCA-transformed latent space. The raw control distance scores were then robustly normalized to the range [0,1] using the 5th and 95th percentiles:

(10) $$s_{i}=\mathrm{clip}\left(\frac{r_{i}-Q_{0.05}\left(r\right)}{Q_{0.95}\left(r\right)-Q_{0.05}\left(r\right)+\epsilon },0,1\right),$$

where $s_{i}$ denotes the normalized control distance score for participant *i*, $Q_{0.05}\left(r\right)$ and $Q_{0.95}\left(r\right)$ denote the 5th and 95th percentiles of the raw control distance scores, and ϵ is a small constant used to avoid division by zero. Higher values therefore indicate a greater latent space distance from the control-group reference distribution.

The QUBO objective was formulated as

(11) $$\begin{array}{cc} E= & \;\lambda_{\mathrm{anchor}}\sum_{i\in \mathrm{Control}}z_{i}+\lambda_{\mathrm{sparse}}\sum_{i\in \mathrm{COVID}}z_{i}-\lambda_{\mathrm{outlier}}\sum_{i\in \mathrm{COVID}}s_{i}z_{i}\\ \; & +\lambda_{\mathrm{smooth}}\sum_{(i,j)\in \mathrm{kNN}}A_{ij}{\left(z_{i}-z_{j}\right)}^{2}. \end{array}$$

In this objective, the first term penalizes the assignment of control-group participants to the control-distant group, thereby anchoring the control group to the control-like reference state. The second term imposes sparsity on control-distant assignments among post-COVID-19 participants. The third term rewards control-distant assignment for post-COVID-19 participants that are farther from the control-group reference distribution. The fourth term enforces graph smoothness by penalizing neighboring participants in the latent similarity graph when they are assigned different labels.

The QUBO weights were fixed as $\lambda_{\mathrm{anchor}}=8.0$, $\lambda_{\mathrm{sparse}}=1.0$, $\lambda_{\mathrm{outlier}}=3.0$, and $\lambda_{\mathrm{smooth}}=2.0$. These weights encode the intended behavior of the clustering objective: strong control-group anchoring, sparse control-distant assignment, preference for control-distant post-COVID-19 samples, and local consistency over the latent similarity graph.

The QUBO was implemented as a binary quadratic model (BQM), a form suitable for quantum annealing and related binary optimization workflows, including clustering and graph-structured optimization problems [24,25,26]. The graph smoothness term was expanded using the binary identity

(12) $${\left(z_{i}-z_{j}\right)}^{2}=z_{i}+z_{j}-2z_{i}z_{j},$$

which contributes linear biases to $z_{i}$ and $z_{j}$ and a quadratic interaction between the corresponding binary variables. Each contrast-specific BQM was solved using a D-Wave Advantage2 quantum annealing workflow through an embedding composite. For each BQM, 1000 reads were performed, and the chain strength was computed using uniform torque compensation. The QPU returned a set of sampled bitstrings, and the lowest-energy sampled bitstring was recorded as the QPU-sampled solution for downstream assignment summaries.

Because the all-control-like assignment, in which all binary variables are set to $z_{i}=0$, is feasible under the QUBO objective and has energy E=0, the QPU-sampled energy was also compared with this all-zero reference energy. This comparison was used only as an optimization quality reference to indicate whether the sampled QPU solution improved upon the trivial all-control-like assignment. It was not used to replace the reported QPU-sampled assignment summaries.

To assess QPU sampling stability, the QPU sampling procedure was repeated three times in the original analysis. The resulting sampled control-distant assignment patterns were unchanged across repeated runs, although the sampled energy values varied slightly. The sampled binary assignments were interpreted as contrast-specific control-like or control-distant labels and were subsequently aggregated across the 13 task contrasts to compute participant-level control-distant burden scores.

Runtime was not used as a primary outcome in this exploratory study. The main purpose of the quantum annealing workflow was to evaluate whether the control-anchored BQM/QUBO formulation could support sparse control-distant subgroup discovery, not to benchmark the end-to-end computational speed. The complete workflow included local preprocessing, VQ-VAE encoding, PCA transformation, control distance scoring, graph construction, BQM construction, remote QPU submission, minor embedding, network transfer, cloud queueing, QPU sampling, sample return, and local postprocessing. These components were not prospectively timed under a controlled benchmarking protocol. Therefore, the runtime is not used to support claims of quantum speedup, solver superiority, or an end-to-end runtime advantage. In this manuscript, the term “quantum-assisted” indicates that the control-anchored BQM/QUBO objective was sampled using a quantum annealing workflow, whereas the solver-level comparison focused on the assignment structure, control preservation, control-distant overlap, and achieved BQM energy under matched BQM/QUBO formulations.

### 2.7. Classical Same-BQM Solver References: Simulated Annealing and Tabu Search

To distinguish the effect of the control-anchored BQM/QUBO objective from the effect of the quantum annealing solver, we additionally solved the same contrast-specific BQM formulations using two classical heuristic solvers: simulated annealing and tabu search. Simulated annealing is a stochastic optimization method inspired by thermodynamic annealing and is widely used for approximating low-energy solutions to combinatorial optimization problems [31]. Tabu search is a local search metaheuristic that uses adaptive memory structures to discourage cycling and improve the exploration of discrete optimization landscapes [32].

For each of the 13 balance-derived task contrasts, the simulated annealing and tabu search solvers were applied to the identical BQM used for the quantum annealing workflow. Thus, the same binary variables, linear biases, quadratic interactions, kNN graph affinities, control distance scores, and QUBO weights were preserved across quantum annealing, simulated annealing, and tabu search. The resulting classical solver outputs therefore served as same-objective solver-level references, rather than as independent clustering models.

The same binary label interpretation was used for all three BQM-based solvers, with $z_{i}=0$ denoting control-like assignment and $z_{i}=1$ denoting control-distant assignment. For each classical solver and contrast, the lowest-energy solution returned by the solver was recorded as the solver-returned solution used for downstream same-BQM assignment summaries. As with the QPU workflow, the returned solution energy was compared with the all-control-like reference assignment with E=0 to aid the interpretation of the optimization quality. This reference comparison was used to identify whether the returned solution improved upon the trivial all-control-like reference, but it was not used to replace the reported solver-returned assignment summaries. These assignments were then aggregated into participant-level control-distant burden scores using the same scoring procedure described below.

These same-BQM solver references were used to assess whether the observed control-distant burden patterns were primarily determined by the control-anchored objective itself or depended strongly on the quantum annealing sampler. They are not used to claim quantum speedup or solver superiority. Instead, they provided a solver-level comparison under matched BQM/QUBO formulations, whereas GMM was retained separately as an unconstrained geometry-driven clustering reference.

#### Control Distance Threshold Baseline

To provide a simple objective-level baseline for the control-anchored QUBO formulation, we additionally evaluated a control distance thresholding approach. This baseline used the same normalized control distance score $s_{i}$ defined in Section 2.6 but did not include the QUBO sparsity term, outlier reward term, graph smoothness term, BQM optimization, quantum annealing, simulated annealing, or tabu search. It therefore served as a simple control distance-based reference for assessing whether the full QUBO objective produced assignment patterns beyond those obtained by thresholding the latent space distance from the control-group reference alone.

For each balance-derived task contrast, the control distance threshold was computed from the control-group participants only. Specifically, the 95th percentile of the normalized control distance scores among control-group participants was used as the contrast-specific threshold. Participants with $s_{i}$ greater than this threshold were assigned to the control-distant group, whereas all remaining participants were assigned to the control-like group. The threshold baseline was applied independently to each of the 13 task contrasts using the same VQ-VAE latent representation, PCA preprocessing, and control distance scores used in the QUBO workflow. The resulting binary assignments were aggregated into participant-level control-distant burden scores using the same scoring procedure described below.

### 2.8. Classical Clustering Reference: Gaussian Mixture Modeling

Gaussian mixture modeling (GMM) was used as a classical unconstrained clustering reference for the QUBO-based clustering framework. GMM provides a probabilistic mixture-model representation of latent structure and is commonly used for clustering in continuous feature spaces [18]. In this study, GMM was not intended to replace the control-anchored QUBO formulation or to serve as a same-objective solver-level reference. Rather, it provided a conventional geometry-driven clustering reference for assessing whether control-distant participant assignments could be obtained from the same VQ-VAE latent representations without graph-based binary optimization or explicit control anchoring. In contrast to simulated annealing and tabu search, GMM was not applied to the BQM/QUBO objective; it was retained as an unconstrained latent space clustering reference.

For each of the 13 balance-derived task contrasts, the VQ-VAE latent features were standardized using z-score normalization and fitted with a two-component GMM. Unlike the QUBO-based clustering procedure, no PCA reduction and no k-nearest-neighbor graph construction were applied prior to the primary GMM fitting. This choice was intentional because the VQ-VAE latent histograms already represented a learned feature extraction step, and the full 128-dimensional latent representation preserved the complete discrete codebook distribution available for classical clustering. The number of mixture components was fixed at two to provide a direct control-like versus control-distant comparison with the binary BQM-based assignments. A diagonal covariance structure was used to improve stability in the high-dimensional latent space relative to the number of participants. The GMM was fitted with 50 random initializations and covariance regularization of $10^{-5}$.

Because GMM cluster labels are arbitrary and do not directly encode control-like or control-distant status, clusters were mapped post hoc using their relationship with the control-group reference distribution. For each participant, a control distance score was computed in the standardized latent space as the mean Euclidean distance to the nearest five control-group participants, followed by robust normalization to the range [0,1] using the 5th and 95th percentiles. The GMM cluster with the higher mean control distance score was assigned as the control-distant cluster, and the remaining cluster was assigned as control-like.

For each participant and contrast, the fitted GMM therefore produced a binary control-like or control-distant assignment, together with the posterior probability of belonging to the control-distant component. These contrast-specific assignments were used for participant-level control-distant burden scoring in the same manner as the BQM-based assignments, allowing a comparison between the control-anchored BQM/QUBO objective and an unconstrained, geometry-driven classical mixture model.

As a preprocessing sensitivity check, GMM was also fitted after the same PCA transformation used for QUBO graph construction. This analysis did not improve control preservation: GMM-PCA assigned 182 of 307 control-group participant–contrast assignments (59.3%) and 831 of 1366 post-COVID-19 participant–contrast assignments (60.8%) to the control-distant component, compared with 63 of 307 control-group participant–contrast assignments (20.5%) and 266 of 1366 post-COVID-19 participant–contrast assignments (19.5%) for the primary GMM analysis. Therefore, the primary GMM reference analysis is reported using the full VQ-VAE latent representation, while the GMM-PCA sensitivity results were retained only as a preprocessing check.

### 2.9. Participant-Level Control-Distant Burden Scoring

After contrast-specific clustering, binary control-like and control-distant assignments were aggregated at the participant level. This aggregation was performed separately for the BQM-based solver outputs, including quantum annealing, simulated annealing, and tabu search, and for the GMM reference analysis. For each participant *p*, a control-distant flag was defined for each available task contrast *c* as

(13) $$a_{p,c}=\left\{\begin{array}{cc} 1, & \mathrm{if}\;\mathrm{participant}\;p\;\mathrm{was}\;\mathrm{assigned}\;\mathrm{to}\;\mathrm{the}\;\mathrm{control}\text{-}\mathrm{distant}\;\mathrm{group}\;\mathrm{in}\;\mathrm{contrast}\;c,\\ 0, & \mathrm{otherwise}. \end{array}\right.$$

Because not all participants completed all task contrasts, participant-level scores were normalized by the number of available contrasts. Let $N_{p}$ denote the number of task contrasts available for participant *p*, and let $A_{p}$ denote the number of control-distant contrast assignments:

(14) $$A_{p}=\sum_{c=1}^{N_{p}}a_{p,c}.$$

The normalized control-distant burden score was then computed as

(15) $${\mathrm{Score}}_{p}=\frac{A_{p}}{N_{p}}.$$

Thus, $A_{p}$ represents the control-distant contrast count, whereas ${\mathrm{Score}}_{p}$ represents the proportion of available task contrasts in which the participant was assigned to the control-distant group. This normalization allowed participants with incomplete task data to be retained while reducing bias due to differences in the number of completed contrasts. Participants with fewer available contrasts were interpreted cautiously because a small number of control-distant assignments could have a larger effect on the normalized score.

Participants were also assigned to operational control-distant burden groups based on the number and proportion of control-distant contrast assignments. Participants with no control-distant contrast assignments were labeled as control-like. Participants with one control-distant contrast assignment were labeled as having a single-test deviation. Participants with two control-distant contrast assignments were labeled as possible control-distant. Participants with three or more control-distant assignments and a control-distant burden score below 0.67 were labeled as probable control-distant. Participants with three or more control-distant assignments and a control-distant burden score of at least 0.67 were labeled as strong control-distant. These thresholds were prespecified for descriptive ranking and were not optimized against clinical outcomes. The labels were used as descriptive control-distant burden categories and were not intended to represent clinical diagnostic categories.

For participant ranking, the results were sorted primarily by the normalized control-distant burden score and then by the number of control-distant task contrasts. For the BQM-based solver outputs, additional tie-breaking was performed using the maximum and mean control distance scores. For the GMM reference analysis, ranking additionally incorporated the maximum and mean posterior probability of belonging to the control-distant GMM component. This ranking procedure was used to prioritize participants for an exploratory review of control-distant neuromotor patterns.

Demographic and clinical metadata were merged with the ranked participant-level results for post hoc interpretation. Available metadata included age, sex, clinical notes, COVID-19-related documentation, long-COVID or fatigue-related notes, balance-related notes, cardiovascular or hypertension-related notes, autoimmune history, orthopedic or surgical notes, and control-related notes. Age was calculated from the date of birth and record date when valid date information was available. These metadata were used only for the exploratory clinical review of the derived control-distant burden rankings and were not used as inputs to the VQ-VAE, BQM-based clustering, GMM clustering, or control-distant burden scoring procedures.

Per-contrast control-distant flags were also retained for all solver and clustering outputs to identify which task contrasts contributed to each participant’s control-distant burden score. This allowed the participant-level control-distant burden to be traced back to specific balance-derived contrasts while preserving the distinction between model-derived clustering assignments and clinical metadata.

## 3. Results

This section reports the balance-derived neuromotor patterns identified from normalized joint-wise spectral contrasts, VQ-VAE latent encoding, and downstream clustering. Analyses were performed using 13 balance-derived task contrasts constructed from single-task sensory organization/postural balance tasks and cognitive dual-task balance conditions. Because not all participants completed every task condition, the contrast-specific sample sizes varied across analyses. Across the 13 balance-derived task contrasts, the number of available samples ranged from 123 to 132 participants per contrast, with the control sample sizes ranging from 22 to 25 and post-COVID-19 sample sizes ranging from 101 to 108. Participant-level control-distant burden scores were therefore normalized by the number of available task contrasts for each participant, as described in the Materials and Methods. The contrast-specific sample availability is summarized in Table 2.

### 3.1. Participant Demographics and Cohort Comparability

Because age and sex may influence balance and postural control measurements, the demographic characteristics were summarized by cohort before interpreting the clustering results. The age distributions were similar between the post-COVID-19 and control groups among participants with valid age information. The post-COVID-19 group had a mean age of 44.33 ± 17.49 years, whereas the control group had a mean age of 45.34 ± 22.06 years. The age distributions did not differ significantly between cohorts when using either a Mann–Whitney U test (p=0.806) or Welch’s *t*-test (p=0.837). Among participants with reported male/female sex, the sex distribution difference between cohorts was not statistically significant when using Fisher’s exact test (p=0.181). Age and sex were used only for cohort characterization and were not included as inputs to the VQ-VAE, BQM/QUBO clustering, GMM clustering, or participant-level control-distant burden scoring procedures. Participant demographics by cohort are summarized in Table 3.

Because the age and sex metadata were incomplete and the control cohort was modest in size, no formal age-stratified, sex-stratified, or leave-one-control-out sensitivity analysis was performed. The demographic analysis was therefore used only as a cohort comparability check, and residual demographic confounding cannot be excluded.

### 3.2. QUBO Optimization Characteristics Across Balance-Derived Contrasts

For each of the 13 balance-derived task contrasts, a separate control-anchored QUBO problem was constructed and solved. The number of binary variables corresponded to the number of participants available for that contrast and ranged from 123 to 132. The resulting kNN graph contained 2686 to 2981 edges across contrasts, which were encoded as quadratic interactions in the BQM. The RBF bandwidth σ varied across contrasts from 2.12 to 3.56, reflecting the contrast-specific latent space geometry after PCA transformation.

Across the 13 QUBO problems, the lowest-energy samples returned by the QPU ranged from −17.13 to 1.70. Because the all-control-like assignment, in which all binary variables are set to $z_{i}=0$, is always feasible under Equation (9) and has energy E=0, the QPU-sampled energy for each contrast was interpreted relative to this all-zero reference energy. Negative QPU-sampled energies indicate that the sampled QPU solution improved upon the all-zero reference under the specified QUBO objective. In contrast, a positive QPU-sampled energy indicates that the sampled solution did not improve upon this reference and should therefore be interpreted cautiously from an optimization standpoint. These values summarize the optimization outcomes for each contrast-specific BQM and should be interpreted as objective-specific energy values rather than directly comparable physiological severity scores across tasks. The contrast-specific QUBO optimization characteristics are summarized in Table 4. Because these BQM formulations define the objective being optimized, the identical contrast-specific BQMs were also solved using simulated annealing and tabu search as classical same-objective solver-level references.

### 3.3. Control-Anchored QUBO Clustering Identifies Selective Control-Distant Balance Profiles

For each balance-derived task contrast, the QPU-sampled QUBO solution was converted into binary control-like and control-distant assignments for downstream assignment summaries. The sampled energies were interpreted relative to the feasible all-control-like reference assignment described in Table 4. Across all 13 contrasts, control-anchored QUBO clustering assigned only 1 of 307 available control participant–contrast assignments (0.3%) to the control-distant group. This isolated control-distant assignment occurred in the $\Delta S_{\mathrm{dual}-\mathrm{single}}$ Romberg (Closed Eyes) contrast. Because this contrast had a positive QPU-sampled energy relative to the all-zero reference, this isolated assignment is interpreted cautiously as part of the sampled QPU assignment pattern rather than as evidence of an improvement over the all-control-like reference. Thus, the QUBO formulation largely preserved the intended control reference structure across balance-derived task contrasts.

Among post-COVID-19 participants, control-distant assignments were more frequent but remained selective. Across individual task contrasts, the proportion of post-COVID-19 participants assigned to the control-distant group ranged from 4.8% to 15.4%. Overall, 118 of 1366 available post-COVID-19 participant–contrast assignments were labeled as control-distant, corresponding to 8.6% of all post-COVID-19 participant–contrast assignments. The highest post-COVID-19 control-distant assignment rate was observed in the $\Delta S_{\mathrm{dual}-\mathrm{single}}$ CTSIB (Open Eyes) contrast, in which 16 of 104 post-COVID-19 participants were classified as control-distant.

These findings indicate that the QUBO objective did not simply separate participants according to COVID-19 status. Instead, it identified a relatively small subset of post-COVID-19 participants with control-distant latent balance profiles while preserving the control-group reference. The positive-energy QPU-sampled solution for the Romberg Closed Eyes cognitive dual-task contrast indicates that this particular contrast was less favorable from an optimization standpoint and is therefore interpreted cautiously. It was nevertheless retained in the downstream sampled assignment summaries for transparency and for consistency with the reported QPU-sampled assignment analysis. This selective pattern is consistent with the heterogeneous nature of post-COVID-19 neuromotor effects, in which control-distant balance patterns may be present in only a subset of post-COVID-19 individuals rather than uniformly across the entire group.

**Figure 3:**
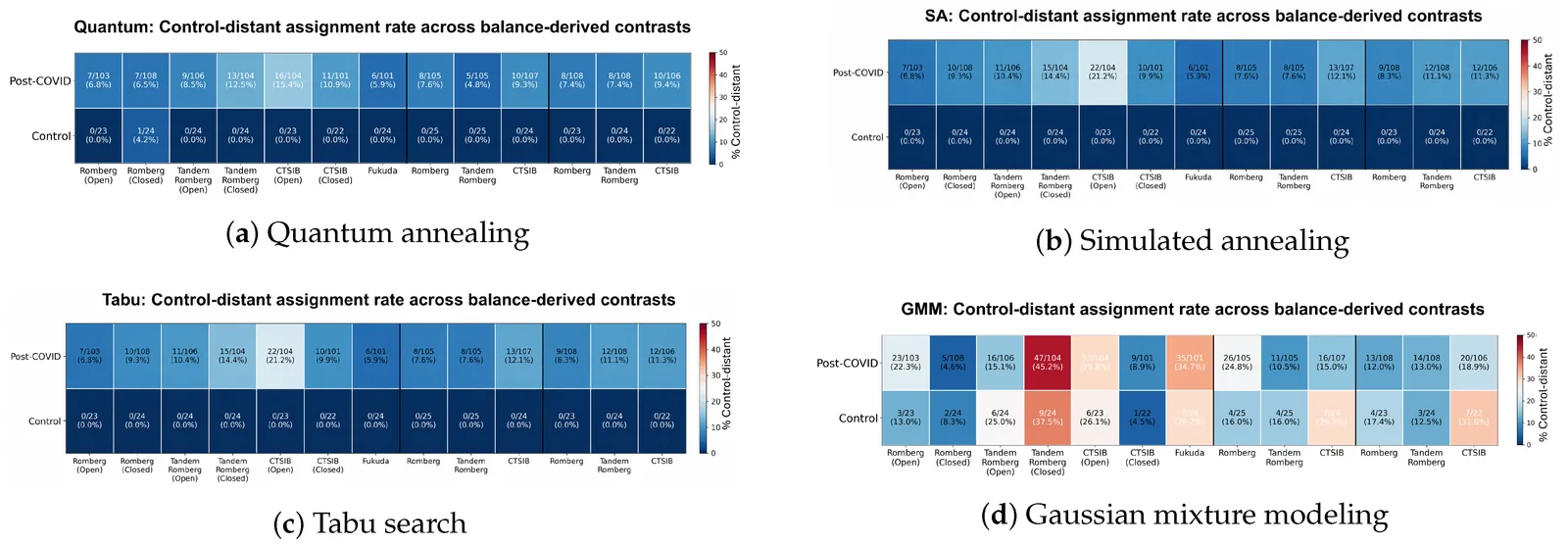
Contrast-level control-distant assignment rates across balance-derived task contrasts. Heatmaps show control-distant assignment rates for post-COVID-19 and control-group participants across the 13 balance-derived task contrasts. Each cell reports the number of control-distant assignments over the number of available participants for that contrast, followed by the corresponding percentage in parentheses. (**a**) shows the quantum annealing-sampled solution for the control-anchored QUBO formulation. (**b**,**c**) show simulated annealing and tabu search applied to the same contrast-specific BQM/QUBO formulations. (**d**) shows the GMM clustering reference. The three BQM-based solvers produced sparse control-distant assignments among post-COVID-19 participants while preserving the control-group reference structure, with simulated annealing and tabu search assigning no control-group participant–contrast samples to the control-distant group. In contrast, GMM assigned a substantially larger proportion of control-group participant–contrast assignments to the control-distant group across multiple contrasts, indicating weaker alignment with the intended control group-preserving objective.

The numerical assignment rates across the BQM-based solvers and the GMM reference analysis are summarized in Table 5, and the contrast-level assignment patterns are visualized in Figure 3. Simulated annealing and tabu search, which were applied to the identical contrast-specific BQM/QUBO formulations, produced contrast-level control-distant assignment patterns that were similar to the quantum annealing-sampled solution but slightly broader among post-COVID-19 participants. Both same-BQM classical solvers assigned 0 of 307 control-group participant–contrast assignments (0.0%) and 143 of 1366 post-COVID-19 participant–contrast assignments (10.5%) to the control-distant group. Compared with GMM, the BQM-based solvers produced markedly lower rates of assigning control-group participants to the control-distant group across task contrasts, consistent with greater alignment with the intended control group-preserving objective.

### 3.4. Participant-Level Control-Distant Burden Across Task Contrasts

The participant-level control-distant burden was computed by aggregating the contrast-specific control-distant assignments across the 13 available balance-derived task contrasts. For each participant, the control-distant count represented the number of contrasts assigned to the control-distant group, and the control-distant burden score represented this count normalized by the number of completed contrasts. This aggregation allowed incomplete task availability to be retained while enabling participant-level ranking.

**Figure 4:**
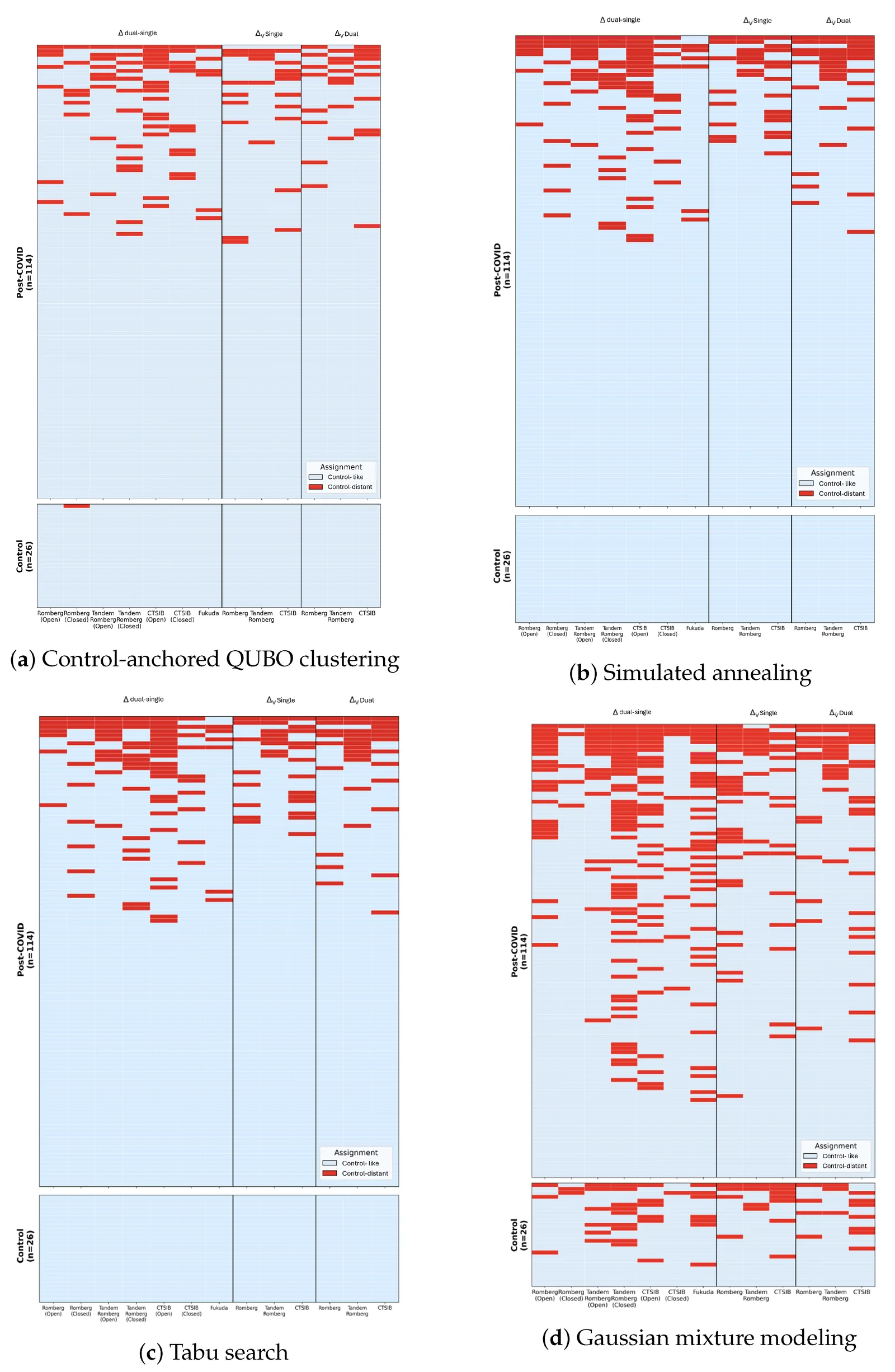
Participant-level control-distant burden across balance-derived task contrasts. Each row represents one participant and each column represents one task contrast. Red cells indicate control-distant assignments, whereas light-blue cells indicate control-like assignments. Participants are grouped by cohort and sorted independently within each method by control-distant burden for visualization. Panel (**a**) shows the quantum annealing-sampled solution for the control-anchored QUBO formulation. Panels (**b**,**c**) show simulated annealing and tabu search applied to the same control-anchored BQM/QUBO formulation. Panel (**d**) shows the GMM analysis as an unconstrained classical clustering reference.

The participant-level control-distant burden heatmaps are shown in Figure 4. Under the quantum annealing solution for the control-anchored QUBO formulation, control-distant assignments were concentrated in a subset of post-COVID-19 participants, while the control group showed a minimal control-distant burden. Most control-group participants had no control-distant assignments across all available contrasts, consistent with the contrast-level preservation of the control-group reference structure.

Simulated annealing and tabu search, which were applied to the same control-anchored BQM/QUBO formulation, showed similar participant-level control-distant burden patterns to the quantum annealing solution. These same-BQM classical solvers retained the same sparse, control-preserving structure but produced slightly broader control-distant assignment patterns among post-COVID-19 participants, consistent with the contrast-level solver comparison. In contrast, GMM produced a broader distribution of control-distant assignments across both post-COVID-19 and control participants.

This broader spread indicates that GMM was more likely to assign control-group participants to control-distant latent components, consistent with the higher control-group control-distant rates observed at the contrast level. Together, these participant-level patterns support the interpretation that the control-anchored BQM/QUBO formulation, whether solved using quantum annealing, simulated annealing, or tabu search, was more aligned with the intended sparse, control-group-preserving objective for identifying control-distant profiles than the unconstrained GMM clustering reference.

### 3.5. Solver-Level, Control Distance Baseline, and GMM Reference Comparisons

To distinguish between solver-level effects, objective-level effects, and unconstrained latent space clustering behavior, we performed three complementary comparisons. First, the quantum annealing-sampled solution was compared with simulated annealing and tabu search applied to the identical contrast-specific BQM/QUBO formulations. This comparison assessed whether the sparse, control-preserving assignment pattern was specific to the quantum annealing sampler or was largely determined by the control-anchored BQM/QUBO objective. Second, the QUBO results were compared with a simple control distance threshold baseline that used the same control distance score as the QUBO objective but omitted the sparsity, outlier reward, graph smoothness, and BQM optimization terms. This comparison assessed whether the full QUBO objective produced assignment patterns beyond those obtained by thresholding the latent space distance from the control-group reference alone. Third, the QUBO results were compared with a two-component Gaussian mixture model (GMM) fitted independently to each task contrast in the 128-dimensional VQ-VAE latent space. This comparison assessed whether similar control-distant assignments would arise from an unconstrained geometry-driven latent space clustering reference.

The same-BQM solver comparison showed similar aggregate behavior across quantum annealing, simulated annealing, and tabu search. Simulated annealing and tabu search both assigned 0 of 307 control-group participant–contrast assignments (0.0%) and 143 of 1366 post-COVID-19 participant–contrast assignments (10.5%) to the control-distant group. Thus, relative to the quantum annealing-sampled solution, the classical same-BQM solvers produced similar but slightly broader control-distant post-COVID-19 subsets while assigning no control-group participant–contrast samples to the control-distant group. Agreement between solvers was quantified using the Jaccard index for the control-distant assignment sets. The Jaccard index was 0.634 for quantum annealing versus simulated annealing and 0.634 for quantum annealing versus tabu search, whereas simulated annealing and tabu search produced identical control-distant assignment sets, with a Jaccard index of 1.000. These results indicate that the sparse, control-preserving assignment structure was largely driven by the BQM/QUBO formulation, although exact membership of the smaller control-distant subset remained solver-dependent.

Because all three BQM-based solvers optimized the same contrast-specific objective, the achieved BQM energy was also used as a direct solver-level solution quality measure. The best energies obtained by quantum annealing, simulated annealing, and tabu search for each task contrast are reported in Supplementary Table S3.

No controlled end-to-end runtime comparison was performed between quantum annealing, simulated annealing, and tabu search. Runtime was not used as a performance outcome because the present study focused on the control-anchored BQM/QUBO formulation and resulting assignment structure rather than the computational speed. The QPU wall clock time includes preprocessing, BQM construction, embedding, network transfer, queueing, QPU sampling, sample return, and postprocessing, which were not prospectively logged under matched benchmarking conditions. Therefore, the runtime was not interpreted as evidence for a quantum advantage or solver superiority in this study.

The control distance threshold baseline produced broader control-distant assignments than the full QUBO formulation, particularly among control-group participants. Using the 95th percentile of the control distance scores among control-group participants as a contrast-specific threshold, this baseline assigned 26 of 307 control-group participant–contrast assignments (8.5%) and 179 of 1366 post-COVID-19 participant–contrast assignments (13.1%) to the control-distant group. In comparison, the control-anchored QUBO formulation assigned 1 of 307 control-group participant–contrast assignments (0.3%) and 118 of 1366 post-COVID-19 participant–contrast assignments (8.6%) to the control-distant group. The threshold baseline showed high overall agreement with the quantum annealing-sampled assignments (93.7%) but only moderate overlap for the control-distant subsets, with an overall Jaccard index of 0.512. These findings indicate that the full QUBO objective was not equivalent to simple control distance thresholding; instead, the control anchoring, sparsity, and graph smoothness terms produced a more selective and control-preserving assignment pattern. The contrast-level threshold baseline results are reported in Supplementary Table S2.

In contrast, GMM produced substantially higher control-distant assignment rates among control-group participants than the QUBO-based method. Across the 13 task contrasts, the control-group control-distant rate under GMM ranged from 4.5% to 37.5%, compared with 0.0–4.2% under control-anchored QUBO clustering. Aggregated across all control-group participant–contrast assignments, GMM assigned 63 of 307 control-group participant–contrast assignments to the control-distant group, corresponding to 20.5%. In contrast, control-anchored QUBO clustering assigned only 1 of 307 control-group participant–contrast assignments (0.3%) to the control-distant group.

GMM also showed highly variable control-distant assignment rates among post-COVID-19 participants, ranging from 4.6% to 45.2% across task contrasts. The highest control-distant rates occurred in the Tandem Romberg Closed Eyes cognitive dual-task, Fukuda, the CTSIB Open Eyes cognitive dual-task, and single-task visual condition Romberg contrasts. However, because several of these contrasts also showed high control-group control-distant rates, these GMM assignments appear to reflect broad latent space separation rather than the selective identification of a control-preserving post-COVID-19 control-distant balance profile.

This pattern was also evident at the participant level. Under GMM, only 5 of 26 control-group participants were classified as control-like across all available task contrasts. Moreover, 11 of 26 control-group participants were assigned to the probable or strong control-distant burden groups, compared with 35 of 114 post-COVID-19 participants. The mean number of control-distant task contrasts was similar between control-group and post-COVID-19 participants under GMM, with 2.42 control-distant contrasts in control-group participants and 2.33 in post-COVID-19 participants. These findings indicate that GMM captured broad latent space heterogeneity, but its assignments were less aligned with the specific control-preserving and sparsity-constrained objective of the present study. This comparison should therefore be interpreted as an objective alignment comparison rather than evidence that one clustering method is generally superior to another.

The GMM AIC and BIC values were retained as descriptive model fit criteria for the fitted two-component mixture models, but these criteria do not by themselves establish clustering quality or alignment with the control-preserving phenotyping objective. This is consistent with the role of GMM as a geometry-driven latent space clustering method: it can partition the latent distribution into Gaussian components, but it does not explicitly encode control anchoring, the sparsity of control-distant assignments, or graph smoothness. In contrast, the BQM/QUBO objective incorporated these assumptions directly into the optimization problem, resulting in assignments that were more aligned with the intended control-preserving and sparsity-constrained objective. This objective alignment should not be interpreted as general clustering superiority, because GMM and the BQM/QUBO formulation optimize fundamentally different objectives. The same-BQM solver and GMM comparisons are summarized in Table 5, and the control distance threshold baseline is summarized in Supplementary Table S2.

### 3.6. VQ-VAE Representation Quality Assessment

Because downstream clustering depended on the learned VQ-VAE latent representation, we performed a post hoc assessment of reconstruction quality and codebook usage. The VQ-VAE was trained on 1673 participant–contrast samples with input shape (1673,1,128,32), latent dimension 64, codebook size 128, and commitment weight β=0.25.

Across all participant–contrast samples, the trained VQ-VAE showed low reconstruction errors, with a mean reconstruction MSE of $8.13\times 10^{-4}$, a median MSE of $1.93\times 10^{-4}$, and a standard deviation of $2.49\times 10^{-3}$. The reconstruction MSE values ranged from $3.0\times 10^{-6}$ to $4.73\times 10^{-2}$. When summarized by task contrast, the mean reconstruction MSE ranged from $4.79\times 10^{-4}$ to $1.22\times 10^{-3}$, indicating that the reconstruction quality was broadly consistent across balance-derived contrasts.

Codebook usage was sparse but did not indicate complete codebook collapse. Of the 128 available codebook entries, 22 were used across the encoded participant–contrast samples, corresponding to a utilization rate of 17.2%. The codebook perplexity was 5.25, indicating that the learned representation relied on a limited subset of discrete latent codes. This sparse codebook usage is retained and reported because it may influence downstream clustering behavior.

Overall, the VQ-VAE representation showed low reconstruction errors and nonzero codebook utilization, supporting its use as a compact latent representation for subsequent BQM/QUBO clustering and GMM reference analysis. However, because codebook usage was sparse, the derived control-distant assignments should be interpreted as dependent on the learned discrete representation. This motivated the post hoc VQ-VAE seed stability analysis reported below. Broader VQ-VAE hyperparameter sensitivity, including the latent dimension, codebook size, commitment weight, and training duration, remains an important direction for future work.

### 3.7. VQ-VAE Seed Stability and Downstream Assignment Robustness

Because downstream clustering depends on the learned discrete VQ-VAE representation, we performed a post hoc seed stability analysis using three VQ-VAE random initializations with the same architecture and hyperparameters. Reconstruction errors remained consistently low across seeds, with mean reconstruction MSE values of $7.73\times 10^{-4}$, $8.36\times 10^{-4}$, and $1.15\times 10^{-3}$. No seed showed complete codebook collapse. However, codebook usage varied across initializations, with 28, 49, and 34 of 128 codewords used and codebook perplexity values of 2.93, 5.69, and 3.80, respectively. These results indicate that reconstruction errors were consistently low across seeds, whereas the learned discrete codebook usage was partly initialization-dependent.

To assess whether this representation-level variability affected downstream subgroup discovery, each seed-derived latent representation was passed through the same control-anchored BQM/QUBO construction and evaluated using simulated annealing as a matched same-BQM reference. Across the three VQ-VAE seeds, simulated annealing assigned 0 of 307 control-group participant–contrast samples to the control-distant group for every seed, indicating stable preservation of the control-group reference structure. Post-COVID-19 control-distant assignments varied from 90 to 172 of 1366 participant–contrast samples, corresponding to 6.6–12.6%. Pairwise overall assignment agreement between seeds was high, ranging from 94.4% to 96.0%, whereas the control-distant Jaccard overlap ranged from 0.472 to 0.624. Thus, the overall sparse, control-preserving assignment structure was stable across VQ-VAE seeds, but the exact membership of the smaller control-distant subset remained partly dependent on the learned discrete representation.

A parallel QPU-sampled analysis of the same three seed-derived latent representations produced nearly identical qualitative results. Across the three seeds, the QPU-sampled workflow also assigned 0 of 307 control-group participant–contrast samples to the control-distant group for every seed and assigned 91–173 of 1366 post-COVID-19 participant–contrast samples to the control-distant group. Pairwise overall assignment agreement ranged from 94.3% to 96.0%, and the control-distant Jaccard overlap ranged from 0.467 to 0.626. These findings confirm that the VQ-VAE seed stability pattern was not specific to the simulated annealing reference solver.

### 3.8. Parameter Sensitivity and Assignment Stability

To assess the robustness of the control-anchored BQM/QUBO formulation, we performed parameter sensitivity analyses by perturbing the QUBO-related settings and comparing the resulting control-distant assignment patterns with the baseline solutions. Stability was quantified using two complementary measures: overall binary assignment agreement across all participant–contrast samples and the Jaccard index computed on the control-distant assignment set. Overall agreement summarizes stability across both control-like and control-distant assignments, whereas the Jaccard index focuses specifically on the smaller control-distant subset. Because control-like assignments represented the majority of participant–contrast samples, the overall agreement should be interpreted together with the control-distant Jaccard index rather than as a standalone measure of subgroup stability. The tested parameter values and corresponding agreement metrics are reported in Supplementary Table S4.

For simulated annealing, the assignment patterns showed high overall agreement and relatively stable control-distant subset membership across the tested parameter perturbations. The overall binary assignment agreement relative to the baseline simulated annealing solution ranged from 98.3% to 100.0%, and the Jaccard index for the control-distant assignment set ranged from 0.836 to 1.000. These results indicate that, under the classical same-BQM solver setting, the overall assignment structure and the membership of the control-distant subset were both comparatively stable across the tested parameter range.

A representative QPU sensitivity analysis showed high overall assignment agreement but greater variability in the exact membership of the control-distant subset. The overall binary assignment agreement relative to the representative QPU baseline ranged from 93.6% to 100.0%, whereas the Jaccard index for the control-distant assignment set ranged from 0.443 to 1.000. Thus, most participant–contrast assignments remained unchanged across QPU parameter perturbations, but the exact composition of the smaller control-distant subset was more sensitive than in the simulated annealing analysis.

Together, these sensitivity analyses suggest that the control-anchored BQM/QUBO formulation produced stable overall assignment patterns, while the precise membership of the sparse control-distant subset remained partly dependent on the solver behavior and parameter settings. These findings support the use of the control-distant burden as an exploratory ranking measure but also indicate that individual case-level assignments should be interpreted cautiously, particularly when based on only one or two control-distant task contrasts, as will be detailed in the following sections.

### 3.9. Exploratory Clinical Context Review of QUBO, Same-BQM Solver, and GMM Rankings

To assess the clinical context of the participant-level rankings, we performed an exploratory post hoc review of the available clinical notes for selected participants. Clinical notes, demographic variables, and diagnostic annotations were not used as inputs to the VQ-VAE, quantum annealing, simulated annealing, tabu search, GMM clustering, or participant-level control-distant burden scoring. Therefore, this analysis should be interpreted strictly as a qualitative and anecdotal clinical context review, not as clinical validation and not as an assessment of diagnostic sensitivity, specificity, or case detection performance.

Among participants with available clinical notes, PC013 represented the clearest supportive case. This participant was the only individual in the cohort with a physician-documented long COVID evaluation note in the available records. The quantum annealing-sampled QUBO solution assigned this participant to the probable control-distant group, with control-distant assignments in 4 of 10 available task contrasts. Simulated annealing and tabu search produced the same participant-level burden for this case, with control-distant assignments in 4 of 10 available task contrasts. GMM also assigned this participant to the probable control-distant group, with control-distant assignments in 5 of 10 available task contrasts. Although the exact ranks and contrast counts differed across methods, all four methods identified this participant as having an elevated control-distant burden across the available balance-derived contrasts. This concordance provides limited case-level clinical context consistency for the latent balance phenotype identified in this participant, but it remains anecdotal because only one participant had a physician-documented long COVID evaluation note and clinical notes were incomplete across participants and were not used as model inputs.

However, the GMM rankings showed a different pattern from the BQM-based rankings. GMM assigned a high control-distant burden to several control-group participants. A notable example was NC008, a control-group participant who was ranked first by GMM and assigned to the control-distant group in all seven of seven available task contrasts. In contrast, the quantum annealing-sampled QUBO solution classified the same participant as having only a single-test deviation, with control-distant assignment in one of seven available contrasts. Simulated annealing and tabu search classified this participant as control-like, with zero of seven control-distant assignments. This participant corresponded to the isolated control-group participant with a QUBO-derived control-distant assignment, indicating that the QUBO objective did not force all control-group participants to remain control-like when an individual contrast showed a deviant latent profile. Because this participant had fewer available task contrasts than many other participants, the GMM rank should not be interpreted as an absolute clinical severity ranking. Nevertheless, this case illustrates that GMM was more likely to produce a high control-distant burden in control-group participants, whereas the BQM-based methods produced more conservative control-distant assignment patterns relative to the control-anchored objective.

A similar pattern was observed in other control-group participants. Several control-group participants, including NC002, NC003, NC004, and NC005, were assigned a possible or probable control-distant burden by GMM despite the available notes indicating no known COVID-19 history or a negative antibody status in some cases. In contrast, the quantum annealing-sampled QUBO solution classified these control-group participants as control-like across their available task contrasts. Simulated annealing and tabu search similarly preserved the control-group reference structure at the aggregate level, assigning no control-group participant–contrast samples to the control-distant group across the 13 task contrasts. These selected examples are consistent with the contrast-level and participant-level analyses showing weaker control-group preservation under GMM compared with the control-anchored BQM/QUBO formulation.

Clinical notes related to non-COVID-19 conditions or testing artifacts also did not necessarily correspond to a high BQM-based control-distant burden. For example, PC080 had notes indicating chronic fatigue or long-hauler symptoms, an autoimmune history, and cardiovascular issues, but the quantum annealing-sampled QUBO solution, simulated annealing, and tabu search all classified this participant as having only a single-test deviation, with 1 of 12 available task contrasts assigned to the control-distant group. GMM, in contrast, classified this participant as probable control-distant, with 3 of 12 available contrasts assigned to the control-distant group. Similarly, NC018 had notes indicating discomfort during balance testing and wall holding behavior, yet the quantum annealing-sampled QUBO solution, simulated annealing, and tabu search all classified this participant as control-like, whereas GMM classified the participant as probable control-distant. This control-group case was retained in the clinical context review because it represented a non-COVID-19 participant with an available note suggesting atypical testing behavior, allowing an inspection of whether the model-derived control-distant burden reflected clinical status, task performance artifacts, or broader latent space variability. These cases suggest that GMM may be more sensitive to broad latent space deviations, including deviations that may reflect non-COVID-19 variability or testing artifacts, whereas the BQM-based methods produced more selective control-distant assignments relative to the control-anchored objective.

Selected clinical note examples are summarized in Table 6. Overall, this exploratory review does not establish that control-anchored QUBO clustering can diagnose long COVID, identify clinically affected participants, or estimate clinical sensitivity or specificity. Rather, it provides a qualitative clinical context description of selected cases. The participant with the only physician-documented long COVID evaluation note was assigned to a control-distant subgroup across all BQM-based solvers, while most control-group participants were preserved as control-like. Importantly, the isolated quantum annealing-sampled QUBO control-distant assignment among control-group participants was retained and reviewed rather than discarded, supporting the interpretation that the control-anchored objective was conservative but not absolute. In contrast, GMM produced broader control-distant assignments and included more control-group participants among high-burden cases, suggesting that GMM captured general latent heterogeneity but was less aligned with the intended control group-preserving post-COVID-19 control-distant phenotyping objective.

### 3.10. Frequency Band Interpretation of Control-Distant Balance Profiles

#### 3.10.1. Band-Averaged Physiological Profiles

To interpret the control-distant balance profiles in physiologically meaningful terms, we summarized the joint-wise spectral contrast maps as four predefined frequency bands. The full joint-resolved heatmaps contained 17 joint pairs and were useful for detailed inspection, but they were difficult to compare across contrasts and clustering methods. Therefore, for the main frequency band interpretation, ΔEnergy values were averaged across the 17 joint pairs within each frequency band. This produced compact band-level profiles for each task contrast, participant subgroup, and clustering method.

The four frequency bands were interpreted as postural sway-dominant activity, mid–low-frequency postural control activity, corrective or overcompensatory activity, and tremor-like/high-frequency components. In the present analysis, the interpretation focused primarily on Band 1 and Band 2, because these low-frequency and mid–low-frequency components are most directly related to postural sway and balance control dynamics. Band 3 and Band 4 were retained for visualization and exploratory interpretation but were interpreted more cautiously because they may reflect compensatory movement, high-frequency correction, tremor-like activity, measurement noise, or task-specific movement artifacts.

**Figure 5:**
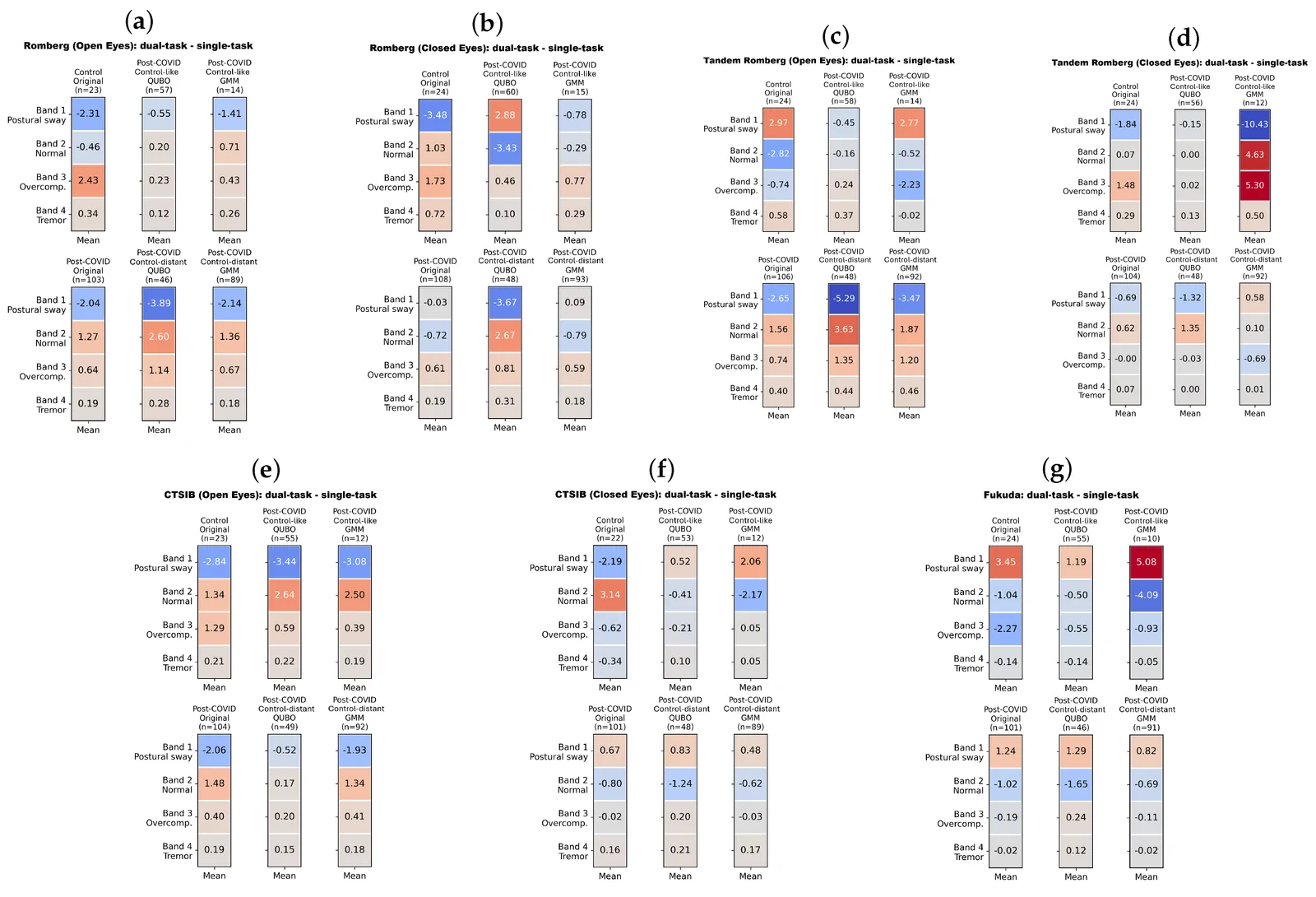
Frequency band profiles for cognitive dual-task versus single-task balance contrasts. Band-averaged ΔEnergy profiles are shown for each task, with $\Delta S_{\mathrm{dual}-\mathrm{single}}$ defined as cognitive dual-task minus single-task. Values represent mean ΔEnergy across 17 joint pairs within each frequency band. Columns show original group profiles and QUBO- and GMM-derived post-COVID-19 control-like and control-distant profiles. Panels show (**a**) Romberg Open Eyes, (**b**) Romberg Closed Eyes, (**c**) Tandem Romberg Open Eyes, (**d**) Tandem Romberg Closed Eyes, (**e**) CTSIB Open Eyes, (**f**) CTSIB Closed Eyes, and (**g**) Fukuda. Subgroup sample sizes and bootstrap 95% confidence intervals for the corresponding band-averaged mean ΔEnergy values are reported in Supplementary Table S1.

**Figure 6:**
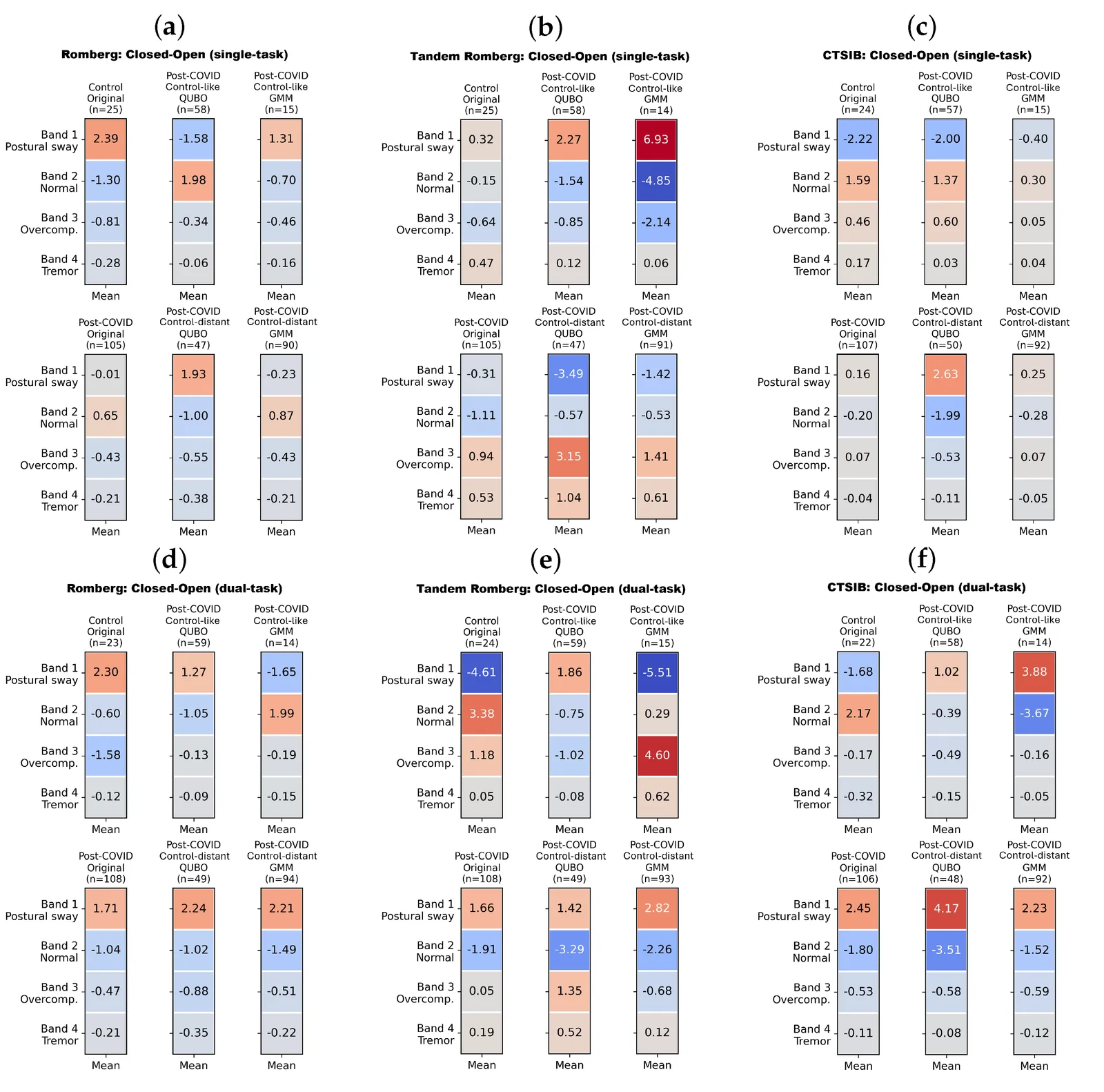
Frequency band profiles for visual removal balance contrasts. Band-averaged ΔEnergy profiles are shown for closed-eyes minus open-eyes contrasts. Values represent mean ΔEnergy across 17 joint pairs within each frequency band. Columns show original group profiles and QUBO- and GMM-derived post-COVID-19 control-like and control-distant profiles. The top row shows single-task visual condition contrasts and the bottom row shows cognitive dual-task visual condition contrasts: (**a**,**d**) Romberg, (**b**,**e**) Tandem Romberg, and (**c**,**f**) CTSIB. Subgroup sample sizes and bootstrap 95% confidence intervals for the corresponding band-averaged mean ΔEnergy values are reported in Supplementary Table S1.

Figure 5 and Figure 6 show the compact frequency band profiles for the cognitive dual-task versus single-task contrasts and the visual removal contrasts, respectively. In each panel, the original control-group and post-COVID-19 group profiles are shown alongside post-COVID-19 control-like and control-distant profiles derived from control-anchored QUBO clustering and GMM. This layout allows a direct comparison between the original cohort grouping and the latent subgroups identified by each clustering approach.

Because the control-like and control-distant subgroups varied in size across task contrasts and clustering methods, the subgroup sample sizes and bootstrap 95% confidence intervals for the band-averaged ΔEnergy profiles are reported in Supplementary Table S1. The mean profiles in Figure 5 and Figure 6 should therefore be interpreted together with these variability summaries, particularly for contrasts and methods in which one of the derived subgroups contained relatively few participants. For readability, confidence intervals are not overlaid directly on the figures because they already summarize multiple tasks, subgroups, methods, and frequency bands.

#### 3.10.2. Band-Specific Patterns in QUBO and GMM Subgroups

Across the cognitive dual-task versus single-task contrasts, QUBO-derived control-distant profiles frequently showed stronger band-specific separation than the corresponding GMM profiles. In several contrasts, including Romberg Open Eyes, Tandem Romberg Open Eyes, CTSIB Closed Eyes, and Fukuda, the QUBO control-distant subgroup showed more prominent changes in Band 1 and Band 2 compared with the QUBO control-like subgroup and the post-COVID-19 original group. This suggests that the QUBO control-distant assignments captured a subset of post-COVID-19 participants with more distinct low-frequency balance control responses that were partially averaged out when all post-COVID-19 participants were analyzed together.

In Romberg Open Eyes, for example, the QUBO control-distant profile showed a stronger decrease in Band 1 and a stronger increase in Band 2 relative to the post-COVID-19 original group, while preserving the overall direction of the post-COVID-19 response. Similar Band 1/Band 2 separation was observed in Tandem Romberg Open Eyes and Fukuda. These patterns suggest that, for several cognitive dual-task-related contrasts, QUBO selected control-distant subgroups whose frequency band responses were not simply arbitrary partitions of the latent space but reflected interpretable changes in postural control dynamics.

Romberg Closed Eyes showed a different pattern. In this contrast, the QUBO control-like and control-distant profiles showed Band 1/Band 2 directions that were less consistent with the post-COVID-19 original profile than in other cognitive dual-task-related contrasts. This made the physiological interpretation of the QUBO partition less straightforward for this task. Notably, Romberg Closed Eyes was also the only contrast in which a control-group participant was assigned to the control-distant group by control-anchored QUBO clustering, as shown in Figure 4. This suggests that the Romberg Closed Eyes cognitive dual-task contrast may represent a more difficult latent separation problem than several other cognitive dual-task contrasts.

In CTSIB Open Eyes, the QUBO control-distant profile showed attenuation rather than amplification of the original post-COVID-19 band pattern. This indicates that control-distant profiles do not necessarily correspond to increased ΔEnergy in all tasks. Instead, control-distant balance phenotypes may involve either the amplification, attenuation, or inversion of specific frequency band responses depending on the sensory and cognitive context of the task.

The visual removal contrasts showed additional task-dependent patterns. In the single-task Romberg and single-task CTSIB visual condition contrasts, the QUBO control-distant profiles showed increased Band 1 and decreased Band 2 relative to the post-COVID-19 original group, suggesting altered low-frequency sway and postural control responses when visual input was removed. In the single-task Tandem Romberg visual condition contrast, however, the QUBO control-distant profile showed a stronger Band 3 component, suggesting that the more challenging tandem stance may induce compensatory spectral responses rather than a simple increase in low-frequency sway.

Under cognitive dual-task visual removal conditions, the CTSIB contrast showed a particularly strong QUBO control-distant pattern, with increased Band 1 and decreased Band 2 relative to the post-COVID-19 original group. This pattern suggests that the combination of visual removal and cognitive load may reveal control-distant balance control responses in a subset of post-COVID-19 participants. However, because this contrast also had a less favorable QUBO energy than several other contrasts, the physiological interpretation should remain exploratory.

Compared with QUBO, GMM profiles were generally less selective. In several contrasts, the GMM control-distant profiles resembled the post-COVID-19 original group more closely and showed weaker separation from the GMM control-like profiles. In other cases, GMM produced large band-specific changes in the control-like group rather than in the control-distant group or generated profiles that appeared broadly blended across subgroups. This is consistent with the earlier observation that GMM captured latent space heterogeneity but showed weaker control-group preservation and less selective control-distant assignment than the QUBO formulation.

#### 3.10.3. Relationship Between QPU-Sampled Energy and Band Profile Interpretability

The frequency band profiles also suggested a relationship between the QUBO optimization behavior and downstream interpretability. As summarized in Table 4, each task contrast was solved as a separate QUBO problem, yielding a contrast-specific QPU-sampled energy. This sampled energy reflects the degree to which the sampled binary assignment satisfied the combined control-anchoring, sparsity, outlier reward, and graph smoothness terms in the QUBO objective.

Contrasts with less favorable QPU-sampled energy values tended to produce band profiles that were more difficult to interpret physiologically. The clearest example was the Romberg Closed Eyes cognitive dual-task contrast, which had a positive QPU-sampled energy and therefore did not improve upon the all-zero reference. This contrast also showed less consistent Band 1/Band 2 separation in the QUBO frequency band profiles. Similarly, the single-task Tandem Romberg visual condition contrast had a QPU-sampled energy close to zero and showed a more complex control-distant profile, with changes extending beyond Band 1 and Band 2 into Band 3.

In contrast, several lower QPU-sampled-energy solutions produced more interpretable control-distant band profiles. In these contrasts, the QUBO control-distant subgroup often showed Band 1/Band 2 patterns that were directionally related to the post-COVID-19 original profile but more strongly separated from the QUBO control-like subgroup. This suggests that lower-energy sampled solutions may correspond to binary partitions that more closely satisfy the intended control-anchored control-distant objective and yield more physiologically interpretable patterns.

Importantly, the QPU-sampled energy was not interpreted as a physiological severity score or clinical validation metric. Energy values depend on the contrast-specific sample size, graph structure, RBF affinity weights, penalty terms, and overall objective landscape. Therefore, QPU-sampled energy values should not be compared directly as measures of physiological severity across tasks. Instead, the present post hoc analysis suggests that the QPU-sampled energy may serve as an optimization-level indicator of how well a sampled contrast-specific control-distant partition satisfies the intended objective and how suitable the sampled partition may be for downstream physiological interpretation.

Overall, the frequency band analysis supports two complementary findings. First, QUBO-derived control-distant profiles often showed interpretable band-specific postural control patterns, particularly in Band 1 and Band 2, whereas GMM profiles were more blended and less selective. Second, the contrast-specific QPU-sampled energy provided a useful context for assessing the physiological interpretability of the derived band profiles. These findings should be considered exploratory and hypothesis-generating, as no independent clinical ground truth was available to externally validate specific frequency band balance phenotypes.

## 4. Discussion

This study demonstrates the feasibility of a control-anchored, QUBO-based framework for identifying control-distant balance phenotypes in a heterogeneous post-COVID-19 cohort using frequency-domain motion representations and label-free latent representation learning followed by objective-driven clustering. The main finding is not that post-COVID-19 status can be directly detected from balance data but that a subset of post-COVID-19 participants exhibited control-distant latent balance profiles while the control-group reference structure was largely preserved. This distinction is important because post-COVID-19 status represents the infection history rather than a ground truth for persistent neuromotor impairment. The observation that most post-COVID-19 participants were assigned to control-like profiles is therefore not a failure of the model but is consistent with the expected heterogeneity of post-acute sequelae, where balance-related dysfunction may be present only in a subset of individuals.

A key contribution of this work is the use of an objective-driven BQM/QUBO formulation rather than a purely geometry-driven clustering model. The QUBO objective explicitly combined control-group anchoring, the sparsity of control-distant assignments, control distance reward among post-COVID-19 participants, and graph smoothness over the latent kNN structure. This design encouraged control-distant assignments only when they were both distant from the control-group reference distribution and locally supported by the latent graph, while penalizing control-distant assignment among control-group participants. Simulated annealing and tabu search applied to the same BQM/QUBO formulation provided classical same-objective solver-level references for the control-anchored objective. These same-BQM solvers produced aggregate assignment patterns that were similar to the quantum annealing solution, supporting the interpretation that the sparse, control group-preserving structure was driven primarily by the BQM/QUBO objective rather than by a single sampler alone. However, the exact membership of the smaller control-distant subset remained partly solver-dependent, as reflected by the solver-level agreement and sensitivity analyses. Thus, the primary methodological contribution of this study is the control-anchored BQM/QUBO objective for sparse control-distant subgroup discovery, rather than a claim about the solver-specific superiority of quantum annealing.

In contrast, GMM provided a useful unconstrained classical clustering reference but addressed a different clustering question. GMM partitioned the latent feature distribution into Gaussian components without explicitly encoding control-group preservation, sparsity, or graph consistency. Thus, GMM appeared sensitive to latent space heterogeneity but not necessarily specific to a control group-preserving control-distant post-COVID-19 balance phenotype. This difference likely explains why GMM produced broader control-distant assignments and greater control-group involvement, whereas the BQM-based methods produced more selective control-distant profiles aligned with the intended control-anchored objective. This does not imply that GMM failed as a clustering method or that the BQM/QUBO approach is generally superior for clustering. Rather, it indicates objective mismatch: GMM optimized an unconstrained mixture-model likelihood, whereas the BQM/QUBO formulation explicitly optimized a control-preserving and sparsity-constrained control-distant phenotyping objective. Importantly, this control group preservation should not be interpreted as independent clinical validation, because it is partly expected from the control-anchored objective design. Evidence for clinical relevance must therefore come from complementary analyses, including frequency band interpretability, participant-level burden patterns, and exploratory clinical context review.

Post-COVID-19-only GMM clustering was not performed in the present study because the primary comparison required a control-group reference for interpreting clusters as control-like versus control-distant. Although post-COVID-19-only clustering may be useful for future subtype discovery within the post-COVID-19 cohort, it would address a different objective from the present control-anchored phenotyping framework.

The frequency band analysis provided an explainable view of what the clustering models appeared to separate. By averaging the joint-wise spectral contrast maps across 17 joint pairs within predefined frequency bands, we obtained compact physiological profiles for each task contrast and subgroup. These profiles suggested that QUBO-derived control-distant assignments often corresponded to interpretable changes in Band 1 and Band 2, which reflect low-frequency postural sway-dominant activity and mid–low-frequency postural control activity. In several contrasts, the QUBO control-distant subgroup showed stronger or more directionally coherent Band 1/Band 2 separation than the post-COVID-19 original group, suggesting that group-level averaging may obscure subgroup-specific balance control responses. In this sense, the band-level analysis functioned as an explanatory layer for the latent clustering output: it did not prove a clinical mechanism, but it helped to translate abstract latent assignments into interpretable balance response patterns. However, this band-level interpretation should not be viewed as fully independent post hoc validation, because the QUBO objective already included an outlier reward term that favored post-COVID-19 samples located farther from the control-group reference distribution in the latent space. The frequency band analysis was therefore used as an explanatory and physiological context layer, not as an independent validation endpoint. Importantly, the predefined frequency band summaries were not used as inputs to the VQ-VAE, QUBO, simulated annealing, tabu search, or GMM clustering procedures. GMM profiles, in comparison, were often more blended or showed stronger responses in control-like groups, consistent with the broader and less selective control-distant assignment behavior observed in the clustering results.

The QPU-sampled energy values provided an additional optimization-level context for interpreting contrast-specific sampled solutions. The QPU-sampled energy was not treated as a physiological severity score or clinical validation metric, because it depends on the sample size, graph structure, RBF affinity weights, penalty terms, and contrast-specific objective landscape. Nevertheless, post hoc inspection suggested that contrasts with less favorable QPU-sampled energy values tended to yield band profiles that were more difficult to interpret physiologically. For example, the Romberg Closed Eyes cognitive dual-task contrast had a positive QPU-sampled energy, indicating that the sampled solution did not improve upon the all-zero reference. This contrast also showed less consistent Band 1/Band 2 separation and was the only contrast in which a control-group participant was assigned to the control-distant group by the main quantum annealing-sampled solution. Similarly, the single-task Tandem Romberg closed-eyes versus open-eyes contrast had a QPU-sampled energy close to zero and produced a more complex control-distant band profile. These observations suggest that the QPU-sampled energy may serve as an optimization-level indicator of how well a sampled contrast-specific binary assignment satisfies the intended control-anchoring, sparsity, outlier reward, and graph smoothness objective. This interpretation remains exploratory, but it provides a potentially useful bridge between the mathematical optimization output and downstream physiological interpretability.

Several limitations should be considered. First, the cohort size was modest, particularly for the control group, and only one participant had a physician-documented long COVID evaluation note in the available records. Although age and sex did not differ significantly between the post-COVID-19 and control groups in the available demographic analysis, residual confounding cannot be excluded because of the smaller control sample size and incomplete metadata. In addition, although most control participants had documented negative COVID-19 testing at enrollment, four control participants reported no known prior COVID-19 history but did not have prior diagnostic confirmation available; this unconfirmed control status uncertainty should be considered when interpreting the control anchor set. The modest control sample size and incomplete demographic metadata also limited the feasibility of formal age-stratified, sex-stratified, missing data, or leave-one-control-out influential sample analyses. Although the available cohort comparability analysis did not show statistically significant age or sex distribution differences between the post-COVID-19 and control groups, age information was incomplete and both cohorts spanned wide age ranges, with fewer participants in the control group. Further stratification would therefore have produced underpowered and unstable subgroup analyses. Consequently, the control anchor should be interpreted as an available reference cohort rather than a fully population-representative normative sample. Future studies with larger and more completely characterized control cohorts should explicitly evaluate age- and sex-stratified stability, missing data effects, and influential control sample sensitivity. Therefore, the clinical review should be interpreted as an exploratory clinical context assessment rather than diagnostic validation. Second, COVID-19 status was used only as a descriptive context and should not be interpreted as a ground truth for persistent neuromotor impairment. Third, clinical notes were incomplete and heterogeneous, and standardized symptom scales, vestibular assessments, autonomic measures, fatigue scores, and longitudinal outcome data were not available for all participants.

Fourth, the balance tasks were performed in a fixed order, with single-task conditions collected before the corresponding cognitive dual-task conditions. Therefore, the cognitive dual-task versus single-task contrasts may reflect not only concurrent cognitive task demands but also repeated-task, learning, habituation, fatigue, or other order-related effects. This limitation is particularly important for interpreting these contrasts as cognitive dual-task effects, and future studies should use randomized or counterbalanced task ordering to better separate concurrent cognitive task demands from repeated-exposure effects. Because these same task contrasts were also used to construct the VQ-VAE latent representations and downstream BQM/QUBO assignments, order-related effects could have contributed to the observed subgroup structure. Therefore, control-distant assignments involving cognitive dual-task contrasts should be interpreted as reflecting task condition-dependent balance response differences rather than isolated cognitive load effects alone. In addition, under the cognitive dual-task condition, the eyes-open trials used Stroop color naming whereas the eyes-closed trials used auditory verbal prompts. Therefore, the cognitive dual-task closed-eyes versus open-eyes contrasts changed both the visual condition and concurrent cognitive task format, and they should not be interpreted as isolating visual input removal alone.

Fifth, the QUBO weights were fixed across all task contrasts. Although this avoided contrast-specific tuning after observing the results, it also means that the same Lagrange parameters were applied to latent graphs with different sample sizes, affinity structures, control distance distributions, and optimization landscapes. Sixth, PCA reduction and kNN graph sparsification were used as hardware-aware preprocessing steps to obtain compact QPU-compatible BQM formulations. Although PCA preserved 95% of the explained latent variance and kNN sparsification preserved the local graph structure, these steps may discard lower-variance latent directions or longer-range graph relationships that could be relevant to phenotype discovery.

Seventh, although the post hoc VQ-VAE seed stability analysis showed low reconstruction errors and a stable overall control group-preserving assignment structure across three random initializations, codebook usage and control-distant subset membership remained partly seed-dependent. This indicates that downstream subgroup assignments depend on the learned discrete representation, particularly for the smaller control-distant subset. In addition, the present stability analysis varied the random initialization only; it did not exhaustively evaluate VQ-VAE hyperparameters such as the latent dimension, codebook size, commitment weight, or training duration. Future work should therefore perform broader representation-level sensitivity analyses, including both seed variation and systematic hyperparameter perturbations. Eighth, the spectral representation was resampled to a fixed 128-point grid to provide consistent input dimensionality for VQ-VAE encoding. This resampling should not be interpreted as increasing the independent frequency resolution of the recordings, which remained constrained by the task duration. Consequently, individual low-frequency points below the duration-limited resolution were not interpreted as independent oscillatory components; physiological interpretation was instead based on predefined frequency band summaries. Finally, although simulated annealing and tabu search provided matched same-BQM solver-level references, the present study did not establish a practical quantum speedup or end-to-end runtime advantage. These findings should therefore be interpreted as evidence of methodological feasibility and exploratory clinical context consistency, not as diagnostic proof of long COVID or as evidence of a practical quantum speedup.

Although an external diagnostic ground truth for post-COVID-19 balance impairment was not available, the control-distant assignments showed several internally consistent but exploratory patterns. The control-anchored BQM/QUBO formulation assigned very few control-group participant–contrast assignments to the control-distant group, suggesting that the inferred control-distant pattern was not simply a broad redistribution of latent space variability across all participants. The proportion of post-COVID-19 participants with a control-distant burden was also consistent with the expectation that persistent balance-related dysfunction would occur only in a subset of individuals rather than across the entire post-COVID-19 cohort. In addition, the control-distant assignments were accompanied by interpretable frequency band differences in postural control-related spectral components. The participant with a physician-documented long COVID evaluation note was also assigned to a control-distant subgroup across the BQM-based solvers and by the GMM clustering reference, providing only limited and anecdotal case-level clinical context consistency rather than evidence of diagnostic sensitivity or specificity. Taken together, these observations do not establish diagnostic validity, clinical sensitivity, or clinical specificity, but they provide convergent internal and exploratory clinical context motivation for further study of the control-distant balance subgroup.

## 5. Future Work

Future work should validate this framework in larger and more clinically characterized cohorts, including participants with physician-confirmed long COVID, standardized symptom inventories, vestibular and autonomic assessments, fatigue measures, and longitudinal follow-up. Such data would allow QUBO-derived control-distant balance profiles to be tested against clinically meaningful outcomes rather than interpreted only through exploratory clinical notes. Independent validation cohorts will also be needed to determine whether the frequency band phenotypes observed here are reproducible across sites, sensors, and participant populations. Future data collection protocols should also consider randomized or counterbalanced task ordering to separate cognitive load effects from repeated-task or order-related effects.

A second direction is the systematic optimization of the representation learning and QUBO components of the framework. For the VQ-VAE stage, future studies should extend the present seed stability analysis by evaluating broader hyperparameter sensitivity, including the latent dimension, codebook size, commitment weight, and training duration, because downstream control-distant assignments depend on the learned discrete latent representation. For the QUBO stage, future studies should evaluate sensitivity to the control-anchoring, sparsity, outlier reward, and graph smoothness weights and should investigate adaptive or data-driven strategies for selecting these parameters. In the present study, a single set of QUBO weights was used across all task contrasts. Lagrange weights could be adjusted according to the graph density, control distance separation, subgroup sparsity targets, or energy stability. In parallel, the relationship between QUBO energy and physiological interpretability should be tested using repeated solver runs, bootstrapped participant subsets, alternative graph constructions, and perturbation analyses.

The interpretability framework can also be expanded beyond band-averaged profiles. Although averaging across 17 joint pairs improved the readability, joint-resolved and body region-level analyses may reveal whether control-distant balance phenotypes preferentially involve distal lower-limb joints, trunk control, head–neck stabilization, or compensatory upper-body movement. Combining joint-resolved heatmaps with frequency band summaries could provide a more complete explanation of how latent control-distant assignments map onto neuromotor control strategies.

Finally, future work should evaluate whether quantum-assisted optimization can support scalable objective phenotyping across larger clinical populations and multi-site datasets. The present workflow was not designed to establish end-to-end quantum speedup, because preprocessing, VQ-VAE encoding, PCA, graph construction, minor embedding, CPU-QPU data transfer, queueing, and the hybrid workflow overhead all contribute to the practical runtime. Nevertheless, the annealing step itself is naturally suited to QUBO-formulated binary optimization problems, and future reductions in embedding overhead, data transfer bottlenecks, and hybrid orchestration costs could make quantum-assisted optimization increasingly practical for selected large-scale phenotyping tasks. Such scalability would be particularly valuable for deployment across many participants, repeated balance tasks, longitudinal visits, and multiple clinical sites. Future benchmarking should therefore compare quantum annealing, simulated annealing, tabu search, and classical mixed-integer optimization under matched QUBO formulations, reporting both the solver-level sampling behavior and end-to-end workflow runtime, to determine whether quantum-assisted optimization provides practical advantages or complementary sampling behavior for larger biomedical phenotyping datasets.

## 6. Conclusions

This study demonstrates the feasibility of using a control-anchored BQM/QUBO clustering framework to identify control-distant balance phenotypes in a heterogeneous post-COVID-19 cohort. By combining frequency-domain motion features, VQ-VAE latent representations, and graph-based binary optimization, the proposed framework identified a selective subset of post-COVID-19 participants with control-distant balance profiles while largely preserving the control-group reference structure.

Classical same-BQM solver analyses using simulated annealing and tabu search produced similar sparse, control group-preserving assignment patterns, suggesting that the observed subgroup structure was driven primarily by the control-anchored BQM/QUBO objective rather than by a single solver alone. Compared with GMM, which was retained as an unconstrained geometry-driven clustering reference, the control-anchored BQM/QUBO formulation produced fewer control-distant assignments among control-group participants and yielded frequency band profiles that appeared more interpretable in relation to postural control dynamics. The band-level analysis further suggested that control-distant profiles were most evident in low-frequency postural sway-dominant and mid–low-frequency postural control activity bands, supporting the physiological interpretability of the derived subgroups.

These findings should be interpreted as exploratory and hypothesis-generating rather than diagnostic validation or evidence of quantum speedup. Nevertheless, they highlight the potential of objective-driven, QUBO-based quantum-assisted clustering as a framework for subgroup discovery in label-limited biomedical datasets. With larger clinically characterized cohorts, systematic representation and QUBO parameter optimization, matched solver benchmarking, and multi-site validation, this approach may support interpretable post-COVID-19 neuromotor phenotyping and related medical data analysis applications.

## Figures and Tables

**Table 1:** Participant characteristics.

Characteristic	Analysis Cohort
Sample size, n	140
Age range, years	10–91
Age, years (mean ± SD)	44.0±18.4
Age missing, n	20
Sex, n (%)	
Male	54 (38.6%)
Female	82 (58.6%)
Not reported	4 (2.9%)
COVID-19 status, n (%)	
Post-COVID-19	114 (81.4%)
No known history of diagnosed COVID-19/control	26 (18.6%)
Age by sex, years (mean ± SD)	
Female	44.0±16.8
Male	43.0±20.4

Note: Age summary statistics were calculated using participants with valid age information. Post-COVID-19 status was determined based on documented prior SARS-CoV-2 infection confirmed using clinically accepted COVID-19 testing. Among the 26 control participants, 22 had documented negative COVID-19 testing at study enrollment, whereas 4 reported no known prior COVID-19 history but did not have prior diagnostic confirmation available. COVID-19 status was used for descriptive and comparative purposes only and was not used as a ground truth label in the control-anchored weakly supervised analytical framework.

**Table 2:** Sample availability across balance-derived task contrasts.

Task Contrast	Total, *n*	Control, *n*	Post-COVID-19, *n*
$\Delta S_{\mathrm{dual}-\mathrm{single}}$: Romberg (Open Eyes)	126	23	103
$\Delta S_{\mathrm{dual}-\mathrm{single}}$: Romberg (Closed Eyes)	132	24	108
$\Delta S_{\mathrm{dual}-\mathrm{single}}$: Tandem Romberg (Open Eyes)	130	24	106
$\Delta S_{\mathrm{dual}-\mathrm{single}}$: Tandem Romberg (Closed Eyes)	128	24	104
$\Delta S_{\mathrm{dual}-\mathrm{single}}$: CTSIB (Open Eyes)	127	23	104
$\Delta S_{\mathrm{dual}-\mathrm{single}}$: CTSIB (Closed Eyes)	123	22	101
$\Delta S_{\mathrm{dual}-\mathrm{single}}$: Fukuda	125	24	101
$\Delta S_{V,\mathrm{single}}$: Romberg	130	25	105
$\Delta S_{V,\mathrm{single}}$: Tandem Romberg	130	25	105
$\Delta S_{V,\mathrm{single}}$: CTSIB	131	24	107
$\Delta S_{V,\mathrm{dual}}$: Romberg	131	23	108
$\Delta S_{V,\mathrm{dual}}$: Tandem Romberg	132	24	108
$\Delta S_{V,\mathrm{dual}}$: CTSIB	128	22	106

Note: ∆*S*_dual–single_ denotes the cognitive dual-task versus single-task contrast for the same balance task, computed as cognitive dual-task minus single-task. ∆*S*_V,single_ denotes the closed-eyes versus open-eyes contrast under the single-task balance condition, and ∆*S*_V,dual_ denotes the corresponding closed-eyes versus open-eyes contrast under the cognitive dual-task balance condition; both visual condition contrasts were computed as closed-eyes minus open-eyes. Sample sizes varied across task contrasts because some participants did not complete all balance task conditions. Participant-level control-distant burden scores were normalized by the number of available task contrasts for each participant.

**Table 3:** Participant demographics by cohort.

Characteristic	Post-COVID-19	Control
Sample size, n	114	26
Valid age, n	96	24
Age missing or invalid, n	18	2
Age, years, mean ± SD	44.33±17.49	45.34±22.06
Age, years, median	43.78	49.86
Age range, years	10.16–87.84	11.28–91.56
Female, n (%)	70 (61.4%)	12 (46.2%)
Male, n (%)	41 (36.0%)	13 (50.0%)
Sex not reported, n (%)	3 (2.6%)	1 (3.8%)

Note: Age summary statistics were calculated using participants with valid age information. Age distributions did not differ significantly between cohorts under Mann–Whitney U testing (p=0.806) or Welch’s *t*-test (p=0.837). Sex distribution was compared among participants with reported male/female sex and did not differ significantly between cohorts under Fisher’s exact test (p=0.181). Demographic variables were used for cohort characterization only and were not included as inputs to the VQ-VAE, BQM/QUBO clustering, GMM clustering, or participant-level control-distant burden scoring procedures.

**Table 4:** QUBO optimization characteristics across balance-derived task contrasts.

Task Contrast	Variables	kNN Edges	RBF σ	QPU Sampled Energy	All-Zero Reference
$\Delta S_{\mathrm{dual}-\mathrm{single}}$: Romberg (Open)	126	2686	3.19	−9.72	0.00
$\Delta S_{\mathrm{dual}-\mathrm{single}}$: Romberg (Closed)	132	2944	2.36	1.70	0.00
$\Delta S_{\mathrm{dual}-\mathrm{single}}$: Tandem Romberg (Open)	130	2868	2.77	−12.66	0.00
$\Delta S_{\mathrm{dual}-\mathrm{single}}$: Tandem Romberg (Closed)	128	2891	2.71	−17.13	0.00
$\Delta S_{\mathrm{dual}-\mathrm{single}}$: CTSIB (Open)	127	2838	2.12	−11.43	0.00
$\Delta S_{\mathrm{dual}-\mathrm{single}}$: CTSIB (Closed)	123	2774	2.37	−8.96	0.00
$\Delta S_{\mathrm{dual}-\mathrm{single}}$: Fukuda	125	2687	3.56	−10.75	0.00
$\Delta S_{V,\mathrm{single}}$: Romberg	130	2871	2.15	−8.40	0.00
$\Delta S_{V,\mathrm{single}}$: Tandem Romberg	130	2919	2.82	−0.89	0.00
$\Delta S_{V,\mathrm{single}}$: CTSIB	131	2946	2.83	−7.56	0.00
$\Delta S_{V,\mathrm{dual}}$: Romberg	131	2866	2.75	−5.32	0.00
$\Delta S_{V,\mathrm{dual}}$: Tandem Romberg	132	2981	2.82	−9.12	0.00
$\Delta S_{V,\mathrm{dual}}$: CTSIB	128	2813	3.03	−4.66	0.00

Note: Each task contrast was formulated as a separate BQM. The number of variables corresponds to the number of available participants for that contrast. kNN edges correspond to RBF-weighted graph interactions included in the smoothness term. The QPU-sampled energy denotes the lowestenergy bitstring returned by the QPU sampler for that contrast. The all-zero reference denotes the feasible all-control-like assignment, in which all binary variables are set to *z_i_* = 0 and *E* = 0 under Equation (9). Energy values are contrast-specific QUBO objective values and are not interpreted as physiological severity scores across tasks. ∆*S*_dual−single_ denotes the cognitive dual-task versus single-task contrast for the same balance task. ∆*S*_V,single_ denotes the closed-eyes versus open-eyes contrast under the single-task balance condition, and ∆*S*_V,dual_ denotes the corresponding closed-eyes versus open-eyes contrast under the cognitive dual-task balance condition; both visual condition contrasts were computed as closed-eyes minus open-eyes. Negative QPU-sampled energies indicate an improvement over the all-zero reference under the specified QUBO objective. In contrast, a positive QPU-sampled energy indicates that the sampled solution did not improve upon this reference; therefore, the corresponding sampled assignment pattern should be interpreted cautiously from an optimization standpoint.

**Table 5:** Solver-level and GMM reference comparisons for control-distant assignments.

(a) Same-BQM solver-level comparison			
**Metric**	**Quantum annealing**	**Simulated annealing**	**Tabu search**
Control-group participant–contrast assignments labeled control-distant, n/N (%)	1/307 (0.3%)	0/307 (0.0%)	0/307 (0.0%)
Post-COVID-19 participant–contrast assignments labeled control-distant, n/N (%)	118/1366 (8.6%)	143/1366 (10.5%)	143/1366 (10.5%)
Control-group control-distant rate across contrasts	0.0–4.2%	0.0–0.0%	0.0–0.0%
Post-COVID-19 participant control-distant rate across contrasts	4.8–15.4%	5.9–21.2%	5.9–21.2%
Jaccard index versus quantum annealing	–	0.634	0.634
Jaccard index versus simulated annealing	0.634	–	1.000
**(b) GMM clustering reference comparison**			
**Metric**		**Quantum annealing**	**GMM**
Control-group participant–contrast assignments labeled control-distant, n/N (%)		1/307 (0.3%)	63/307 (20.5%)
Post-COVID-19 participant–contrast assignments labeled control-distant, n/N (%)		118/1366 (8.6%)	266/1366 (19.5%)
Control-group control-distant rate across contrasts		0.0–4.2%	4.5–37.5%
Post-COVID-19 participant control-distant rate across contrasts		4.8–15.4%	4.6–45.2%
Control-group participants with no control-distant contrasts, n/N (%)		25/26 (96.2%)	5/26 (19.2%)
Control-group participants classified as probable or strong control-distant, n/N (%)		0/26 (0.0%)	11/26 (42.3%)
Mean control-distant contrasts in control-group participants		0.04	2.42
Mean control-distant contrasts in post-COVID-19 participants		1.04	2.33

Note: Panel (a) compares solvers applied to the same contrast-specific BQM/QUBO formulations. Jaccard indices were computed using the control-distant assignment sets and are reported as solver-level agreement measures, not as evidence of solver superiority or quantum speedup. Panel (b) compares the quantum annealing QUBO solution with GMM as an unconstrained geometry-driven clustering reference. Participant–contrast summaries aggregate all available participant–contrast assignments across the 13 balance-derived task contrasts. Participant-level summaries are based on control-distant contrast counts normalized by the number of available task contrasts.

**Table 6:** Selected clinical note examples from participant-level QUBO, same-BQM solver, and GMM rankings.

Participant	Group	Clinical Note Summary	QA	SA	Tabu	GMM
PC013	Post-COVID-19	Physician-documented long COVID evaluation note	4/10; Probable control-distant	4/10; Probable control-distant	4/10; Probable control-distant	5/10; Probable control-distant
PC043	Post-COVID-19	No relevant symptom note available	6/13; Probable control-distant	8/13; Probable control-distant	8/13; Probable control-distant	11/13; Strong control-distant
PC080	Post-COVID-19	Chronic fatigue/long-hauler, autoimmune, cardiovascular notes	1/12; Single-test deviation	1/12; Single-test deviation	1/12; Single-test deviation	3/12; Probable control-distant
NC008	Control group	Control-group participant; limited available contrasts	1/7; Single-test deviation	0/7; Control-like	0/7; Control-like	7/7; Strong control-distant
NC003	Control group	No known COVID-19; not vaccinated	0/13; Control-like	0/13; Control-like	0/13; Control-like	5/13; Probable control-distant
NC018	Control group	Uncomfortable during balance testing; held wall	0/13; Control-like	0/13; Control-like	0/13; Control-like	3/13; Probable control-distant

Note: Clinical notes were incomplete and were not used as inputs to the VQ-VAE, quantum annealing, simulated annealing, tabu search, GMM clustering, or participant-level control-distant burden scoring. The selected cases illustrate concordant and discordant patterns between model-derived control-distant burden rankings and available clinical context. QA, SA, Tabu, and GMM values indicate the number of control-distant task contrasts over the number of available task contrasts, followed by the participant-level burden category. QA denotes the quantum annealing-sampled solution of the control-anchored QUBO formulation. SA and Tabu denote simulated annealing and tabu search applied to the same BQM/QUBO formulation. GMM denotes the unconstrained latent space clustering reference.

## Data Availability

The data that support the findings of this study are not publicly available due to ethical, privacy, confidentiality, and IRB restrictions involving human motion capture data and clinical context information. These restrictions apply to raw motion capture data, clinical metadata, participant-level derived features, and analysis materials that could be linked to the restricted human-subject dataset. To support reproducibility within these constraints, the manuscript reports the VQ-VAE architecture and training settings, BQM/QUBO construction, solver settings, and clustering summary procedures in detail. Additional reproducibility materials may be made available by the corresponding author only through appropriate institutional and IRB-approved data sharing mechanisms, where permitted.

## Supplementary Materials

The following supporting information can be downloaded at https://www.mdpi.com/article/10.3390/s26206556/s1. Table S1: Subgroup sample sizes and bootstrap 95% confidence intervals for band-averaged ΔEnergy profiles; Table S2: Control distance threshold baseline comparison; Table S3: Best BQM energies across quantum annealing, simulated annealing, and tabu search; Table S4: Parameter sensitivity analysis for QUBO-related settings.

## Abbreviations

The following abbreviations are used in this manuscript:

AIC	Akaike information criterion
BIC	Bayesian information criterion
BQM	Binary quadratic model
CTSIB	Clinical Test of Sensory Interaction on Balance
DFT	Discrete Fourier transform
GMM	Gaussian mixture modeling
IRB	Institutional Review Board
kNN	k-nearest neighbors
MSE	Mean squared error
NS	Standard single-task condition
PASC	Post-acute sequelae of COVID-19
PCA	Principal component analysis
QA	Quantum annealing
QPU	Quantum processing unit
QUBO	Quadratic unconstrained binary optimization
RBF	Radial basis function
RT-PCR	Reverse transcription polymerase chain reaction
SA	Simulated annealing
SOP	Sensory organization performance
ST	Cognitive dual-task condition; retained from the original task condition notation
SARS-CoV-2	Severe acute respiratory syndrome coronavirus 2
VQ-VAE	Vector-quantized variational autoencoder

## References

[1] Soriano J.B., Murthy S., Marshall J.C., Relan P., Diaz J.V. (2022). A clinical case definition of post-COVID-19 condition by a Delphi consensus. Lancet Infect. Dis..

[2] Proal A.D., VanElzakker M.B. (2021). Long COVID or Post-Acute Sequelae of COVID-19 (PASC): An Overview of Biological Factors That May Contribute to Persistent Symptoms. Front. Microbiol..

[3] Woodrow M., Carey C., Ziauddeen N., Thomas R., Akrami A., Lutje V., Greenwood D.C., Alwan N.A. (2023). Systematic review of the prevalence of long COVID. Proceedings of the Open Forum Infectious Diseases.

[4] Yılmaz O., Mutlu B.O., Yaman H., Bayazıt D., Demirhan H., Bayazıt Y.A. (2022). Assessment of balance after recovery from COVID-19 disease. Auris Nasus Larynx.

[5] Guzik A., Wolan-Nieroda A., Kochman M., Perenc L., Drużbicki M. (2022). Impact of mild COVID-19 on balance function in young adults, a prospective observational study. Sci. Rep..

[6] Winter D.A. (1995). Human Balance and Posture Control During Standing and Walking.

[7] Prieto T.E., Myklebust J.B., Hoffmann R.G., Lovett E.G., Myklebust B. (1996). Measures of postural steadiness: Differences between healthy young and elderly adults. IEEE Trans. Biomed. Eng..

[8] Pelicioni P.H.S., Santos A.D., Tako K.V., Santos P.C.R. (2021). COVID-19 and its impact on human motor control. Braz. J. Mot. Behav..

[9] Daniels K.A.J., Henderson G., Strike S., Cosgrave C., Fuller C., Falvey É. (2019). The use of continuous spectral analysis for the assessment of postural stability changes after sports-related concussion. J. Biomech..

[10] Gresty M., Buckwell D. (1990). Spectral analysis of tremor: Understanding the results. J. Neurol. Neurosurg. Psychiatry.

[11] Kunapinun A., Ellison P.J., Danesh A.A., Cai X.C., Levy X., Fields G.B., Pelah A. Joint-wise Spectral Analysis of Balance Responses Using Inverse Kinematics: A Motion Capture Study in Post-Acute COVID vs Non-COVID Individuals. In Proceedings of the 18th International Convention on Rehabilitation Engineering and Assistive Technology (i-CREATe 2025).

[12] Lasko T.A., Denny J.C., Levy M.A. (2013). Computational phenotype discovery using unsupervised feature learning over noisy, sparse, and irregular clinical data. PLoS ONE.

[13] Wu P.W., Bleecker M.E., Moore M.W., Busse M.W.W., Castro M.M., Chung M.K.F., Calhoun W.J., Erzurum S., Goston B., Israel E. (2014). Unsupervised phenotyping of severe asthma research program participants using expanded lung data. J. Allergy Clin. Immunol..

[14] Wang Y., Zhao Y., Therneau T.M., Atkinson E.J., Tafti A.P., Zhang N., Amin S., Limper A.H., Khosla S., Liu H. (2020). Unsupervised machine learning for the discovery of latent disease clusters and patient subgroups using electronic health records. J. Biomed. Inform..

[15] van den Oord A., Vinyals O., Kavukcuoglu K. Neural discrete representation learning. Proceedings of the 31st International Conference on Neural Information Processing Systems.

[16] Razavi A., van den Oord A., Vinyals O. Generating diverse high-fidelity images with VQ-VAE-2. Proceedings of the 33rd International Conference on Neural Information Processing Systems.

[17] Fortuin V., Hüser M., Locatello F., Strathmann H., Rätsch G. SOM-VAE: Interpretable Discrete Representation Learning on Time Series. Proceedings of the International Conference on Learning Representations.

[18] Bishop C.M. (2006). Pattern Recognition and Machine Learning.

[19] Nassir N., Hashmi M.A., Raji K.G., Jamalalail B., Maksymowsky A., Scherer S.W., Alsheikh-Ali A., Uddin M. (2025). Quantum computing and the implementation of precision medicine. npj Genom. Med..

[20] Nałkecz-Charkiewicz K., Charkiewicz K., Nowak R.M. (2024). Quantum computing in bioinformatics: A systematic review mapping. Brief. Bioinform..

[21] Ghazi Vakili M., Gorgulla C., Snider J., Nigam A., Bezrukov D., Varoli D., Aliper A., Polykovsky D., Padmanabha Das K.M., Cox H. (2025). Quantum-computing-enhanced algorithm unveils potential KRAS inhibitors. Nat. Biotechnol..

[22] Lucas A. (2014). Ising formulations of many NP problems. Front. Phys..

[23] Glover F., Kochenberger G., Du Y. (2018). A tutorial on formulating and using QUBO models. arXiv.

[24] Lubinski T., Coffrin C., McGeoch C., Sathe P., Apanavicius J., Bernal Neira D., Quantum Economic Development Consortium (2024). Optimization applications as quantum performance benchmarks. ACM Trans. Quantum Comput..

[25] Sijpesteijn T., Phillipson F. (2023). Quantum approaches for medoid clustering. Proceedings of the International Conference on Innovations for Community Services.

[26] Pérez Armas L.F., Creemers S., Deleplanque S. (2024). Solving the resource constrained project scheduling problem with quantum annealing. Sci. Rep..

[27] Oppenheim A.V., Schafer R.W., Buck J.R. (1999). Discrete-Time Signal Processing.

[28] Stoica P., Moses R.L. (2005). Spectral Analysis of Signals.

[29] Jolliffe I.T. (2002). Principal Component Analysis.

[30] Cover T.M., Hart P.E. (1967). Nearest neighbor pattern classification. IEEE Trans. Inf. Theory.

[31] Kirkpatrick S., Gelatt C.D., Vecchi M.P. (1983). Optimization by Simulated Annealing. Science.

[32] Glover F. (1989). Tabu Search—Part I. ORSA J. Comput..

