# Supplementary material for: Control-Anchored QUBO Clustering Reveals Control-Distant Postural Control Patterns in Motion Capture Balance Signals After COVID-19
Source: Sensors (Basel). 2026 Oct 15;26(20):6556. doi: 10.3390/s26206556 (PMC13708746; doi:10.3390/s26206556)
Supplement: Supplementary file 1 [file sensors-26-06556-s001.zip › sensors-4511317-supplementary.pdf]

Supplementary Materials

Control-Anchored QUBO Clustering Reveals Control-Distant Postural-Control Patterns in Motion-Capture Balance Signals After COVID-19

Supplementary Table S1

Supplementary Table S1 reports the number of participants contributing to each frequency-band subgroup profile and the corresponding bootstrap 95% confidence intervals for the band-averaged ΔEnergy values. Bootstrap confidence intervals were computed using 5000 participant-level bootstrap resamples within each subgroup, task contrast, clustering method, and frequency band. The task-contrast labels in this table follow the exported analysis identifiers: `DualTask-SingleTask` denotes the cognitive dual-task minus single-task contrast, and `ClosedMinusOpen` denotes the Closed-Eyes minus Open-Eyes visual-removal contrast. Labels containing `SOP` refer to single-task balance conditions, whereas labels containing `DualTask` refer to cognitive dual-task balance conditions.

Table S1: Subgroup sample sizes and bootstrap confidence intervals for frequency-band profiles.

Delta type	Task contrast	Method	Subgroup	Frequency band	n	Mean ΔEnergy (%)	CI lower	CI upper	Bootstrap n
DualTask-SingleTask	CTSIB_Closed_Eyes	Original	Original Control	Band1_PosturalSway	22	-2.188518	-8.575965	6.232565	5000
DualTask-SingleTask	CTSIB_Closed_Eyes	Original	Original Control	Band2_Normal	22	3.143324	-1.271986	7.451453	5000
DualTask-SingleTask	CTSIB_Closed_Eyes	Original	Original Control	Band3_Overcomp	22	-0.61779	-4.955174	2.884927	5000
DualTask-SingleTask	CTSIB_Closed_Eyes	Original	Original Control	Band4_Tremor	22	-0.337016	-1.539721	0.646525	5000
DualTask-SingleTask	CTSIB_Closed_Eyes	Original	Original post-COVID	Band1_PosturalSway	101	0.666773	-2.021855	3.465226	5000
DualTask-SingleTask	CTSIB_Closed_Eyes	Original	Original post-COVID	Band2_Normal	101	-0.804356	-3.402113	1.686357	5000
DualTask-SingleTask	CTSIB_Closed_Eyes	Original	Original post-COVID	Band3_Overcomp	101	-0.018038	-0.714989	0.559344	5000
DualTask-SingleTask	CTSIB_Closed_Eyes	Original	Original post-COVID	Band4_Tremor	101	0.155622	-0.025346	0.339113	5000
DualTask-SingleTask	CTSIB_Closed_Eyes	Quantum	QUBO post-COVID Control-like	Band1_PosturalSway	59	-1.117739	-4.025419	1.746401	5000
DualTask-SingleTask	CTSIB_Closed_Eyes	Quantum	QUBO post-COVID Control-like	Band2_Normal	59	1.459773	-1.013109	4.054716	5000
DualTask-SingleTask	CTSIB_Closed_Eyes	Quantum	QUBO post-COVID Control-like	Band3_Overcomp	59	-0.34924	-1.286153	0.344233	5000
DualTask-SingleTask	CTSIB_Closed_Eyes	Quantum	QUBO post-COVID Control-like	Band4_Tremor	59	0.007207	-0.136459	0.143429	5000
DualTask-SingleTask	CTSIB_Closed_Eyes	Quantum	QUBO post-COVID Control-distant	Band1_PosturalSway	42	3.173588	-1.982338	8.021488	5000
DualTask-SingleTask	CTSIB_Closed_Eyes	Quantum	QUBO post-COVID Control-distant	Band2_Normal	42	-3.984919	-8.518911	0.943275	5000
DualTask-SingleTask	CTSIB_Closed_Eyes	Quantum	QUBO post-COVID Control-distant	Band3_Overcomp	42	0.447222	-0.667189	1.359887	5000
DualTask-SingleTask	CTSIB_Closed_Eyes	Quantum	QUBO post-COVID Control-distant	Band4_Tremor	42	0.364109	0.000383	0.744744	5000
DualTask-SingleTask	CTSIB_Closed_Eyes	GMM	GMM post-COVID Control-like	Band1_PosturalSway	12	2.063218	-3.325972	7.434947	5000
DualTask-SingleTask	CTSIB_Closed_Eyes	GMM	GMM post-COVID Control-like	Band2_Normal	12	-2.167625	-7.862737	3.398945	5000
DualTask-SingleTask	CTSIB_Closed_Eyes	GMM	GMM post-COVID Control-like	Band3_Overcomp	12	0.049921	-0.343643	0.542155	5000
DualTask-SingleTask	CTSIB_Closed_Eyes	GMM	GMM post-COVID Control-like	Band4_Tremor	12	0.054486	-0.083259	0.232643	5000
DualTask-SingleTask	CTSIB_Closed_Eyes	GMM	GMM post-COVID Control-distant	Band1_PosturalSway	89	0.478488	-2.465057	3.488778	5000
DualTask-SingleTask	CTSIB_Closed_Eyes	GMM	GMM post-COVID Control-distant	Band2_Normal	89	-0.620545	-3.317837	2.081676	5000
DualTask-SingleTask	CTSIB_Closed_Eyes	GMM	GMM post-COVID Control-distant	Band3_Overcomp	89	-0.027201	-0.805734	0.647239	5000

Continued on next page

Supplementary Table S1 continued.

Delta type	Task contrast	Method	Subgroup	Frequency band	n	Mean ΔEnergy (%)	CI lower	CI upper	Bootstrap n
DualTask-SingleTask	CTSIB_Closed_Eyes	GMM	GMM post-COVID Control-distant	Band4_Tremor	89	0.169258	-0.023879	0.380373	5000
DualTask-SingleTask	CTSIB_Open_Eyes	Original	Original Control	Band1_PosturalSway	23	-2.842771	-6.952463	1.775957	5000
DualTask-SingleTask	CTSIB_Open_Eyes	Original	Original Control	Band2_Normal	23	1.341331	-2.171594	4.769607	5000
DualTask-SingleTask	CTSIB_Open_Eyes	Original	Original Control	Band3_Overcomp	23	1.28667	-0.534195	3.190687	5000
DualTask-SingleTask	CTSIB_Open_Eyes	Original	Original Control	Band4_Tremor	23	0.214769	-0.310591	0.717654	5000
DualTask-SingleTask	CTSIB_Open_Eyes	Original	Original post-COVID	Band1_PosturalSway	104	-2.064938	-4.937148	0.957406	5000
DualTask-SingleTask	CTSIB_Open_Eyes	Original	Original post-COVID	Band2_Normal	104	1.475413	-1.28839	4.121362	5000
DualTask-SingleTask	CTSIB_Open_Eyes	Original	Original post-COVID	Band3_Overcomp	104	0.403817	-0.28029	1.051796	5000
DualTask-SingleTask	CTSIB_Open_Eyes	Original	Original post-COVID	Band4_Tremor	104	0.185708	-0.021208	0.392728	5000
DualTask-SingleTask	CTSIB_Open_Eyes	Quantum	QUBO post-COVID Control-like	Band1_PosturalSway	60	-3.225326	-6.347611	0.163629	5000
DualTask-SingleTask	CTSIB_Open_Eyes	Quantum	QUBO post-COVID Control-like	Band2_Normal	60	2.756487	-0.542602	5.841404	5000
DualTask-SingleTask	CTSIB_Open_Eyes	Quantum	QUBO post-COVID Control-like	Band3_Overcomp	60	0.35411	-0.026104	0.7457	5000
DualTask-SingleTask	CTSIB_Open_Eyes	Quantum	QUBO post-COVID Control-like	Band4_Tremor	60	0.114729	-0.019669	0.249128	5000
DualTask-SingleTask	CTSIB_Open_Eyes	Quantum	QUBO post-COVID Control-distant	Band1_PosturalSway	44	-0.48259	-5.703616	5.389016	5000
DualTask-SingleTask	CTSIB_Open_Eyes	Quantum	QUBO post-COVID Control-distant	Band2_Normal	44	-0.271507	-5.250663	4.318226	5000
DualTask-SingleTask	CTSIB_Open_Eyes	Quantum	QUBO post-COVID Control-distant	Band3_Overcomp	44	0.471599	-1.069913	1.863395	5000
DualTask-SingleTask	CTSIB_Open_Eyes	Quantum	QUBO post-COVID Control-distant	Band4_Tremor	44	0.282498	-0.167963	0.717828	5000
DualTask-SingleTask	CTSIB_Open_Eyes	GMM	GMM post-COVID Control-like	Band1_PosturalSway	12	-3.079716	-7.841034	1.856685	5000
DualTask-SingleTask	CTSIB_Open_Eyes	GMM	GMM post-COVID Control-like	Band2_Normal	12	2.495495	-2.328831	7.321134	5000
DualTask-SingleTask	CTSIB_Open_Eyes	GMM	GMM post-COVID Control-like	Band3_Overcomp	12	0.392108	0.07285	0.72348	5000
DualTask-SingleTask	CTSIB_Open_Eyes	GMM	GMM post-COVID Control-like	Band4_Tremor	12	0.192113	0.076872	0.299674	5000
DualTask-SingleTask	CTSIB_Open_Eyes	GMM	GMM post-COVID Control-distant	Band1_PosturalSway	92	-1.932575	-5.140249	1.513047	5000
DualTask-SingleTask	CTSIB_Open_Eyes	GMM	GMM post-COVID Control-distant	Band2_Normal	92	1.342358	-1.731344	4.261322	5000
DualTask-SingleTask	CTSIB_Open_Eyes	GMM	GMM post-COVID Control-distant	Band3_Overcomp	92	0.405344	-0.361728	1.159238	5000

Continued on next page

Supplementary Table S1 continued.

Delta type	Task contrast	Method	Subgroup	Frequency band	n	Mean ΔEnergy (%)	CI lower	CI upper	Bootstrap n
DualTask-SingleTask	CTSIB_Open_Eyes	GMM	GMM post-COVID Control-distant	Band4_Tremor	92	0.184873	-0.048035	0.422561	5000
DualTask-SingleTask	Fukuda	Original	Original Control	Band1_PosturalSway	24	3.448749	-3.085453	10.380747	5000
DualTask-SingleTask	Fukuda	Original	Original Control	Band2_Normal	24	-1.038086	-6.857052	4.626316	5000
DualTask-SingleTask	Fukuda	Original	Original Control	Band3_Overcomp	24	-2.274397	-5.165842	0.267301	5000
DualTask-SingleTask	Fukuda	Original	Original Control	Band4_Tremor	24	-0.136266	-0.890232	0.599812	5000
DualTask-SingleTask	Fukuda	Original	Original post-COVID	Band1_PosturalSway	101	1.238138	-2.106201	4.689103	5000
DualTask-SingleTask	Fukuda	Original	Original post-COVID	Band2_Normal	101	-1.024583	-4.197215	1.972306	5000
DualTask-SingleTask	Fukuda	Original	Original post-COVID	Band3_Overcomp	101	-0.192269	-1.12496	0.747947	5000
DualTask-SingleTask	Fukuda	Original	Original post-COVID	Band4_Tremor	101	-0.021286	-0.350287	0.287646	5000
DualTask-SingleTask	Fukuda	Quantum	QUBO post-COVID Control-like	Band1_PosturalSway	60	0.451434	-3.847636	4.627357	5000
DualTask-SingleTask	Fukuda	Quantum	QUBO post-COVID Control-like	Band2_Normal	60	0.439579	-3.295128	4.290311	5000
DualTask-SingleTask	Fukuda	Quantum	QUBO post-COVID Control-like	Band3_Overcomp	60	-0.681953	-1.99079	0.58593	5000
DualTask-SingleTask	Fukuda	Quantum	QUBO post-COVID Control-like	Band4_Tremor	60	-0.20906	-0.682354	0.234695	5000
DualTask-SingleTask	Fukuda	Quantum	QUBO post-COVID Control-distant	Band1_PosturalSway	41	2.389412	-3.232368	8.339932	5000
DualTask-SingleTask	Fukuda	Quantum	QUBO post-COVID Control-distant	Band2_Normal	41	-3.167258	-8.32484	1.788587	5000
DualTask-SingleTask	Fukuda	Quantum	QUBO post-COVID Control-distant	Band3_Overcomp	41	0.524343	-0.747579	1.764725	5000
DualTask-SingleTask	Fukuda	Quantum	QUBO post-COVID Control-distant	Band4_Tremor	41	0.253504	-0.141773	0.639147	5000
DualTask-SingleTask	Fukuda	GMM	GMM post-COVID Control-like	Band1_PosturalSway	10	5.07704	-1.013706	10.613095	5000
DualTask-SingleTask	Fukuda	GMM	GMM post-COVID Control-like	Band2_Normal	10	-4.088424	-8.933626	0.521299	5000
DualTask-SingleTask	Fukuda	GMM	GMM post-COVID Control-like	Band3_Overcomp	10	-0.934006	-3.044885	1.016918	5000
DualTask-SingleTask	Fukuda	GMM	GMM post-COVID Control-like	Band4_Tremor	10	-0.05461	-1.0355	0.870091	5000
DualTask-SingleTask	Fukuda	GMM	GMM post-COVID Control-distant	Band1_PosturalSway	91	0.81628	-2.887038	4.607045	5000
DualTask-SingleTask	Fukuda	GMM	GMM post-COVID Control-distant	Band2_Normal	91	-0.687897	-4.089724	2.714546	5000
DualTask-SingleTask	Fukuda	GMM	GMM post-COVID Control-distant	Band3_Overcomp	91	-0.110759	-1.143588	0.862621	5000

Continued on next page

Supplementary Table S1 continued.

Delta type	Task contrast	Method	Subgroup	Frequency band	n	Mean ΔEnergy (%)	CI lower	CI upper	Bootstrap n
DualTask-SingleTask	Fukuda	GMM	GMM post-COVID Control-distant	Band4_Tremor	91	-0.017624	-0.375895	0.320766	5000
DualTask-SingleTask	Romberg_Closed_Eyes	Original	Original Control	Band1_PosturalSway	24	-3.478502	-8.942216	2.043693	5000
DualTask-SingleTask	Romberg_Closed_Eyes	Original	Original Control	Band2_Normal	24	1.028527	-4.237488	6.232827	5000
DualTask-SingleTask	Romberg_Closed_Eyes	Original	Original Control	Band3_Overcomp	24	1.726832	0.527248	3.396274	5000
DualTask-SingleTask	Romberg_Closed_Eyes	Original	Original Control	Band4_Tremor	24	0.723143	0.25964	1.295379	5000
DualTask-SingleTask	Romberg_Closed_Eyes	Original	Original post-COVID	Band1_PosturalSway	108	-0.03048	-2.744064	2.601149	5000
DualTask-SingleTask	Romberg_Closed_Eyes	Original	Original post-COVID	Band2_Normal	108	-0.720328	-3.231442	1.865468	5000
DualTask-SingleTask	Romberg_Closed_Eyes	Original	Original post-COVID	Band3_Overcomp	108	0.612544	0.054249	1.219677	5000
DualTask-SingleTask	Romberg_Closed_Eyes	Original	Original post-COVID	Band4_Tremor	108	0.19273	0.052117	0.335935	5000
DualTask-SingleTask	Romberg_Closed_Eyes	Quantum	QUBO post-COVID Control-like	Band1_PosturalSway	65	2.79597	-0.16637	5.771909	5000
DualTask-SingleTask	Romberg_Closed_Eyes	Quantum	QUBO post-COVID Control-like	Band2_Normal	65	-3.198034	-5.938226	-0.460229	5000
DualTask-SingleTask	Romberg_Closed_Eyes	Quantum	QUBO post-COVID Control-like	Band3_Overcomp	65	0.348897	-0.389846	1.037507	5000
DualTask-SingleTask	Romberg_Closed_Eyes	Quantum	QUBO post-COVID Control-like	Band4_Tremor	65	0.143665	0.030539	0.268249	5000
DualTask-SingleTask	Romberg_Closed_Eyes	Quantum	QUBO post-COVID Control-distant	Band1_PosturalSway	43	-4.303022	-9.217308	0.329598	5000
DualTask-SingleTask	Romberg_Closed_Eyes	Quantum	QUBO post-COVID Control-distant	Band2_Normal	43	3.025043	-1.583062	7.890755	5000
DualTask-SingleTask	Romberg_Closed_Eyes	Quantum	QUBO post-COVID Control-distant	Band3_Overcomp	43	1.011081	0.11216	2.158094	5000
DualTask-SingleTask	Romberg_Closed_Eyes	Quantum	QUBO post-COVID Control-distant	Band4_Tremor	43	0.266898	-0.036565	0.597233	5000
DualTask-SingleTask	Romberg_Closed_Eyes	GMM	GMM post-COVID Control-like	Band1_PosturalSway	15	-0.775899	-5.236659	3.866128	5000
DualTask-SingleTask	Romberg_Closed_Eyes	GMM	GMM post-COVID Control-like	Band2_Normal	15	-0.286936	-4.733989	4.104911	5000
DualTask-SingleTask	Romberg_Closed_Eyes	GMM	GMM post-COVID Control-like	Band3_Overcomp	15	0.769786	0.255854	1.360849	5000
DualTask-SingleTask	Romberg_Closed_Eyes	GMM	GMM post-COVID Control-like	Band4_Tremor	15	0.293048	0.06271	0.554331	5000
DualTask-SingleTask	Romberg_Closed_Eyes	GMM	GMM post-COVID Control-distant	Band1_PosturalSway	93	0.089748	-2.959433	3.077495	5000
DualTask-SingleTask	Romberg_Closed_Eyes	GMM	GMM post-COVID Control-distant	Band2_Normal	93	-0.790229	-3.60639	2.023532	5000
DualTask-SingleTask	Romberg_Closed_Eyes	GMM	GMM post-COVID Control-distant	Band3_Overcomp	93	0.587182	-0.068087	1.286309	5000

Continued on next page

Supplementary Table S1 continued.

Delta type	Task contrast	Method	Subgroup	Frequency band	n	Mean ΔEnergy (%)	CI lower	CI upper	Bootstrap n
DualTask-SingleTask	Romberg_Closed_Eyes	GMM	GMM post-COVID Control-distant	Band4_Tremor	93	0.17655	0.017374	0.339886	5000
DualTask-SingleTask	Romberg_Open_Eyes	Original	Original Control	Band1_PosturalSway	23	-2.307505	-8.551614	3.86794	5000
DualTask-SingleTask	Romberg_Open_Eyes	Original	Original Control	Band2_Normal	23	-0.455709	-5.437581	4.534075	5000
DualTask-SingleTask	Romberg_Open_Eyes	Original	Original Control	Band3_Overcomp	23	2.427487	0.171945	5.039702	5000
DualTask-SingleTask	Romberg_Open_Eyes	Original	Original Control	Band4_Tremor	23	0.335727	-0.141877	0.819871	5000
DualTask-SingleTask	Romberg_Open_Eyes	Original	Original post-COVID	Band1_PosturalSway	103	-2.044084	-5.071288	0.86461	5000
DualTask-SingleTask	Romberg_Open_Eyes	Original	Original post-COVID	Band2_Normal	103	1.272706	-1.398566	4.044238	5000
DualTask-SingleTask	Romberg_Open_Eyes	Original	Original post-COVID	Band3_Overcomp	103	0.63503	0.113493	1.201583	5000
DualTask-SingleTask	Romberg_Open_Eyes	Original	Original post-COVID	Band4_Tremor	103	0.193459	-0.021822	0.419988	5000
DualTask-SingleTask	Romberg_Open_Eyes	Quantum	QUBO post-COVID Control-like	Band1_PosturalSway	60	-1.625246	-5.162541	1.842408	5000
DualTask-SingleTask	Romberg_Open_Eyes	Quantum	QUBO post-COVID Control-like	Band2_Normal	60	1.245717	-2.089536	4.59977	5000
DualTask-SingleTask	Romberg_Open_Eyes	Quantum	QUBO post-COVID Control-like	Band3_Overcomp	60	0.343373	-0.136401	0.859932	5000
DualTask-SingleTask	Romberg_Open_Eyes	Quantum	QUBO post-COVID Control-like	Band4_Tremor	60	0.134195	-0.028729	0.306823	5000
DualTask-SingleTask	Romberg_Open_Eyes	Quantum	QUBO post-COVID Control-distant	Band1_PosturalSway	43	-2.62851	-7.919001	2.39859	5000
DualTask-SingleTask	Romberg_Open_Eyes	Quantum	QUBO post-COVID Control-distant	Band2_Normal	43	1.310365	-3.245843	6.049865	5000
DualTask-SingleTask	Romberg_Open_Eyes	Quantum	QUBO post-COVID Control-distant	Band3_Overcomp	43	1.041994	0.002012	2.261445	5000
DualTask-SingleTask	Romberg_Open_Eyes	Quantum	QUBO post-COVID Control-distant	Band4_Tremor	43	0.276152	-0.196318	0.765154	5000
DualTask-SingleTask	Romberg_Open_Eyes	GMM	GMM post-COVID Control-like	Band1_PosturalSway	14	-1.406406	-6.005762	3.584691	5000
DualTask-SingleTask	Romberg_Open_Eyes	GMM	GMM post-COVID Control-like	Band2_Normal	14	0.712191	-4.319841	5.293203	5000
DualTask-SingleTask	Romberg_Open_Eyes	GMM	GMM post-COVID Control-like	Band3_Overcomp	14	0.434004	-0.352247	1.285017	5000
DualTask-SingleTask	Romberg_Open_Eyes	GMM	GMM post-COVID Control-like	Band4_Tremor	14	0.26021	-0.15796	0.780809	5000
DualTask-SingleTask	Romberg_Open_Eyes	GMM	GMM post-COVID Control-distant	Band1_PosturalSway	89	-2.144393	-5.501106	1.128299	5000
DualTask-SingleTask	Romberg_Open_Eyes	GMM	GMM post-COVID Control-distant	Band2_Normal	89	1.360877	-1.653737	4.405505	5000
DualTask-SingleTask	Romberg_Open_Eyes	GMM	GMM post-COVID Control-distant	Band3_Overcomp	89	0.666652	0.078946	1.33513	5000

Continued on next page

Supplementary Table S1 continued.

Delta type	Task contrast	Method	Subgroup	Frequency band	n	Mean ΔEnergy (%)	CI lower	CI upper	Bootstrap n
DualTask-SingleTask	Romberg_Open_Eyes	GMM	GMM post-COVID Control-distant	Band4_Tremor	89	0.182958	-0.053834	0.438344	5000
DualTask-SingleTask	Tandem_Romberg_Closed_Eyes	Original	Original Control	Band1_PosturalSway	24	-1.844492	-7.106274	4.138931	5000
DualTask-SingleTask	Tandem_Romberg_Closed_Eyes	Original	Original Control	Band2_Normal	24	0.073156	-3.497252	3.641807	5000
DualTask-SingleTask	Tandem_Romberg_Closed_Eyes	Original	Original Control	Band3_Overcomp	24	1.482079	-2.825515	5.757377	5000
DualTask-SingleTask	Tandem_Romberg_Closed_Eyes	Original	Original Control	Band4_Tremor	24	0.289257	-0.977305	1.584269	5000
DualTask-SingleTask	Tandem_Romberg_Closed_Eyes	Original	Original post-COVID	Band1_PosturalSway	104	-0.690572	-4.163846	2.877883	5000
DualTask-SingleTask	Tandem_Romberg_Closed_Eyes	Original	Original post-COVID	Band2_Normal	104	0.624424	-1.668306	2.824489	5000
DualTask-SingleTask	Tandem_Romberg_Closed_Eyes	Original	Original post-COVID	Band3_Overcomp	104	-0.002786	-2.100944	2.016999	5000
DualTask-SingleTask	Tandem_Romberg_Closed_Eyes	Original	Original post-COVID	Band4_Tremor	104	0.068934	-0.374725	0.540288	5000
DualTask-SingleTask	Tandem_Romberg_Closed_Eyes	Quantum	QUBO post-COVID Control-like	Band1_PosturalSway	62	-0.021983	-3.811675	3.793334	5000
DualTask-SingleTask	Tandem_Romberg_Closed_Eyes	Quantum	QUBO post-COVID Control-like	Band2_Normal	62	0.964985	-1.770952	3.744803	5000
DualTask-SingleTask	Tandem_Romberg_Closed_Eyes	Quantum	QUBO post-COVID Control-like	Band3_Overcomp	62	-0.938214	-2.851317	1.025831	5000
DualTask-SingleTask	Tandem_Romberg_Closed_Eyes	Quantum	QUBO post-COVID Control-like	Band4_Tremor	62	-0.004788	-0.384923	0.444304	5000
DualTask-SingleTask	Tandem_Romberg_Closed_Eyes	Quantum	QUBO post-COVID Control-distant	Band1_PosturalSway	42	-1.677536	-8.632204	5.291357	5000
DualTask-SingleTask	Tandem_Romberg_Closed_Eyes	Quantum	QUBO post-COVID Control-distant	Band2_Normal	42	0.121691	-3.901414	3.944018	5000
DualTask-SingleTask	Tandem_Romberg_Closed_Eyes	Quantum	QUBO post-COVID Control-distant	Band3_Overcomp	42	1.378083	-2.907046	5.505332	5000
DualTask-SingleTask	Tandem_Romberg_Closed_Eyes	Quantum	QUBO post-COVID Control-distant	Band4_Tremor	42	0.177762	-0.834575	1.110103	5000
DualTask-SingleTask	Tandem_Romberg_Closed_Eyes	GMM	GMM post-COVID Control-like	Band1_PosturalSway	12	-10.430114	-18.822062	-3.793757	5000
DualTask-SingleTask	Tandem_Romberg_Closed_Eyes	GMM	GMM post-COVID Control-like	Band2_Normal	12	4.628971	1.292236	8.609017	5000
DualTask-SingleTask	Tandem_Romberg_Closed_Eyes	GMM	GMM post-COVID Control-like	Band3_Overcomp	12	5.297118	0.786673	11.587033	5000
DualTask-SingleTask	Tandem_Romberg_Closed_Eyes	GMM	GMM post-COVID Control-like	Band4_Tremor	12	0.504025	-0.288579	1.678889	5000
DualTask-SingleTask	Tandem_Romberg_Closed_Eyes	GMM	GMM post-COVID Control-distant	Band1_PosturalSway	92	0.579804	-3.324436	4.472544	5000
DualTask-SingleTask	Tandem_Romberg_Closed_Eyes	GMM	GMM post-COVID Control-distant	Band2_Normal	92	0.102091	-2.383678	2.665407	5000
DualTask-SingleTask	Tandem_Romberg_Closed_Eyes	GMM	GMM post-COVID Control-distant	Band3_Overcomp	92	-0.694078	-2.979736	1.472462	5000

Continued on next page

Supplementary Table S1 continued.

Delta type	Task contrast	Method	Subgroup	Frequency band	n	Mean ΔEnergy (%)	CI lower	CI upper	Bootstrap n
DualTask-SingleTask	Tandem_Romberg_Closed_Eyes	GMM	GMM post-COVID Control-distant	Band4_Tremor	92	0.012183	-0.505453	0.515415	5000
DualTask-SingleTask	Tandem_Romberg_Open_Eyes	Original	Original Control	Band1_PosturalSway	24	2.973391	-5.215186	11.350083	5000
DualTask-SingleTask	Tandem_Romberg_Open_Eyes	Original	Original Control	Band2_Normal	24	-2.817958	-8.460052	2.197946	5000
DualTask-SingleTask	Tandem_Romberg_Open_Eyes	Original	Original Control	Band3_Overcomp	24	-0.736371	-4.559271	3.462113	5000
DualTask-SingleTask	Tandem_Romberg_Open_Eyes	Original	Original Control	Band4_Tremor	24	0.580938	-0.067001	1.353344	5000
DualTask-SingleTask	Tandem_Romberg_Open_Eyes	Original	Original post-COVID	Band1_PosturalSway	106	-2.646095	-6.12399	0.735712	5000
DualTask-SingleTask	Tandem_Romberg_Open_Eyes	Original	Original post-COVID	Band2_Normal	106	1.556738	-0.807205	3.982158	5000
DualTask-SingleTask	Tandem_Romberg_Open_Eyes	Original	Original post-COVID	Band3_Overcomp	106	0.744165	-0.870591	2.358514	5000
DualTask-SingleTask	Tandem_Romberg_Open_Eyes	Original	Original post-COVID	Band4_Tremor	106	0.400686	0.116865	0.724187	5000
DualTask-SingleTask	Tandem_Romberg_Open_Eyes	Quantum	QUBO post-COVID Control-like	Band1_PosturalSway	63	-0.226716	-4.519626	4.086903	5000
DualTask-SingleTask	Tandem_Romberg_Open_Eyes	Quantum	QUBO post-COVID Control-like	Band2_Normal	63	-0.693896	-3.767462	2.454621	5000
DualTask-SingleTask	Tandem_Romberg_Open_Eyes	Quantum	QUBO post-COVID Control-like	Band3_Overcomp	63	0.616141	-1.537047	2.704197	5000
DualTask-SingleTask	Tandem_Romberg_Open_Eyes	Quantum	QUBO post-COVID Control-like	Band4_Tremor	63	0.397841	0.028495	0.879549	5000
DualTask-SingleTask	Tandem_Romberg_Open_Eyes	Quantum	QUBO post-COVID Control-distant	Band1_PosturalSway	43	-6.190767	-11.81736	-0.920824	5000
DualTask-SingleTask	Tandem_Romberg_Open_Eyes	Quantum	QUBO post-COVID Control-distant	Band2_Normal	43	4.854178	1.389977	8.587533	5000
DualTask-SingleTask	Tandem_Romberg_Open_Eyes	Quantum	QUBO post-COVID Control-distant	Band3_Overcomp	43	0.931734	-1.440348	3.380808	5000
DualTask-SingleTask	Tandem_Romberg_Open_Eyes	Quantum	QUBO post-COVID Control-distant	Band4_Tremor	43	0.404854	0.00716	0.820249	5000
DualTask-SingleTask	Tandem_Romberg_Open_Eyes	GMM	GMM post-COVID Control-like	Band1_PosturalSway	14	2.772174	-4.943707	11.496844	5000
DualTask-SingleTask	Tandem_Romberg_Open_Eyes	GMM	GMM post-COVID Control-like	Band2_Normal	14	-0.520735	-5.786203	5.038279	5000
DualTask-SingleTask	Tandem_Romberg_Open_Eyes	GMM	GMM post-COVID Control-like	Band3_Overcomp	14	-2.23451	-6.610811	2.117746	5000
DualTask-SingleTask	Tandem_Romberg_Open_Eyes	GMM	GMM post-COVID Control-like	Band4_Tremor	14	-0.016929	-0.323184	0.306271	5000
DualTask-SingleTask	Tandem_Romberg_Open_Eyes	GMM	GMM post-COVID Control-distant	Band1_PosturalSway	92	-3.470614	-7.152168	0.143203	5000
DualTask-SingleTask	Tandem_Romberg_Open_Eyes	GMM	GMM post-COVID Control-distant	Band2_Normal	92	1.872875	-0.78591	4.557825	5000
DualTask-SingleTask	Tandem_Romberg_Open_Eyes	GMM	GMM post-COVID Control-distant	Band3_Overcomp	92	1.197442	-0.499841	2.914757	5000

Continued on next page

Supplementary Table S1 continued.

Delta type	Task contrast	Method	Subgroup	Frequency band	n	Mean ΔEnergy (%)	CI lower	CI upper	Bootstrap n
DualTask-SingleTask	Tandem_Romberg_-Open_Eyes	GMM	GMM post-COVID Control-distant	Band4_Tremor	92	0.464236	0.144094	0.838446	5000
ClosedMinusOpen	SOP_CTSIB	Original	Original Control	Band1_PosturalSway	24	-2.216102	-9.425504	4.488657	5000
ClosedMinusOpen	SOP_CTSIB	Original	Original Control	Band2_Normal	24	1.588588	-3.943078	7.432673	5000
ClosedMinusOpen	SOP_CTSIB	Original	Original Control	Band3_Overcomp	24	0.4603	-2.388642	4.470036	5000
ClosedMinusOpen	SOP_CTSIB	Original	Original Control	Band4_Tremor	24	0.167214	-0.780883	1.3757	5000
ClosedMinusOpen	SOP_CTSIB	Original	Original post-COVID	Band1_PosturalSway	107	0.161405	-2.924655	3.293572	5000
ClosedMinusOpen	SOP_CTSIB	Original	Original post-COVID	Band2_Normal	107	-0.197444	-3.002804	2.581917	5000
ClosedMinusOpen	SOP_CTSIB	Original	Original post-COVID	Band3_Overcomp	107	0.071435	-0.702442	0.824928	5000
ClosedMinusOpen	SOP_CTSIB	Original	Original post-COVID	Band4_Tremor	107	-0.035395	-0.244812	0.149922	5000
ClosedMinusOpen	SOP_CTSIB	Quantum	QUBO post-COVID Control-like	Band1_PosturalSway	62	-1.681531	-5.600403	2.435145	5000
ClosedMinusOpen	SOP_CTSIB	Quantum	QUBO post-COVID Control-like	Band2_Normal	62	0.943708	-2.856116	4.532153	5000
ClosedMinusOpen	SOP_CTSIB	Quantum	QUBO post-COVID Control-like	Band3_Overcomp	62	0.665603	-0.093541	1.604317	5000
ClosedMinusOpen	SOP_CTSIB	Quantum	QUBO post-COVID Control-like	Band4_Tremor	62	0.072219	-0.054487	0.209746	5000
ClosedMinusOpen	SOP_CTSIB	Quantum	QUBO post-COVID Control-distant	Band1_PosturalSway	45	2.70056	-1.929874	7.748361	5000
ClosedMinusOpen	SOP_CTSIB	Quantum	QUBO post-COVID Control-distant	Band2_Normal	45	-1.769699	-6.093409	2.436865	5000
ClosedMinusOpen	SOP_CTSIB	Quantum	QUBO post-COVID Control-distant	Band3_Overcomp	45	-0.747197	-2.197858	0.548444	5000
ClosedMinusOpen	SOP_CTSIB	Quantum	QUBO post-COVID Control-distant	Band4_Tremor	45	-0.183665	-0.639253	0.218578	5000
ClosedMinusOpen	SOP_CTSIB	GMM	GMM post-COVID Control-like	Band1_PosturalSway	15	-0.399872	-6.531882	6.633201	5000
ClosedMinusOpen	SOP_CTSIB	GMM	GMM post-COVID Control-like	Band2_Normal	15	0.300815	-6.463828	6.14222	5000
ClosedMinusOpen	SOP_CTSIB	GMM	GMM post-COVID Control-like	Band3_Overcomp	15	0.054936	-0.278552	0.406912	5000
ClosedMinusOpen	SOP_CTSIB	GMM	GMM post-COVID Control-like	Band4_Tremor	15	0.044121	-0.03541	0.121197	5000
ClosedMinusOpen	SOP_CTSIB	GMM	GMM post-COVID Control-distant	Band1_PosturalSway	92	0.252917	-3.113919	3.746205	5000
ClosedMinusOpen	SOP_CTSIB	GMM	GMM post-COVID Control-distant	Band2_Normal	92	-0.278682	-3.396055	2.835377	5000
ClosedMinusOpen	SOP_CTSIB	GMM	GMM post-COVID Control-distant	Band3_Overcomp	92	0.074125	-0.825507	0.945584	5000
ClosedMinusOpen	SOP_CTSIB	GMM	GMM post-COVID Control-distant	Band4_Tremor	92	-0.04836	-0.287744	0.17269	5000
ClosedMinusOpen	SOP_Romberg	Original	Original Control	Band1_PosturalSway	25	2.388674	-3.612518	8.727576	5000
ClosedMinusOpen	SOP_Romberg	Original	Original Control	Band2_Normal	25	-1.298381	-6.881414	4.197745	5000
ClosedMinusOpen	SOP_Romberg	Original	Original Control	Band3_Overcomp	25	-0.8106	-1.821366	-0.050929	5000
ClosedMinusOpen	SOP_Romberg	Original	Original Control	Band4_Tremor	25	-0.279693	-0.626189	-0.03745	5000
ClosedMinusOpen	SOP_Romberg	Original	Original post-COVID	Band1_PosturalSway	105	-0.008102	-2.380149	2.423861	5000
ClosedMinusOpen	SOP_Romberg	Original	Original post-COVID	Band2_Normal	105	0.646415	-1.672672	2.921668	5000

Continued on next page

Supplementary Table S1 continued.

Delta type	Task contrast	Method	Subgroup	Frequency band	n	Mean ΔEnergy (%)	CI lower	CI upper	Bootstrap n
ClosedMinusOpen	SOP_Romberg	Original	Original post-COVID	Band3_Overcomp	105	-0.433013	-0.796513	-0.020591	5000
ClosedMinusOpen	SOP_Romberg	Original	Original post-COVID	Band4_Tremor	105	-0.2053	-0.367917	-0.073111	5000
ClosedMinusOpen	SOP_Romberg	Quantum	QUBO post-COVID Control-like	Band1_PosturalSway	61	-2.181171	-5.159024	0.756317	5000
ClosedMinusOpen	SOP_Romberg	Quantum	QUBO post-COVID Control-like	Band2_Normal	61	2.509487	-0.178045	5.281748	5000
ClosedMinusOpen	SOP_Romberg	Quantum	QUBO post-COVID Control-like	Band3_Overcomp	61	-0.212515	-0.686286	0.385254	5000
ClosedMinusOpen	SOP_Romberg	Quantum	QUBO post-COVID Control-like	Band4_Tremor	61	-0.115801	-0.222534	-0.017204	5000
ClosedMinusOpen	SOP_Romberg	Quantum	QUBO post-COVID Control-distant	Band1_PosturalSway	44	3.004561	-0.972292	6.892224	5000
ClosedMinusOpen	SOP_Romberg	Quantum	QUBO post-COVID Control-distant	Band2_Normal	44	-1.93648	-5.803165	2.076816	5000
ClosedMinusOpen	SOP_Romberg	Quantum	QUBO post-COVID Control-distant	Band3_Overcomp	44	-0.738703	-1.311928	-0.267999	5000
ClosedMinusOpen	SOP_Romberg	Quantum	QUBO post-COVID Control-distant	Band4_Tremor	44	-0.329378	-0.678497	-0.061315	5000
ClosedMinusOpen	SOP_Romberg	GMM	GMM post-COVID Control-like	Band1_PosturalSway	15	1.31427	-2.584189	5.682607	5000
ClosedMinusOpen	SOP_Romberg	GMM	GMM post-COVID Control-like	Band2_Normal	15	-0.69729	-5.043937	3.047461	5000
ClosedMinusOpen	SOP_Romberg	GMM	GMM post-COVID Control-like	Band3_Overcomp	15	-0.455884	-0.861291	-0.091983	5000
ClosedMinusOpen	SOP_Romberg	GMM	GMM post-COVID Control-like	Band4_Tremor	15	-0.161096	-0.367944	-0.000991	5000
ClosedMinusOpen	SOP_Romberg	GMM	GMM post-COVID Control-distant	Band1_PosturalSway	90	-0.228498	-2.952991	2.412559	5000
ClosedMinusOpen	SOP_Romberg	GMM	GMM post-COVID Control-distant	Band2_Normal	90	0.870366	-1.670933	3.431744	5000
ClosedMinusOpen	SOP_Romberg	GMM	GMM post-COVID Control-distant	Band3_Overcomp	90	-0.429201	-0.84329	0.038528	5000
ClosedMinusOpen	SOP_Romberg	GMM	GMM post-COVID Control-distant	Band4_Tremor	90	-0.212667	-0.390726	-0.066089	5000
ClosedMinusOpen	SOP_Tandem_-Romberg	Original	Original Control	Band1_PosturalSway	25	0.320793	-6.427681	6.954929	5000
ClosedMinusOpen	SOP_Tandem_-Romberg	Original	Original Control	Band2_Normal	25	-0.151503	-3.68863	3.572648	5000
ClosedMinusOpen	SOP_Tandem_-Romberg	Original	Original Control	Band3_Overcomp	25	-0.636284	-4.924049	4.082132	5000
ClosedMinusOpen	SOP_Tandem_-Romberg	Original	Original Control	Band4_Tremor	25	0.466993	-0.325537	1.417395	5000
ClosedMinusOpen	SOP_Tandem_-Romberg	Original	Original post-COVID	Band1_PosturalSway	105	-0.309196	-3.397892	2.63149	5000
ClosedMinusOpen	SOP_Tandem_-Romberg	Original	Original post-COVID	Band2_Normal	105	-1.106025	-3.215559	1.075734	5000
ClosedMinusOpen	SOP_Tandem_-Romberg	Original	Original post-COVID	Band3_Overcomp	105	0.937026	-0.655927	2.577734	5000

Continued on next page

Supplementary Table S1 continued.

Delta type	Task contrast	Method	Subgroup	Frequency band	n	Mean ΔEnergy (%)	CI lower	CI upper	Bootstrap n
ClosedMinusOpen	SOP_Tandem_Romberg	Original	Original post-COVID	Band4_Tremor	105	0.534218	0.209899	0.926455	5000
ClosedMinusOpen	SOP_Tandem_Romberg	Quantum	QUBO post-COVID Control-like	Band1_PosturalSway	64	1.19332	-2.417449	5.005791	5000
ClosedMinusOpen	SOP_Tandem_Romberg	Quantum	QUBO post-COVID Control-like	Band2_Normal	64	-1.564376	-4.157448	1.076973	5000
ClosedMinusOpen	SOP_Tandem_Romberg	Quantum	QUBO post-COVID Control-like	Band3_Overcomp	64	0.281813	-1.520698	2.139923	5000
ClosedMinusOpen	SOP_Tandem_Romberg	Quantum	QUBO post-COVID Control-like	Band4_Tremor	64	0.181155	-0.053177	0.426029	5000
ClosedMinusOpen	SOP_Tandem_Romberg	Quantum	QUBO post-COVID Control-distant	Band1_PosturalSway	41	-2.654587	-7.783506	2.121311	5000
ClosedMinusOpen	SOP_Tandem_Romberg	Quantum	QUBO post-COVID Control-distant	Band2_Normal	41	-0.390552	-3.742076	3.372071	5000
ClosedMinusOpen	SOP_Tandem_Romberg	Quantum	QUBO post-COVID Control-distant	Band3_Overcomp	41	1.959797	-0.799137	5.009808	5000
ClosedMinusOpen	SOP_Tandem_Romberg	Quantum	QUBO post-COVID Control-distant	Band4_Tremor	41	1.085341	0.35288	1.985153	5000
ClosedMinusOpen	SOP_Tandem_Romberg	GMM	GMM post-COVID Control-like	Band1_PosturalSway	14	6.934251	-1.507046	15.257035	5000
ClosedMinusOpen	SOP_Tandem_Romberg	GMM	GMM post-COVID Control-like	Band2_Normal	14	-4.852492	-11.944336	2.244599	5000
ClosedMinusOpen	SOP_Tandem_Romberg	GMM	GMM post-COVID Control-like	Band3_Overcomp	14	-2.140552	-5.321621	0.816524	5000
ClosedMinusOpen	SOP_Tandem_Romberg	GMM	GMM post-COVID Control-like	Band4_Tremor	14	0.058794	-0.546586	0.723116	5000
ClosedMinusOpen	SOP_Tandem_Romberg	GMM	GMM post-COVID Control-distant	Band1_PosturalSway	91	-1.423572	-4.693273	1.769741	5000
ClosedMinusOpen	SOP_Tandem_Romberg	GMM	GMM post-COVID Control-distant	Band2_Normal	91	-0.529646	-2.765776	1.753629	5000
ClosedMinusOpen	SOP_Tandem_Romberg	GMM	GMM post-COVID Control-distant	Band3_Overcomp	91	1.410499	-0.328013	3.223086	5000
ClosedMinusOpen	SOP_Tandem_Romberg	GMM	GMM post-COVID Control-distant	Band4_Tremor	91	0.60736	0.230603	1.046052	5000
ClosedMinusOpen	Stroop_CTSIB	Original	Original Control	Band1_PosturalSway	22	-1.677421	-8.313219	5.119121	5000
ClosedMinusOpen	Stroop_CTSIB	Original	Original Control	Band2_Normal	22	2.170269	-4.029546	7.992317	5000
ClosedMinusOpen	Stroop_CTSIB	Original	Original Control	Band3_Overcomp	22	-0.169376	-1.679701	1.794269	5000
ClosedMinusOpen	Stroop_CTSIB	Original	Original Control	Band4_Tremor	22	-0.323472	-0.83929	0.16263	5000
ClosedMinusOpen	Stroop_CTSIB	Original	Original post-COVID	Band1_PosturalSway	106	2.448303	-0.307022	5.405476	5000
ClosedMinusOpen	Stroop_CTSIB	Original	Original post-COVID	Band2_Normal	106	-1.801318	-4.546767	0.807574	5000
ClosedMinusOpen	Stroop_CTSIB	Original	Original post-COVID	Band3_Overcomp	106	-0.532469	-1.106471	0.01301	5000
ClosedMinusOpen	Stroop_CTSIB	Original	Original post-COVID	Band4_Tremor	106	-0.114516	-0.277984	0.047367	5000
ClosedMinusOpen	Stroop_CTSIB	Quantum	QUBO post-COVID Control-like	Band1_PosturalSway	64	0.372764	-2.697803	3.490282	5000
ClosedMinusOpen	Stroop_CTSIB	Quantum	QUBO post-COVID Control-like	Band2_Normal	64	0.008893	-2.864369	2.919975	5000
ClosedMinusOpen	Stroop_CTSIB	Quantum	QUBO post-COVID Control-like	Band3_Overcomp	64	-0.30147	-1.121613	0.438332	5000

Continued on next page

Supplementary Table S1 continued.

Delta type	Task contrast	Method	Subgroup	Frequency band	n	Mean ΔEnergy (%)	CI lower	CI upper	Bootstrap n
ClosedMinusOpen	Stroop_CTSIB	Quantum	QUBO post-COVID Control-like	Band4_Tremor	64	-0.080187	-0.244414	0.085863	5000
ClosedMinusOpen	Stroop_CTSIB	Quantum	QUBO post-COVID Control-distant	Band1_PosturalSway	42	5.611029	0.832193	10.757139	5000
ClosedMinusOpen	Stroop_CTSIB	Quantum	QUBO post-COVID Control-distant	Band2_Normal	42	-4.559735	-9.630314	0.347735	5000
ClosedMinusOpen	Stroop_CTSIB	Quantum	QUBO post-COVID Control-distant	Band3_Overcomp	42	-0.884466	-1.606038	-0.2338	5000
ClosedMinusOpen	Stroop_CTSIB	Quantum	QUBO post-COVID Control-distant	Band4_Tremor	42	-0.166828	-0.506493	0.12538	5000
ClosedMinusOpen	Stroop_CTSIB	GMM	GMM post-COVID Control-like	Band1_PosturalSway	14	3.880275	0.350132	7.909649	5000
ClosedMinusOpen	Stroop_CTSIB	GMM	GMM post-COVID Control-like	Band2_Normal	14	-3.666755	-8.05635	0.033826	5000
ClosedMinusOpen	Stroop_CTSIB	GMM	GMM post-COVID Control-like	Band3_Overcomp	14	-0.160567	-0.62737	0.382156	5000
ClosedMinusOpen	Stroop_CTSIB	GMM	GMM post-COVID Control-like	Band4_Tremor	14	-0.052953	-0.235861	0.171282	5000
ClosedMinusOpen	Stroop_CTSIB	GMM	GMM post-COVID Control-distant	Band1_PosturalSway	92	2.230394	-0.889391	5.434063	5000
ClosedMinusOpen	Stroop_CTSIB	GMM	GMM post-COVID Control-distant	Band2_Normal	92	-1.517447	-4.556628	1.499052	5000
ClosedMinusOpen	Stroop_CTSIB	GMM	GMM post-COVID Control-distant	Band3_Overcomp	92	-0.589062	-1.236558	0.025786	5000
ClosedMinusOpen	Stroop_CTSIB	GMM	GMM post-COVID Control-distant	Band4_Tremor	92	-0.123885	-0.310872	0.048426	5000
ClosedMinusOpen	Stroop_Romberg	Original	Original Control	Band1_PosturalSway	23	2.299025	-5.985225	9.867607	5000
ClosedMinusOpen	Stroop_Romberg	Original	Original Control	Band2_Normal	23	-0.597292	-6.296789	5.593722	5000
ClosedMinusOpen	Stroop_Romberg	Original	Original Control	Band3_Overcomp	23	-1.583859	-4.373862	1.236153	5000
ClosedMinusOpen	Stroop_Romberg	Original	Original Control	Band4_Tremor	23	-0.117874	-0.662929	0.445076	5000
ClosedMinusOpen	Stroop_Romberg	Original	Original post-COVID	Band1_PosturalSway	108	1.71202	-1.159424	4.697021	5000
ClosedMinusOpen	Stroop_Romberg	Original	Original post-COVID	Band2_Normal	108	-1.03525	-3.749737	1.644701	5000
ClosedMinusOpen	Stroop_Romberg	Original	Original post-COVID	Band3_Overcomp	108	-0.469124	-1.161699	0.138939	5000
ClosedMinusOpen	Stroop_Romberg	Original	Original post-COVID	Band4_Tremor	108	-0.207646	-0.380176	-0.055906	5000
ClosedMinusOpen	Stroop_Romberg	Quantum	QUBO post-COVID Control-like	Band1_PosturalSway	64	1.920757	-1.880254	5.639882	5000
ClosedMinusOpen	Stroop_Romberg	Quantum	QUBO post-COVID Control-like	Band2_Normal	64	-1.56327	-5.024075	2.026251	5000
ClosedMinusOpen	Stroop_Romberg	Quantum	QUBO post-COVID Control-like	Band3_Overcomp	64	-0.245237	-0.81236	0.47971	5000
ClosedMinusOpen	Stroop_Romberg	Quantum	QUBO post-COVID Control-like	Band4_Tremor	64	-0.11225	-0.266319	0.033335	5000
ClosedMinusOpen	Stroop_Romberg	Quantum	QUBO post-COVID Control-distant	Band1_PosturalSway	44	1.408404	-3.279085	6.257336	5000
ClosedMinusOpen	Stroop_Romberg	Quantum	QUBO post-COVID Control-distant	Band2_Normal	44	-0.267222	-4.492958	4.160937	5000
ClosedMinusOpen	Stroop_Romberg	Quantum	QUBO post-COVID Control-distant	Band3_Overcomp	44	-0.794778	-2.202807	0.305142	5000

Continued on next page

Supplementary Table S1 continued.

Delta type	Task contrast	Method	Subgroup	Frequency band	n	Mean ΔEnergy (%)	CI lower	CI upper	Bootstrap n
ClosedMinusOpen	Stroop_Romberg	Quantum	QUBO post-COVID Control-distant	Band4_Tremor	44	-0.346404	-0.703346	-0.023544	5000
ClosedMinusOpen	Stroop_Romberg	GMM	GMM post-COVID Control-like	Band1_PosturalSway	14	-1.645505	-6.2725	2.77285	5000
ClosedMinusOpen	Stroop_Romberg	GMM	GMM post-COVID Control-like	Band2_Normal	14	1.989782	-1.885936	6.29812	5000
ClosedMinusOpen	Stroop_Romberg	GMM	GMM post-COVID Control-like	Band3_Overcomp	14	-0.189547	-0.968554	0.649405	5000
ClosedMinusOpen	Stroop_Romberg	GMM	GMM post-COVID Control-like	Band4_Tremor	14	-0.154731	-0.516048	0.217106	5000
ClosedMinusOpen	Stroop_Romberg	GMM	GMM post-COVID Control-distant	Band1_PosturalSway	94	2.212077	-1.031445	5.600366	5000
ClosedMinusOpen	Stroop_Romberg	GMM	GMM post-COVID Control-distant	Band2_Normal	94	-1.485787	-4.608241	1.586535	5000
ClosedMinusOpen	Stroop_Romberg	GMM	GMM post-COVID Control-distant	Band3_Overcomp	94	-0.510763	-1.255614	0.195069	5000
ClosedMinusOpen	Stroop_Romberg	GMM	GMM post-COVID Control-distant	Band4_Tremor	94	-0.215527	-0.405818	-0.04058	5000
ClosedMinusOpen	Stroop_Tandem_Romberg	Original	Original Control	Band1_PosturalSway	24	-4.609951	-12.214978	2.978838	5000
ClosedMinusOpen	Stroop_Tandem_Romberg	Original	Original Control	Band2_Normal	24	3.37889	-1.227222	8.612323	5000
ClosedMinusOpen	Stroop_Tandem_Romberg	Original	Original Control	Band3_Overcomp	24	1.182994	-4.042104	5.971004	5000
ClosedMinusOpen	Stroop_Tandem_Romberg	Original	Original Control	Band4_Tremor	24	0.048067	-1.01302	1.010733	5000
ClosedMinusOpen	Stroop_Tandem_Romberg	Original	Original post-COVID	Band1_PosturalSway	108	1.659454	-1.546042	4.84817	5000
ClosedMinusOpen	Stroop_Tandem_Romberg	Original	Original post-COVID	Band2_Normal	108	-1.905062	-4.191457	0.315088	5000
ClosedMinusOpen	Stroop_Tandem_Romberg	Original	Original post-COVID	Band3_Overcomp	108	0.052309	-1.758151	1.994544	5000
ClosedMinusOpen	Stroop_Tandem_Romberg	Original	Original post-COVID	Band4_Tremor	108	0.193299	-0.256947	0.64892	5000
ClosedMinusOpen	Stroop_Tandem_Romberg	Quantum	QUBO post-COVID Control-like	Band1_PosturalSway	64	0.766554	-3.034776	4.354207	5000
ClosedMinusOpen	Stroop_Tandem_Romberg	Quantum	QUBO post-COVID Control-like	Band2_Normal	64	0.61023	-1.885599	3.374904	5000
ClosedMinusOpen	Stroop_Tandem_Romberg	Quantum	QUBO post-COVID Control-like	Band3_Overcomp	64	-1.195428	-3.386799	0.986513	5000
ClosedMinusOpen	Stroop_Tandem_Romberg	Quantum	QUBO post-COVID Control-like	Band4_Tremor	64	-0.181355	-0.759247	0.351346	5000
ClosedMinusOpen	Stroop_Tandem_Romberg	Quantum	QUBO post-COVID Control-distant	Band1_PosturalSway	44	2.958216	-2.786205	8.815266	5000
ClosedMinusOpen	Stroop_Tandem_Romberg	Quantum	QUBO post-COVID Control-distant	Band2_Normal	44	-5.563668	-9.198372	-2.117355	5000
ClosedMinusOpen	Stroop_Tandem_Romberg	Quantum	QUBO post-COVID Control-distant	Band3_Overcomp	44	1.867201	-1.328875	5.380419	5000

Continued on next page

Supplementary Table S1 continued.

Delta type	Task contrast	Method	Subgroup	Frequency band	n	Mean ΔEnergy (%)	CI lower	CI upper	Bootstrap n
ClosedMinusOpen	Stroop_Tandem_Romberg	Quantum	QUBO post-COVID Control-distant	Band4_Tremor	44	0.738251	0.095737	1.507637	5000
ClosedMinusOpen	Stroop_Tandem_Romberg	GMM	GMM post-COVID Control-like	Band1_PosturalSway	15	-5.505968	-13.843085	1.866115	5000
ClosedMinusOpen	Stroop_Tandem_Romberg	GMM	GMM post-COVID Control-like	Band2_Normal	15	0.290446	-3.361939	4.46717	5000
ClosedMinusOpen	Stroop_Tandem_Romberg	GMM	GMM post-COVID Control-like	Band3_Overcomp	15	4.596145	-0.30286	10.730305	5000
ClosedMinusOpen	Stroop_Tandem_Romberg	GMM	GMM post-COVID Control-like	Band4_Tremor	15	0.619377	-0.327685	2.00607	5000
ClosedMinusOpen	Stroop_Tandem_Romberg	GMM	GMM post-COVID Control-distant	Band1_PosturalSway	93	2.815167	-0.685285	6.278894	5000
ClosedMinusOpen	Stroop_Tandem_Romberg	GMM	GMM post-COVID Control-distant	Band2_Normal	93	-2.259176	-4.699958	0.245501	5000
ClosedMinusOpen	Stroop_Tandem_Romberg	GMM	GMM post-COVID Control-distant	Band3_Overcomp	93	-0.680567	-2.627877	1.302179	5000
ClosedMinusOpen	Stroop_Tandem_Romberg	GMM	GMM post-COVID Control-distant	Band4_Tremor	93	0.124577	-0.357132	0.616018	5000

Supplementary Table S2

Supplementary Table S2

Supplementary Table S2 reports the contrast-level comparison between the quantum-annealing sampled QUBO assignments and the simple Control-distance threshold baseline. The threshold baseline used the 95th percentile of normalized Control-distance scores among Control participants as a contrast-specific threshold. Participants with Control-distance scores greater than this threshold were assigned to the Control-distant group. Agreement was computed across all participant–contrast assignments available for each contrast, and the Jaccard index was computed using the Control-distant assignment sets. The Jaccard index therefore quantifies the overlap between the Control-distant assignment set obtained from the quantum-annealing sampled QUBO solution and the Control-distant assignment set obtained from the threshold baseline.

Table S2: Control-distance threshold baseline comparison with quantum-annealing sampled QUBO assignments.

Task contrast	n	QA Control-distant	Threshold Control-distant	Agreement	Jaccard index	Threshold Control	Threshold Post-COVID
$\Delta S_{\mathrm{dual-single}}$: CTSIB (Closed Eyes)	123	11	10	92.7%	0.400	2/22	8/101
$\Delta S_{\mathrm{dual-single}}$: CTSIB (Open Eyes)	127	20	29	92.9%	0.690	2/23	27/104
$\Delta S_{\mathrm{dual-single}}$: Fukuda	125	5	15	92.0%	0.333	2/24	13/101
$\Delta S_{\mathrm{V,single}}$: CTSIB	131	8	11	96.2%	0.583	2/24	9/107
$\Delta S_{\mathrm{V,single}}$: Romberg	130	6	9	94.6%	0.364	2/25	7/105
$\Delta S_{\mathrm{V,single}}$: Tandem Romberg	130	4	11	94.6%	0.364	2/25	9/105
$\Delta S_{\mathrm{dual-single}}$: Romberg (Closed Eyes)	132	10	20	92.4%	0.500	2/24	18/108
$\Delta S_{\mathrm{dual-single}}$: Romberg (Open Eyes)	126	7	19	90.5%	0.368	2/23	17/103
$\Delta S_{\mathrm{V,dual}}$: CTSIB	128	11	14	96.1%	0.667	2/22	12/106
$\Delta S_{\mathrm{V,dual}}$: Romberg	131	10	17	93.1%	0.500	2/23	15/108
$\Delta S_{\mathrm{V,dual}}$: Tandem Romberg	132	10	16	95.5%	0.625	2/24	14/108
$\Delta S_{\mathrm{dual-single}}$: Tandem Romberg (Closed Eyes)	128	10	21	91.4%	0.476	2/24	19/104
$\Delta S_{\mathrm{dual-single}}$: Tandem Romberg (Open Eyes)	130	8	13	96.2%	0.615	2/24	11/106
All contrasts	1673	119	205	93.7%	0.512	26/307	179/1366

Note: The Jaccard index reports the overlap between the Control-distant assignment set obtained from the quantum-annealing sampled QUBO solution and the Control-distant assignment set obtained from the threshold baseline.

Supplementary Table S3

Supplementary Table S3 reports the achieved BQM energy for the quantum-annealing sampled solution, simulated annealing, and tabu search for each balance-derived task contrast. Because all three solvers were applied to the same contrast-specific BQM/QUBO objective, achieved energy provides a direct solver-level measure of solution quality. Energy values are objective-specific and should not be interpreted as physiological severity scores across task contrasts.

Table S3: Solver-level achieved BQM energies across balance-derived task contrasts.

Task contrast	QA sampled energy	SA best energy	Tabu best energy
$\Delta S_{\text{dual}-\text{single}}$: Romberg (Open Eyes)	−9.72	−12.711	−12.711
$\Delta S_{\text{dual}-\text{single}}$: Romberg (Closed Eyes)	1.70	−14.569	−14.569
$\Delta S_{\text{dual}-\text{single}}$: Tandem Romberg (Open Eyes)	−12.66	−18.117	−18.117
$\Delta S_{\text{dual}-\text{single}}$: Tandem Romberg (Closed Eyes)	−17.13	−21.128	−21.128
$\Delta S_{\text{dual}-\text{single}}$: CTSIB (Open Eyes)	−11.43	−27.303	−27.303
$\Delta S_{\text{dual}-\text{single}}$: CTSIB (Closed Eyes)	−8.96	−15.029	−15.029
$\Delta S_{\text{dual}-\text{single}}$: Fukuda	−10.75	−10.751	−10.751
$\Delta S_{\text{V,single}}$: Romberg	−8.40	−14.480	−14.480
$\Delta S_{\text{V,single}}$: Tandem Romberg	−0.89	−13.810	−13.810
$\Delta S_{\text{V,single}}$: CTSIB	−7.56	−17.847	−17.847
$\Delta S_{\text{V,dual}}$: Romberg	−5.32	−16.483	−16.483
$\Delta S_{\text{V,dual}}$: Tandem Romberg	−9.12	−19.485	−19.485
$\Delta S_{\text{V,dual}}$: CTSIB	−4.66	−17.871	−17.871

Supplementary Table S4

Supplementary Table S4 reports the QUBO parameter-sensitivity settings and assignment agreement relative to the corresponding baseline configuration. Overall binary assignment agreement was computed across all participant–contrast assignments. The Jaccard index was computed using only the Control-distant assignment sets and therefore focuses on the smaller outcome subset of primary interest.

Table S4: QUBO parameter-sensitivity settings and assignment stability relative to baseline.

Solver	Configuration	k	λ_{anchor}	λ_{sparse}	λ_{outlier}	λ_{smooth}	Baseline Control-distant	Config Control-distant	Overall agreement	Jaccard index
**(a) Representative QPU sensitivity analysis**										
QPU	baseline	32	8.0	1.0	3.0	2.0	123	123	100.0%	1.000
QPU	`anchor_low`	32	6.0	1.0	3.0	2.0	123	125	95.3%	0.521
QPU	`anchor_high`	32	10.0	1.0	3.0	2.0	123	117	95.3%	0.509
QPU	`outlier_low`	32	8.0	1.0	2.0	2.0	123	99	95.1%	0.461
QPU	`outlier_high`	32	8.0	1.0	4.0	2.0	123	124	94.9%	0.488
QPU	`knn_24`	24	8.0	1.0	3.0	2.0	123	154	93.6%	0.443
QPU	`knn_40`	40	8.0	1.0	3.0	2.0	123	108	95.3%	0.490
**(b) Simulated-annealing sensitivity analysis**										
SA	baseline	32	8.0	1.0	3.0	2.0	143	143	100.0%	1.000
SA	`anchor_low`	32	6.0	1.0	3.0	2.0	143	143	100.0%	1.000
SA	`anchor_high`	32	10.0	1.0	3.0	2.0	143	143	100.0%	1.000
SA	`sparse_low`	32	8.0	0.5	3.0	2.0	143	152	99.5%	0.941
SA	`sparse_high`	32	8.0	1.5	3.0	2.0	143	134	99.5%	0.937
SA	`outlier_low`	32	8.0	1.0	2.0	2.0	143	124	98.9%	0.867
SA	`outlier_high`	32	8.0	1.0	4.0	2.0	143	153	99.4%	0.935
SA	`smooth_low`	32	8.0	1.0	3.0	1.0	143	156	99.2%	0.917
SA	`smooth_high`	32	8.0	1.0	3.0	3.0	143	134	99.5%	0.937
SA	`knn_24`	24	8.0	1.0	3.0	2.0	143	171	98.3%	0.836
SA	`knn_40`	40	8.0	1.0	3.0	2.0	143	123	98.8%	0.860

Note. The Jaccard index reports the overlap between the Control-distant assignment set obtained from each parameter configuration and the Control-distant assignment set obtained from the corresponding baseline configuration.
